# A unified nomenclature for vertebrate olfactory receptors

**DOI:** 10.1186/s12862-020-01607-6

**Published:** 2020-04-15

**Authors:** Tsviya Olender, Tamsin E. M. Jones, Elspeth Bruford, Doron Lancet

**Affiliations:** 1 Department of Molecular Genetics, Weizmann Institute of Science, 76100 Rehovot, Israel; 2 HUGO Gene Nomenclature Committee, European Molecular Biology Laboratory, European Bioinformatics Institute, Wellcome Genome Campus, Hinxton, Cambridgeshire, CB10 1SD UK; 3 Department of Haematology, University of Cambridge School of Clinical Medicine, Cambridge Biomedical Campus, Cambridge, CB2 0AW UK

**Keywords:** Olfaction, Nomenclature, Olfactory receptors, Orthologs, Paralogs, Evolution

## Abstract

**Background:**

Olfactory receptors (ORs) are G protein-coupled receptors with a crucial role in odor detection. A typical mammalian genome harbors ~ 1000 OR genes and pseudogenes; however, different gene duplication/deletion events have occurred in each species, resulting in complex orthology relationships. While the human OR nomenclature is widely accepted and based on phylogenetic classification into 18 families and further into subfamilies, for other mammals different and multiple nomenclature systems are currently in use, thus concealing important evolutionary and functional insights.

**Results:**

Here, we describe the Mutual Maximum Similarity (MMS) algorithm, a systematic classifier for assigning a human-centric nomenclature to any OR gene based on inter-species hierarchical pairwise similarities. MMS was applied to the OR repertoires of seven mammals and zebrafish. Altogether, we assigned symbols to 10,249 ORs. This nomenclature is supported by both phylogenetic and synteny analyses. The availability of a unified nomenclature provides a framework for diverse studies, where textual symbol comparison allows immediate identification of potential ortholog groups as well as species-specific expansions/deletions; for example, *Or52e5* and *Or52e5b* represent a rat-specific duplication of *OR52E5*. Another example is the complete absence of OR subfamily OR6Z among primate OR symbols. In other mammals, OR6Z members are located in one genomic cluster, suggesting a large deletion in the great ape lineage. An additional 14 mammalian OR subfamilies are missing from the primate genomes. While in chimpanzee 87% of the symbols were identical to human symbols, this number decreased to ~ 50% in dog and cow and to ~ 30% in rodents, reflecting the adaptive changes of the OR gene superfamily across diverse ecological niches. Application of the proposed nomenclature to zebrafish revealed similarity to mammalian ORs that could not be detected from the current zebrafish olfactory receptor gene nomenclature.

**Conclusions:**

We have consolidated a unified standard nomenclature system for the vertebrate OR superfamily. The new nomenclature system will be applied to cow, horse, dog and chimpanzee by the Vertebrate Gene Nomenclature Committee and its implementation is currently under consideration by other relevant species-specific nomenclature committees.

## Background

Olfactory receptors (ORs) are G protein-coupled receptors with an essential role in odor detection. Being the largest gene family in vertebrates, a typical mammalian genome harbors ~ 1000 OR genes and pseudogenes; however, the numbers of functional OR genes vary enormously among genomes of different animals, reflecting the adaptation of organisms to different environments [1–4]. ORs are distributed in clusters on most mammalian chromosomes. There are strong indications of a common ancestry for most OR clusters [5], a feature which might be related to common cis-regulatory elements [6, 7]. Nevertheless, processes of gene duplication and gene deletion have taken place in each species [1–4], making orthology relationships difficult to determine [8], and hence requiring careful manual curation for the assignment of gene nomenclature. Another complexity arises from the high content of OR pseudogenes in some organisms, such as human (55% pseudogenes) [9]. Thus, assigning orthology-based symbols to the OR gene superfamily is challenging and requires conceptual translation of all OR pseudogenes.

For many years, an official nomenclature system has been in place for human ORs [10] that is widely accepted and utilized by the community. The human nomenclature is based on a sequence similarity classification of the OR repertoire into 18 families and > 300 subfamilies, where symbols consist of the root “OR” followed by a family numeral, subfamily letter(s), and a numeral representing the individual gene within the subfamily. For example, *OR3A1* is member 1 of family 3, subfamily A, and *OR7E12P* is an OR pseudogene that is member 12 of family 7, subfamily E. The details of the classification method were previously described [9–12]. Briefly, a new gene is classified into the same subfamily if it shows at least 60% protein sequence identity to the best hit; family membership is based on at least 40% sequence identity at the protein level. This classification system is based on a divergent evolutionary model of the ORs [10, 13, 14] derived from phylogenetic analyses of ORs from multiple species [10]. It is further consistent with accepted nomenclature schemes for other multigene families, e.g. the cytochrome P450 superfamily [15, 16] and the UDP glucuronosyltransferase superfamily [17], which use similar cutoffs. This nomenclature has already been applied to dog, platypus and opossum [5, 18, 19] and is available to the community via a dedicated database, the Human Olfactory Receptor Data Explorer (HORDE) [11, 20, 21].

In some other vertebrates, different nomenclature systems are currently in use. For example, several nomenclature systems have previously been published for mouse. One utilized the prefix ‘MOR’, followed by a family number, a hyphen (−) and a number representing the individual gene within the family, with ‘P’ at the end to denote a pseudogene (e.g. *MOR1–1* and *MOR185-9P*) [22]; another was based on genomic location [23]. The current Mouse Genome Informatics (MGI) official nomenclature uses the root ‘Olfr’ followed by a number, with ‘-ps’ and another number at the end for pseudogenes (e.g. *Olfr562* and *Olfr1001-ps1*). This serial number-based nomenclature conceals important structural and functional insights. Moreover, it deviates from the rule applied to almost all other genes, whereby, when possible, the symbols for orthologous genes in human and other vertebrates should be identical (excluding capitalization). Another example of deviation from this rule is the zebrafish OR nomenclature, which is based on a phylogenetic classification into subfamilies with sequential numbers starting from 101 (e.g. *or101–1* and *or102–1* are two genes in two different subfamilies) [24]. As fish ORs are highly diverged [25], the designated OR subfamilies in fish are expected to be lineage-specific and, therefore, this nomenclature [24] is not useful to detect similarities across fish and vertebrates.

The use of different nomenclature in different organisms creates difficulties both when comparing genes across species and, especially, when the same gene is reported more than once under different names. Although this situation is also common with other genes, it becomes especially confusing in large gene families that are found in multiple species, such as the ORs. Next generation technologies are dramatically increasing the number of sequenced vertebrates. Therefore, the availability of a unified and widely accepted nomenclature that encodes homology relationships becomes more important than ever.

Here, we propose a unified nomenclature system for vertebrate OR genes and pseudogenes. The nomenclature is human-centric and therefore based on the human classification system for OR genes. Using a dedicated algorithm (Mutual Maximum Similarity, MMS), we applied our nomenclature system to the OR repertoires of mouse, rat, cow, dog, horse, orangutan and chimpanzee and, finally, also to zebrafish, a more distantly related vertebrate species. We show that the nomenclature captures the phylogenetic relationships among the studied species and provides a powerful framework for diverse studies of vertebrate ORs. A unified nomenclature for the OR gene family can also serve as a model for other large multigene families, allowing researchers to easily make cross-species comparisons in complex groups of genes. All of the nomenclature data are available from the HORDE database (https://genome.weizmann.ac.il/horde/), and are under consideration by the relevant species-specific nomenclature committees that are using an alternative OR nomenclature, namely MGNC for mouse [26], RGNC for rat [27] and ZNC for zebrafish [28]. The Vertebrate Gene Nomenclature Committee (VGNC) [29] are currently naming genes within chimpanzee, cow, dog and horse and will adopt this OR nomenclature in these species.

## Results

### The MMS algorithm

**Fig. 1:**
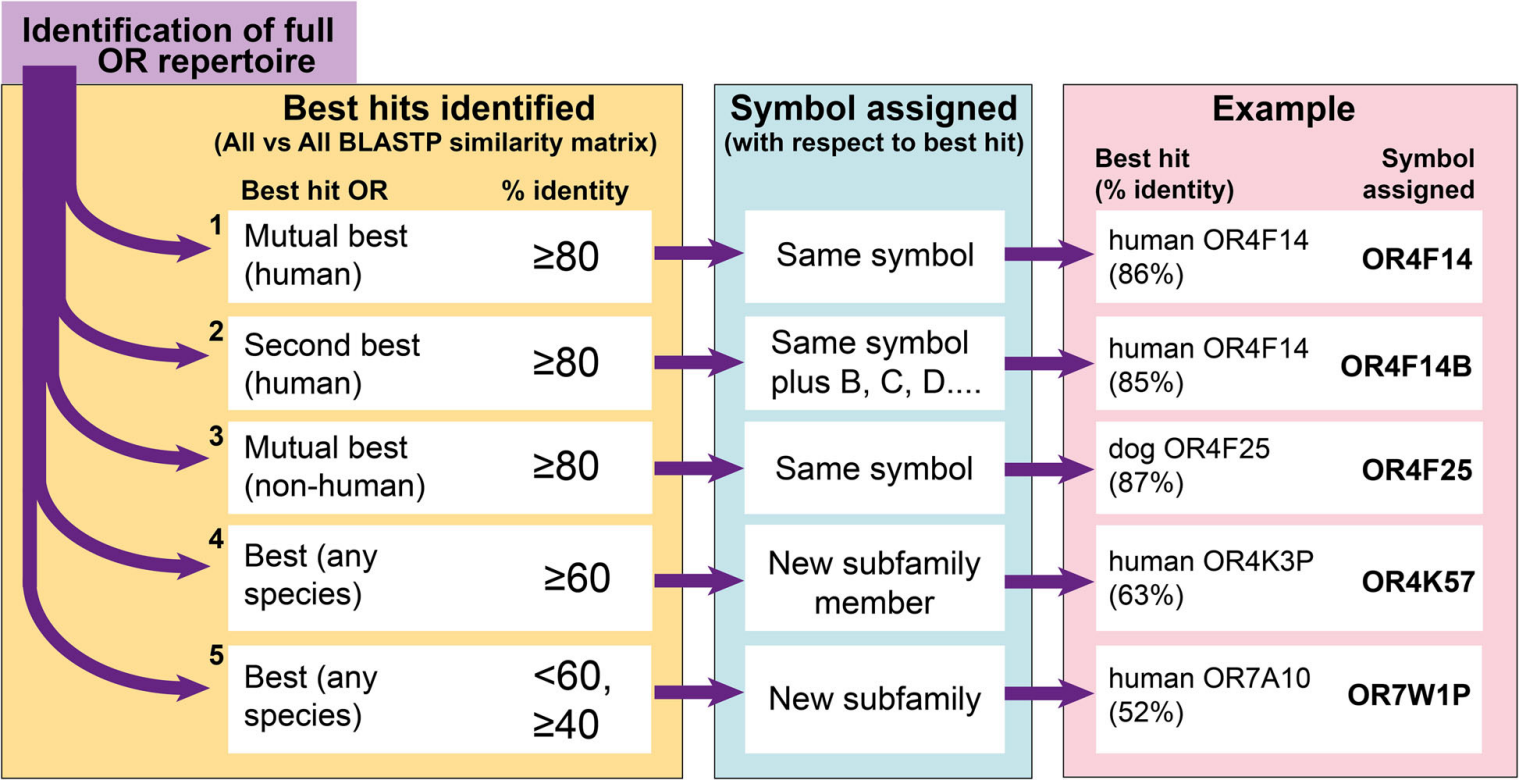
The MMS algorithm. A diagram illustrating the workflow of hierarchical symbol assignment with the MMS algorithm. After identification of the full OR repertoire in a given species, all vs all BLASTP similarity matrices are generated against the OR repertoires of human and other mammals whose repertoires have been classified. The classification criteria (highlighted in the left-hand yellow box), numbered 1–5, are applied in a stepwise fashion: genes that fail to pass the cutoff in step 1 are passed to step 2, and so on. Symbols are assigned based on the best hits as shown in the middle (blue) box. Examples of cow OR symbols at each classification step are shown in the right-hand (pink) box

The MMS algorithm assigns human-based OR symbols by detecting the inter-species hierarchical pairwise similarities (Fig. 1). The algorithm first analyzes the all-versus-all BLASTP identity matrix of the given OR repertoire versus human. Mutual best hits with ≥ 80% identity are first identified and are assigned the same symbol as the human best hit, second-best ortholog candidates with ≥ 80% identity are then identified and are assigned with the human best hit gene symbol with the addition of the letters *B*, *C*, etc., with the exception of *P* which is reserved for pseudogenes. For example, *OR9A4* is the mutual best hit and *OR9A4B* is the second best hit. The ≥ 80% identity cutoff is based on our previous studies of sequence similarities between mutual best hits across mammals [5, 11], and on phylogenetic analyses that we performed on a randomly selected set of OR subfamilies during the current project (data not shown). We previously reported a high level of conservation of ORs across mammals [5, 30], and thus we believe that this cutoff can be used for most placental mammals. The remaining ORs are compared to non-human ORs to detect non-human OR orthology relationships. OR genes that have not already been named in the above steps are classified into families and subfamilies in the same way as the human ORs [10, 11] and as shown in Fig. 1. We use a ≥ 60% identity cutoff to classify a gene as a new member of a subfamily with the next available subfamily member number. If the sequence identity is below 60% we use ≥40% identity to classify the gene into an OR family, with a novel subfamily symbol (see above, and also in Olender T. et al. 2013 [11] for more details). At the step of subfamily classification (Step 4, Fig. 1), the similarity matrix includes the within-species OR repertoire in addition to the other species’ OR repertoires. This step is required for the correct classification of species-specific subfamilies. An exception to the above order of comparisons was made for the rat, where the repertoire was initially compared to mouse before detecting best human matches, to take into account the close evolutionary distance of the rodents. Symbols are composed of uppercase letters and Arabic numbers, except in rodents where, by convention, only the first letter is capitalized and the suffix “*-ps*” is used for pseudogenes in place of “*P*”.

### Classification of mammalian OR repertoires

We used the MMS algorithm to assign symbols to the OR repertoires of the mouse, rat, dog, cow, horse, chimpanzee and orangutan (Table 1). Across all 7 mammalian species, the OR genes and pseudogenes were classified into 18 families and 623 subfamilies (Additional file 1, Additional file 2). A comparison between our subfamily classification and the orthologous gene groups (OGGs) suggested by Niimura [31] for the cow, dog and horse genes found in both studies (4587 loci) shows that the OGG method classifies the ORs into smaller subgroups (737 OGGs versus 285 OR subfamilies), where a typical OR subfamily includes on average 2.6 OGGs.

**Table 1:** Numbers of identified OR genes and pseudogenes for each species, including the version of the reference genome that was used in the data-mining step (see Methods)

Species	Genes	Pseudogenes	Total	Genome
chimpanzee	396	427	823	PanTro4
orangutan	321	466	787	PonAbe2
dog	803	206	1009	CanFam3
cow	1110	695	1805	BosTau8
horse	1101	1372	2473	EquCab2
mouse	1142	247	1389	Mm10
rat	1333	457	1790	Rn6
zebrafish	158	14	172	DanRer10
**Total**	**6365**	**3884**	**10,249**	

The fraction of genes and pseudogenes assigned as putative orthologs of a human gene (including those with the B/C/D suffix) varies from 83.9% in chimpanzee to ~ 27% in rodents (Additional file 3: Fig. S1). These numbers are in line with the literature and reflect the rapid evolution of the OR gene family [8, 23], including the ~ 20% difference between human and chimpanzee OR repertoires we found, which is similar to the ~ 25% reported by Go [32]. As expected, genes whose symbols are shared among more than one mammal contain a significantly higher fraction of intact ORs, as compared to genes that were assigned as novel subfamily members (Additional file 3: Fig. S2).

To test if genes assigned as best and second best hits to a human OR gene also lie in syntenic regions, we analyzed mammalian multiz genome alignments [33]. We found that 88% of the mutual best hits and 62% of the second best hits aligned to the exact genomic location of the corresponding human ortholog, and 96% of the mutual-best hits and 82% of the second best are aligned within a distance of < 100 kb. Thus, most of the ORs that were classified by the MMS algorithm as putative orthologs also reside in the approximate expected syntenic location. We note that due to the rapid evolution of the OR gene family synteny is not always expected to be preserved among orthologs.

**Fig. 2:**
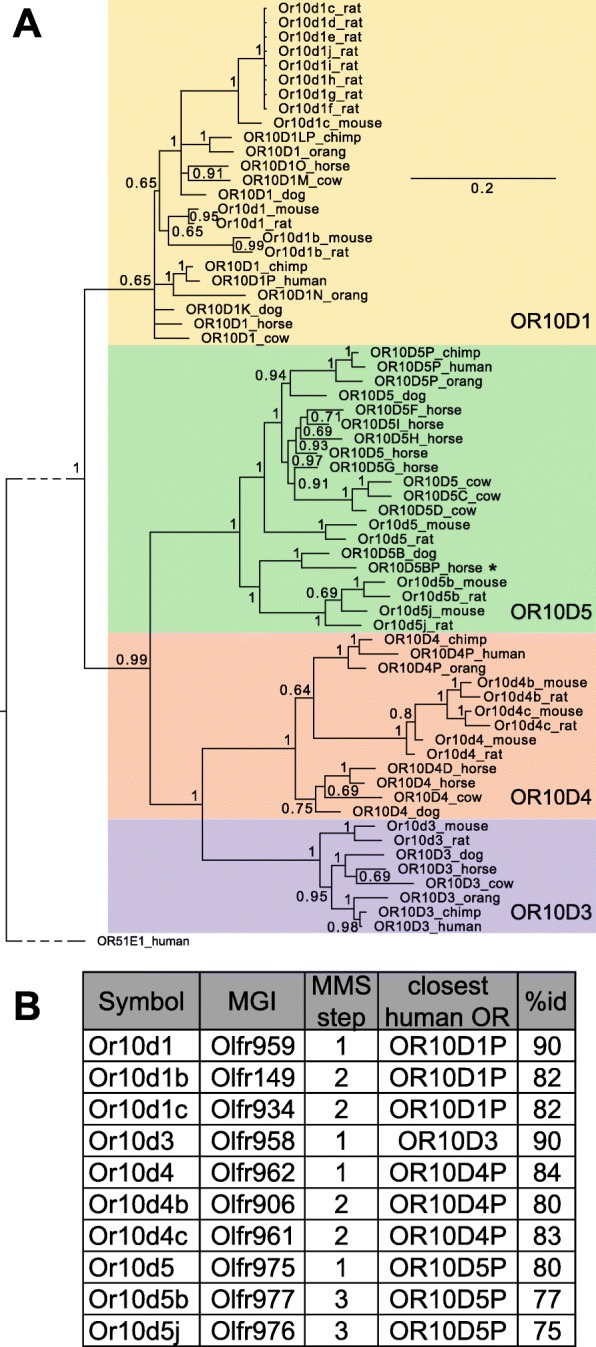
OR subfamily 10D classification. **a** Bayesian phylogeny of subfamily 10D members. Human OR51E1 was used as the outgroup. The tree is displayed with posterior probabilities on the branch nodes. Scale bar represents the number of amino acid changes per site. The dotted line indicates that that the outgroup branch has been compressed for display purposes; the unmodified branch length can be viewed in Additional file 5. The subfamily member clades are each given a different background colors. OR10D1: yellow, OR10D3: purple, OR10D4: orange, and OR10D5: green. An asterisk denotes a gene that was classified manually based on the strength of phylogenetic evidence placing it within that clade. Automated symbol assignment using the MMS algorithm is concordant with the clades resolved by phylogenetic analysis. **b** Details of the classification of mouse OR10D genes. For each gene, the table shows the symbol suggested using the MMS algorithm alongside the MMS classification step in which it was assigned (see Fig. 1), the current MGI symbol, and its closest human OR gene (by % identity)

We used phylogenetic analysis to assess the accuracy of the nomenclature assignment. Though, in general, phylogenetic analysis cannot fully resolve the relationships within the OR superfamily due to low bootstrap values (also reported by Niimura [34], Niimura [35], Rimbault [36] and Khan [37]) this is possible within subfamilies, as shown in Fig. 2a. The relationships between the genes in subfamily 10D can immediately be recognized from the phylogenetic tree as well as from the symbols (Fig. 2a). These relationships would not be easily detected using, for example, the current approved mouse gene nomenclature (Fig. 2b). For each of the 50 largest OR subfamilies, representing 45% of the total number of ORs in these species, we generated Maximum Likelihood and Bayesian phylogenies (Additional files 4 and 5). We then compared the within-subfamily groupings in these phylogenies against the hierarchical naming results. We found that the nomenclature assignments were largely congruent with the phylogenetic groupings of ORs within a subfamily, and 195 exceptions for which there was strong phylogenetic support for reclassification by both maximum likelihood and Bayesian inference trees were renamed manually (see Additional files 4,5 and 6). We note that the majority of the ORs that were renamed manually have similarity scores just short of a cut-off that would have led them to be named in line with the phylogeny. Although it is inevitable that rigid similarity cut-offs will occasionally result in this type of classification issue, we provide evidence that the vast majority of OR genes are classified in agreement with phylogenetic analysis and it is mostly pseudogenized loci that are incorrectly classified. We manually updated a very small proportion of the total OR symbols in this study to ensure that the classification is as accurate as possible in this initial cohort of species. This will ensure that when more species’ OR repertoires are classified using the MMS algorithm, the set of ORs that they are being compared to are classified correctly. We performed phylogenetic analysis only within subfamilies, and not in larger family groupings, because it is well established in the literature that due to rapid evolution within the OR superfamily, deeper nodes in OR phylogenies are extremely challenging to resolve with confidence [34–37]. Because of the relative difficulty in assigning deeper family and subfamily relationships using phylogenetic analysis, the MMS method is preferable to phylogenetic classification because it is able to rapidly classify ORs into families with a reproducible and consistent methodology. Nevertheless, as this work is ongoing we note that updates to the genome assemblies of the studied organisms, and future refinements to the MMS algorithm, may result in minor changes to some symbols prior to final approval by nomenclature committees.

The overall congruence of our MMS nomenclature assignment algorithm with both synteny comparison across species, and phylogenetic analyses within OR subfamilies, is highly supportive of our methodology for easily, rapidly and accurately classifying newly identified OR repertoires in a given species.

### Systematic gene family nomenclature as a tool for evolutionary studies

**Fig. 3:**
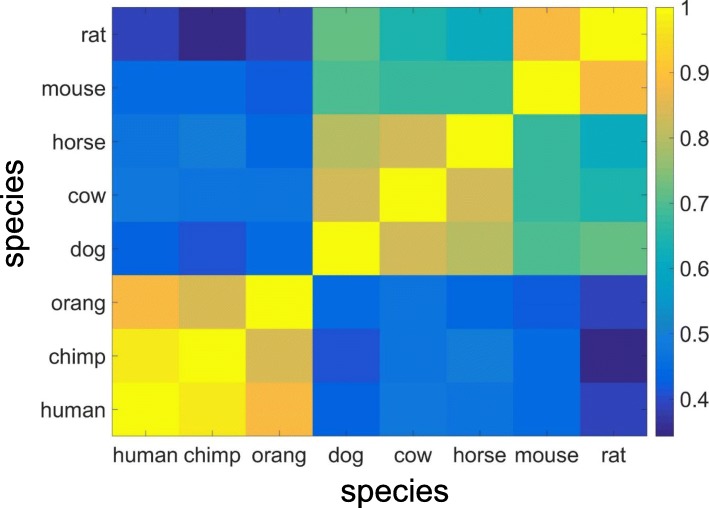
Correlation among the mammalian OR repertoires. The Pearson correlation matrix was calculated using the number of members of each subfamily for each pair of species (Additional file 2). Only subfamilies that are shared by at least 2 mammals (378 subfamilies) were used in the analysis. As expected, the OR repertoires of closely related species show a higher correlation than those of less closely related species

The unified nomenclature system presented here provides a framework for evolutionary studies of vertebrate ORs. We used our subfamily classification to calculate the Pearson correlations coefficients between the classified OR repertoires from each species, using the number of genes in each subfamily (Fig. 3, Additional file 2). The result is in line with evolutionary expectations, where closely related species, namely primates (human, chimpanzee, orangutan), *Laurasiatheria* (dog and cow), and rodents (mouse and rat) are clustered together. We observed that the human and chimpanzee subfamily classifications are closer (0.98 Pearson) than those of mouse and rat (0.90 Pearson). This is expected given that human and chimpanzee diverged ~ 6–12 million years ago [38, 39], whereas mouse and rat diverged ~ 12–24 million years ago [40].

This nomenclature classification also allows the immediate identification of species-specific expansions and deletions. For example, in the species studied the OR4D subfamily has up to 18 members, except for in horse where it has 57. Horse OR4D genes are found within 18 clusters, where the largest cluster contains 17 genes. In total, we identified 147 subfamilies for which the gene count in one of the studied organisms was at least double that in the other organisms, presumably representing species-specific expansions (Additional file 7: Table S1A). The OR subfamily 6Z is entirely absent from the primate genomes analyzed, while in other mammals members of this subfamily lie in a single genomic cluster, suggesting a large deletion in the primate lineage. Ten other mammalian OR subfamilies were not identified in the primate genomes studied, of which six are encoded from a single genomic cluster in the mammalian genome, as is subfamily 6Z (Additional file 7: Table S1B), and an additional 14 subfamilies were not identified in the rodents, of which 8 are encoded in a single genomic cluster (additional file 7: Table S1 B).

**Fig. 4:**
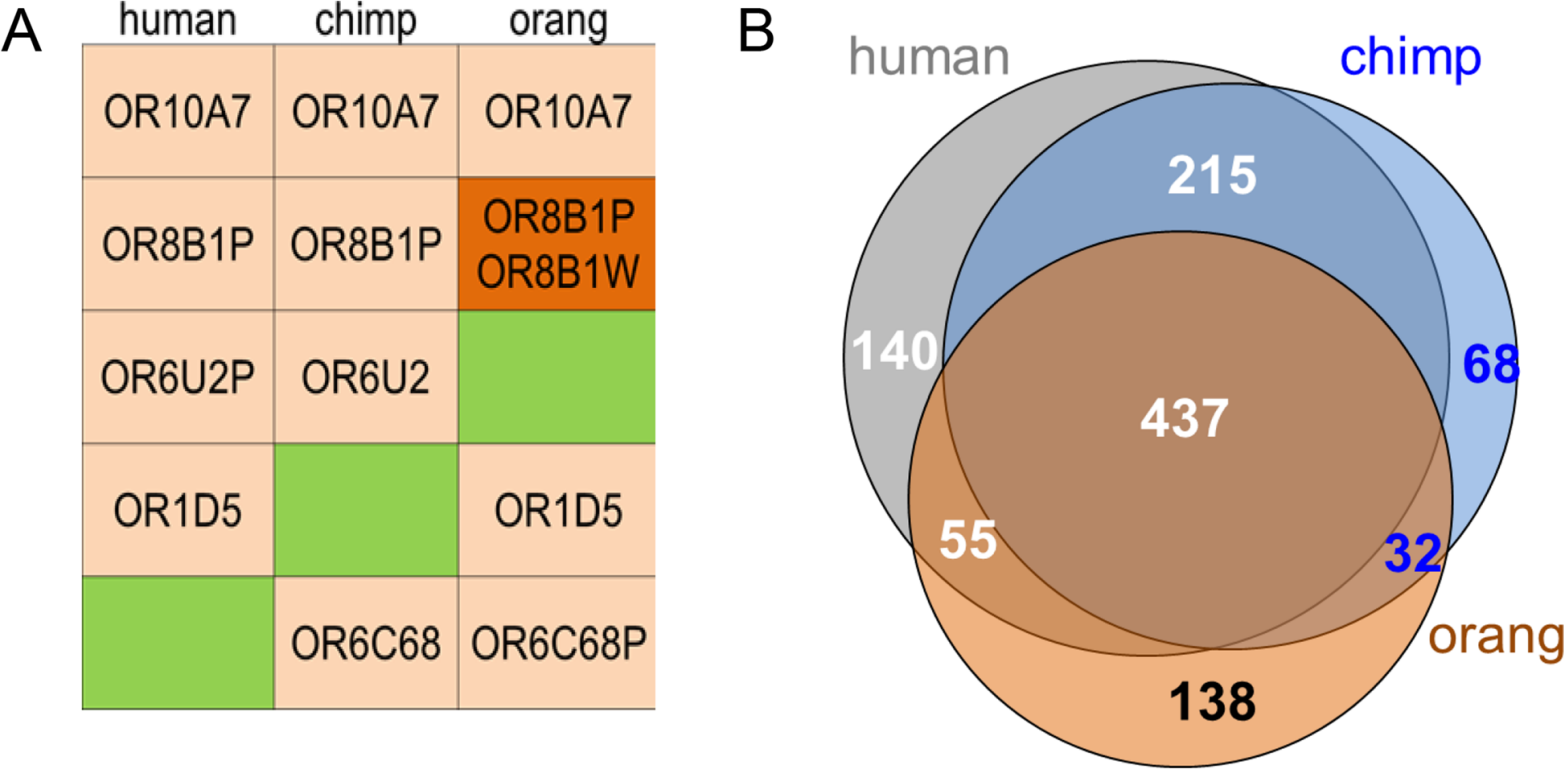
A three-way comparison of human, chimpanzee and orangutan OR symbols. **a**. Examples of how one can use the nomenclature to compare the primate OR repertoires. Symbols that are found in all three primates are in orange boxes, missing symbols are in green boxes (putative deletions), and symbols that are duplicated in one of the species are in dark orange (putative duplications). The “*P*” suffix stands for pseudogene. The “*W*” suffix in the *OR8B1W* symbol indicates that this is one of many orthologs of *OR8B1*. Additional *OR8B1* orthologs with suffixes *B-V* in their symbols were identified in non-primate species. **b**. A Venn diagram summarizing the comparison result. Numbers in white refer to OR gene count in human

We further used the assigned OR symbols of the human, chimpanzee and orangutan to perform a three-way repertoire comparison (Fig. 4). This analysis identified 437 symbols (51.1% of the human ORs) that are shared among all 3 apes, with a significantly higher presentation of class I (“fishlike”, OR families 51–56) OR genes (*p* = 4e-6, chi-square); 215 (29.6%) symbols are shared only between human and chimpanzee, and 55 symbols are shared only between human and orangutan (Fig. 4). Thus, the use of our nomenclature shows that despite the similarity in the OR repertoire size, and in pseudogene content within primates, the gene content is different, in line with previous published findings [32].

### Classification of zebrafish ORs

In an effort to extend this work to non-mammalian species, we classified the OR repertoire of *Danio rerio* (zebrafish), a popular model organism in studies of the olfactory system [41–43]. Previous publications [24, 25] identified a repertoire of ~ 140 OR genes in the zebrafish genome which, although smaller than that of mammalian OR repertoires, shows greater intra-species sequence diversity than in mammals. As mentioned previously, the study of Alioto [24] also proposed a nomenclature for zebrafish ORs which is based on phylogenetic classification and groups the zebrafish ORs into classes and not into families and subfamilies.

**Fig. 5:**
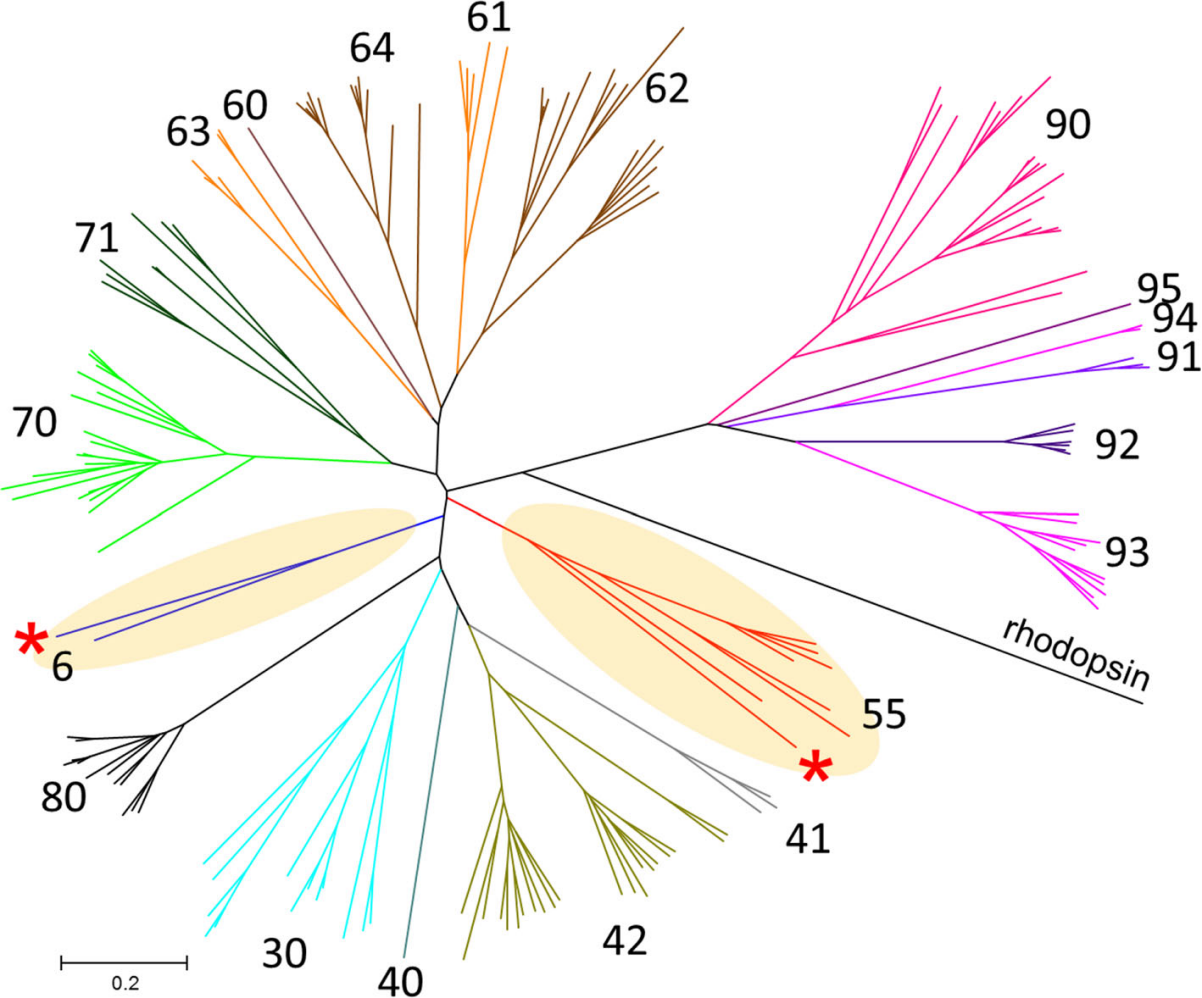
Phylogenetic analysis of zebrafish ORs. A neighbor-joining tree of all zebrafish OR genes and pseudogenes with up to 2 frame-disruptions, mouse OR representatives from families 55 and 6 (the mouse *Or55b3* and *Or6c75,* indicated by red asterisks) and the bovine rhodopsin sequence as an outgroup. The tree is color-coded by the OR family classification where the OR family numbers are indicated next to the corresponding evolutionary branch (see Additional file 3: Table S2 for the relationship between family numbers and classes). All OR families’ main branches are supported by bootstrap values > 90, except for OR family 60 and 40 as their location within the class clade was less certain (bootstrap values of 31 and 77, respectively). The 2 OR families that are shared with mammals are highlighted in yellow

We used version GRCz10 of the zebrafish genome to identify an updated repertoire of 172 zebrafish OR genes, including 13 pseudogenes. We then used the MMS algorithm to assign symbols to each of the zebrafish genes. However, as zebrafish ORs are very distant from mammalian ORs and are expected to be classified into different OR families, we initially manually named zebrafish-specific OR family representatives which were added to the library with the classified ORs (hierarchical clustering, Additional file 3: Fig. S3). Importantly, the OR family numbers were selected to fit with the study of Alioto [24], which used phylogenetic analysis to classify the zebrafish ORs into eight classes, two of which are found in mammals (class A and B). We assigned a distinct set of family numbers for every class (Additional file 3: Table S2), e,g, class C: OR family numbers 30–39, class D: OR family numbers 40–49. Because the mammalian classes have already been assigned the family numbers 51–59 (class A) and 1–14 (class B), classes E, F etc. were assigned numbers starting with 60, 70 and so on, respectively. We then proceeded with the symbol assignment process by applying the MMS algorithm using the same cutoff criteria that we used for mammals (see Methods). The zebrafish OR repertoire was classified into 20 families, of which 2 are shared with mammals (OR families 6 and 55) (Fig. 5). We note that the similarity to mammalian ORs could not be detected using the nomenclature suggested by Alioto [24].

## Discussion

We have consolidated a unified standard nomenclature system for the vertebrate OR superfamily, the largest gene family in the vertebrate genome, and one of the most challenging in terms of orthology relationship identification. We applied the nomenclature to a total of 10,249 genes from the OR repertoires of seven mammals, and zebrafish. The nomenclature is human-centric, a concept that is agreed upon by the vertebrate research community [44], and is based on sequence similarity. OR genes were assigned the same symbols as their putative human orthologs, or otherwise the same symbols as their putative orthologs in other species. If no ortholog was detected the gene was assigned into a relevant subfamily based on the degree of sequence similarity. This was achieved by the development of the MMS algorithm, a systematic classifier for assigning human-based nomenclature to any OR gene by detecting hierarchical similarity relationships between any two species. The algorithm allows more than a single ortholog per human gene, thus reflecting as much as possible the many-to-many homology relationships that characterize the OR family. Although the algorithm was applied to only 7 vertebrate species, it can be applied to other species in a straightforward manner.

As part of this work we established up-to-date OR repertoires for all classified species, using our automated pipeline, which mines OR gene and pseudogene sequences out of complete genomes [11]. Our approach results in a more complete OR repertoire as compared to automated pipelines such as those used by NCBI and Ensembl, because our method is optimized for the olfactory receptors which are unusual in being both single exon and having a high proportion of pseudogenes. Our annotation results are in good agreement with published OR repertoires that were identified in other studies ([24, 25, 31]). Automated orthology prediction pipelines are also not optimized for large complex gene families such as the olfactory receptors as they do not take into account pseudogenes.

The many-to-many relationships among the ORs, which stem from their dynamic evolution, make orthology predictions difficult. Nevertheless, our nomenclature facilitates easy identification of orthology and paralogy relationships within the OR superfamily, which is not possible using the current nomenclature systems across vertebrates. Moreover, our nomenclature assignment within subfamilies is very well supported by the phylogenetic analyses that we conducted. Disagreement between MMS and the phylogenetic analysis was found in only 3.9% of the 4946 ORs that were analyzed (Additional file 6). This rate is highly species specific, as the vast majority of manually renamed ORs were found in horse and cow, while other species had only a very small number of changes. Some of the corrections required in horse and cow are due to lineage specific expansions within these species, where some of the expanded ORs have diverged enough from their ancestral gene to fail to meet the MMS sequence identity cutoff. Future developments in MMS will aim to better accommodate such situations. We note that even for those cases, where the orthology relationship was missed by MMS, the initial classification was to the same OR subfamily, thus the phylogenetic relationship could still be inferred from the symbol, something that is not possible using the current nomenclature systems.

The purpose of our phylogenetic analyses was primarily to demonstrate that our algorithm provides results that are largely consistent with phylogenetic methods. There are several reasons that a purely phylogenetic approach is not ideal for the olfactory receptor superfamily. Firstly, the olfactory receptor gene family contains a high fraction of pseudogenes, which are often not aligned accurately. Secondly, relationships at deeper nodes of the olfactory receptor tree are often not well resolved by phylogenetic methods, due to consistently low bootstrap/confidence values [34–37]. This lack of resolution and low reproducibility leads to difficulty in determining exact evolutionary relationships. Thirdly, phylogenetic methods use large amounts of computational time and memory that rapidly increase with addition of more sequences. As these analyses would need to be repeated every time a new species’ OR repertoire was identified or changed (for example, with a new genome assembly), using phylogenetic methods for nomenclature assignment for olfactory receptors would be hugely time consuming. In contrast, our algorithm is rapid, highly reproducible, and can process coding genes and pseudogenes alike.

Moving forward, we plan to make this method available for general use and hope it will be helpful for other researchers working on their species of interest. It is important to note that no automated method can provide 100% confidence in orthology assignment. It is standard practice within all gene nomenclature committees to conduct manual review and correction of individual gene nomenclature before approval (e.g. [45–47]). We consider the number of genes renamed in this study to be a small proportion of the whole, and also aim to reduce this proportion in future versions of the algorithm.

We have shown that our nomenclature facilitates evolutionary studies of the OR gene families. Species-specific expansions or deletions as well as putative orthologs are identified instantly and clearly by their symbols. The utility of a unified nomenclature system as a tool in evolutionary studies was demonstrated by Hayden [48] and Hughes [49]. These studies applied the human OR family classification method to OR repertoires coming from diverse mammalian taxa to identify specific expansions important for each habitat. The human OR family classification was used also by Vandewege [50] to study the OR gene repertoire in several sauropsid genomes. Their results indicated a highly divergent composition of OR repertoires, which derives from lineage-specific combinations of gene expansions, losses, and retentions of ancestral OR genes. Nevertheless, those studies utilized only the family classification, and not the classification into subfamilies. Here, we have shown that the subfamily classification provides a simple way to identify large deletions (e.g. whole OR clusters), as well as species-specific expansions, at a higher resolution than using only the family classification. By comparing the symbols of human ORs to those of the chimpanzee we obtained the same results as Go [32] who reported ~ 650 human-chimpanzee orthologs. Interestingly, that study identified ~ 420 OR orthologs among human, chimpanzee, and macaque, comparable to the number of putative orthologs that we found among human, chimpanzee and orangutan. The high representation of Class I OR genes in this group also fits well with a previous study, which found a higher proportion of intact genes among Class I ORs relative to Class II ORs in humans [12], which might suggest that a high proportion of Class I ORs are functional.

Finally, as a proof of concept, we extended our nomenclature system to a non-mammalian animal, the zebrafish. A previous comparative study of fish and tetrapods [25] suggested that the most recent common ancestor between jawed and jawless vertebrates had at least 2 OR genes, which evolved to nine in the common ancestor of fishes and tetrapods. Eight of these expanded in the fish genome. Only 2 are present in the mammalian genome, Class I for families 52–56, and Class II for families 1–14, where Class II has expanded tremendously. Our nomenclature assigns a distinct set of family numbers to every class of OR genes (Additional file 3: Table S2) and thus is in agreement with the current understanding of the evolution of vertebrate ORs [24, 25]. The clear advantage of our novel nomenclature is that it allows instant identification of the ORs that are closest to mammalian ORs. While zebrafish ORs are highly diverged, we believe that the MMS algorithm can be applied successfully to other vertebrates such as reptiles and birds. Our previous analyses of zebrafinch [51], as well as other studies of ORs from various avian and reptilian species [37, 50, 52, 53] clearly show that these ORs group well with the mammalian OR classes and families. This is consistent with our preliminary classification of the *Anolis carolinensis* (lizard) OR repertoire that we have recently performed (data not shown). As we apply this method to more distantly related vertebrates, we will continue to critically assess the suitability of the similarity cutoffs. The classification of the zebrafish ORs is included here to demonstrate the utility of a unified nomenclature and as a guideline for classification of ORs from other fishes. The clear advantage of our novel nomenclature is that it allows instant identification of the ORs that are closest to mammalian ORs.

## Conclusions

We developed a unified and standardized nomenclature system for ORs that can be easily applied across all vertebrates. The nomenclature allows for easy and immediate cross-species comparisons and provides a powerful framework for evolutionary studies. Implementation of the proposed nomenclature by the VGNC is currently in progress.

## Methods

### OR gene sequences

We used the HORDE pipeline [11] to establish the OR repertoires of the species described in this work. The pipeline mines OR gene and pseudogene sequences out of any given genome to obtain the complete OR repertoire of that species, and is based on a series of TBLASTN searches as explained in detail in Olender [11]. Briefly, we use a set of ~ 500 ORs (“OR baits”), chosen from different OR subfamilies, and different vertebrates species, in a TBLASTN search to discover putative genomic OR loci, suspected to harbor OR genes. Sequences of putative OR loci are translated with bioperl to yield an ORFs of 280–350 amino acids (for intact genes), or using FASTY [54] which is capable of generating a “corrected” OR sequence by the introduction of frameshifts into the sequence (for pseudogenes). The resulting “OR protein candidates” are classified as ORs or non-ORs by BLASTP comparison against HORDE OR sequences. OR candidates with at least 40% sequence identity over at least 100 amino acids to another OR are considered as OR genes. The OR repertoires (Table 1) were compared to relevant publications and public resources including Mouse Genome Informatics (MGI, [55]), Rat Genome Database (RGD, [56]), The Zebrafish Information Network (ZFIN, [57]), as well as Ensembl [58] and NCBI [59]. The sizes of the identified repertoires are in good agreement with those resources, as well as with published studies [24, 25, 31]. See Additional file 1, and Additional file 8 for more details and protein sequences.

### MMS algorithm

MMS is based on a comparison of a given OR repertoire to a sequence library with classified ORs. The algorithm, encoded by a serial of Perl scripts, is composed of 1. Preparation of input libraries; 2. Calculation of the all-vs-all similarity matrix; 3. Symbol assignment. In step 1 the algorithm prepares the required libraries and other relevant parameters (length, symbols etc.). The algorithm uses 2 libraries, one with human ORs and the second with non-human classified ORs. While the human OR library is constant, the second library grew progressively as more species were included, such that mouse (the first classified species) was compared to human (and available sequences from dog [18] and opossum [5]), rat was compared to mouse and human and so on. After running BLASTP, MMS determines the best hit for every OR gene from human and non-human ORs and vice-versa. In addition, the best hit within the given OR repertoire is determined (required in step 4, Fig. 1). The best hit is calculated as the hit with the highest % identity, where the % identity is defined as the number of identical amino-acids divided by the mean gene length in the pairwise comparison. In a second round of MMS, the % identity was defined as the number of identical amino-acids divided by the length of the shorter gene in each pairwise comparison. This was done to ensure the reliable classification of OR pseudogenes which tend to have a shorter conceptual translation, and thus some pseudogenes might not be classified correctly when mean length is used in the % identity calculation. Finally, symbols are assigned as explained in Fig. 1 and above.

### Synteny tests

To test if potential orthologous genes are syntenic to a human gene we used the on-line UCSC liftOver tool (https://genome.ucsc.edu/cgi-bin/hgLiftOver), which converts genome coordinates between assemblies based on alignments of the human genome (GRCh38) to other genomes. The genome assemblies we used are given in Table 1. We used the default parameters and allowed multiple output regions. Where multiple output regions were returned, the region closest to the human OR ortholog was selected for synteny analysis.

### Assigning symbols to zebrafish ORs

We used hierarchical clustering to select representatives from each OR subfamily (Additional file 3: Fig. S3). This was done by creating a multiple alignment of the full zebrafish OR repertoire using clustalx2 [60] followed by calculation of the sequence identity matrix using BioEdit [61]. We then applied hierarchical clustering to the sequence identity matrix using Matlab2016a with “unweighted average distance” and “correlation” parameters in the hierarchical tree creation, and a cutoff of 0.48 for the ‘distance’ criterion in the clustering. This yielded a crude estimation of the zebrafish OR family structure, which allowed a random selection of OR family representatives. MMS was then applied, where the zebrafish representatives previously selected were included in the non-human OR sequence library. This was sufficient to classify 80% of the zebrafish OR genes. The process was completed by selecting additional OR family representatives using a phylogenetic tree (Fig. 5) and repetition of the MMS process.

### Phylogenetic analysis

Predicted coding sequences of the members of the 50 largest mammalian OR subfamilies (by the total number of ORs in human, chimpanzee, orangutan, horse, cattle, dog, mouse and rat) were used for phylogenetic analysis. For each subfamily, alignments were generated using ClustalX 2.1 [60] including the predicted conceptual translation sequence of all intact OR genes, as well as pseudogenes with no more than 2 frame-disruptions. Alignments were trimmed using trimAl v1.4 [62] to remove positions with more than 80% gaps. The Maximum Likelihood trees were reconstructed using the IQTree webserver [63], using the “AUTO” amino acid substitution model, and the ultra-fast bootstrap option (UFBoot) [64] with 1000 samples. Maximum Likelihood consensus trees are displayed with ultrafast bootstrap values on the branch nodes (Additional file 4). The Bayesian trees were inferred using MrBayes v3.2 [65], with amino acid model prior set to mixed, number of generations = 1,000,000, and burn-in set to 25%. FigTree v1.4.3 [66] was used for tree visualization. Bayesian trees are displayed with posterior probabilities on the branch nodes (Fig. 2a, Additional file 5).

For phylogenetic analysis of zebrafish ORs, a neighbor-joining tree was constructed with MEGA7 [67] using the p-distance method for estimating the evolutionary distances in units of the number of amino acid differences per site.

### Analysis of OGGs classification

To compare the classification of Niimura [31] to HORDE’s subfamily classification, we downloaded the supplementary material of Niimura [31], and extracted the OR protein sequences of the species in our study (cow, mouse and horse). We then used BLASTP to match these sequences with ours, using a cutoff of > 97% sequence identity over at least 80% of the protein sequence. This analysis was required since the study of Niimura [31] uses internal identifiers for the OR sequences without giving genomic coordinates, and because of differences in the genome versions (for mouse and cow) that might result in sequence differences. We successfully matched 4587 OR genes and pseudogenes that were classified to 737 OGGs and to 285 OR subfamilies. An additional 123 ORs, mostly pseudogenes, were classified using our method into distinct 101 distinct subfamilies.

## Supplementary information

**Additional file 1.** The symbols, genomic locations and aliases of the OR repertoires in this work.

**Additional file 2.** Summary of the OR genes in subfamilies.

**Additional file 3:Figure S1.** The percentage of ORs assigned with human symbols. Dark blue = mutual best hits; light blue = second best hits. **Figure S2.** Percentage of pseudogenes in ORs with orthologous symbols (blue), versus ORs with unique symbols (light blue). Orthologous symbol; symbol that is shared between at least two mammals (including human second best hits). Unique symbol; symbol that appears in one mammal. Numbers are Fisher exact *p* values. **Figure S3.** Hierarchical clustering of the zebrafish sequence identity matrix. **Table S2.** The zebrafish OR family numbers.

**Additional file 4.** Phylogenetic analysis of the 50 largest OR subfamilies using Maximum Likelihood. Maximum Likelihood phylogenies were generated for the 50 largest OR subfamilies as described in the Methods. Maximum likelihood consensus trees are displayed with ultrafast bootstrap values on the branch nodes. ORs that were renamed manually are annotated with an asterisk (*). Scale bar represents the number of amino acid changes per site.

**Additional file 5.** Phylogenetic analysis of the 50 largest OR subfamilies using Bayesian inference. Bayesian phylogenies were generated for the 50 largest OR subfamilies as described in the Methods. Bayesian trees are displayed with posterior probabilities on the branch nodes. ORs that were renamed manually are annotated with an asterisk (*). Scale bar represents the number of amino acid changes per site.

**Additional file 6.** A list of 195 ORs that were renamed manually.

**Additional file 7:Table S1.** Species-specific expansion/deletions. A. OR subfamilies with species-specific expansions. An expansion is defined as a subfamily for which one of the studied organisms has ≥2 times the number of ORs than all the other organisms. Subfamilies with a single OR were omitted from the analysis. The green background indicates expansion. B. Putative large genomic deletions. A putative deletion is defined as an OR subfamily that is missing in primates, Laurasiatheria, or rodents. The numbers are the gene count per subfamily. The genomic locations of subfamilies that are encoded from a single genomic cluster are shown in the right side of the table.

**Additional file 8.** Protein sequences of the OR genes and pseudogenes in this work.

## Data Availability

All data generated in this study are included in this published article, its supplementary information files, and in the Human Olfactory Receptor Data Explorer database (HORDE, https://genome.weizmann.ac.il/horde/).

## Abbreviations

OR: Olfactory receptor
MMS: Mutual Maximum Similarity
VGNC: Vertebrate Gene Nomenclature Committee
