# Supplementary material for: A unified nomenclature for vertebrate olfactory receptors
Source: BMC Evol Biol. 2020 Apr 15;20:42. doi: 10.1186/s12862-020-01607-6 (PMC7160942; doi:10.1186/s12862-020-01607-6)
Supplement: Supplementary file 3 — Additional file 3:Figure S1. The percentage of ORs assigned with human symbols. Dark blue = mutual best hits; light blue = second best hits. Figure S2. Percentage of pseudogenes in ORs with orthologous symbols (blue), versus ORs with unique symbols (light blue). Orthologous symbol; symbol that is shared between at least two mammals (including human second best hits). Unique symbol; symbol that appears in one mammal. Numbers are Fisher exact p values. Figure S3. Hierarchical clustering of the zebrafish sequence identity matrix. Table S2. The zebrafish OR family numbers. [file 12862_2020_1607_MOESM3_ESM.pptx]

## Slide 1
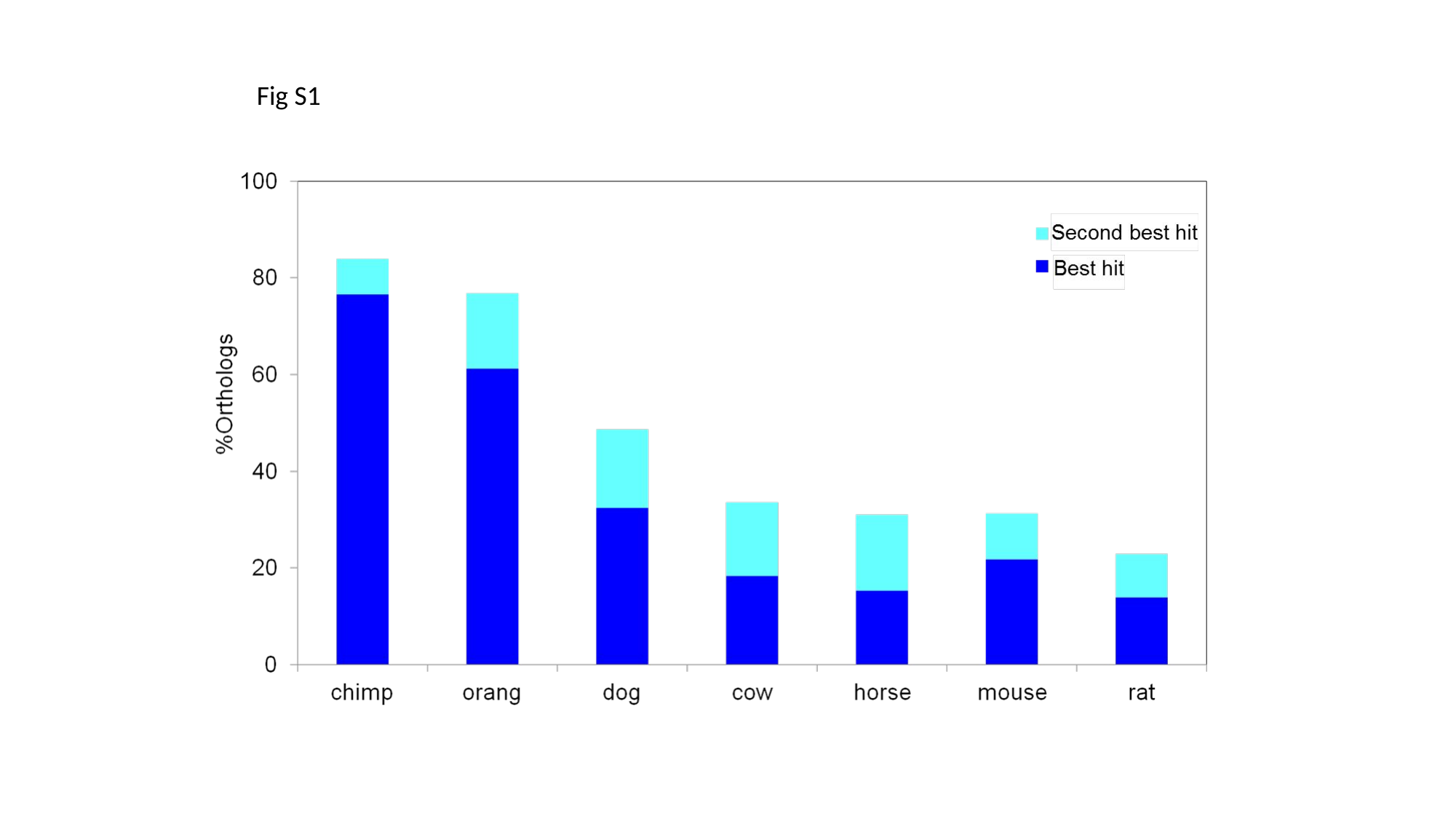

Fig S1

## Slide 2
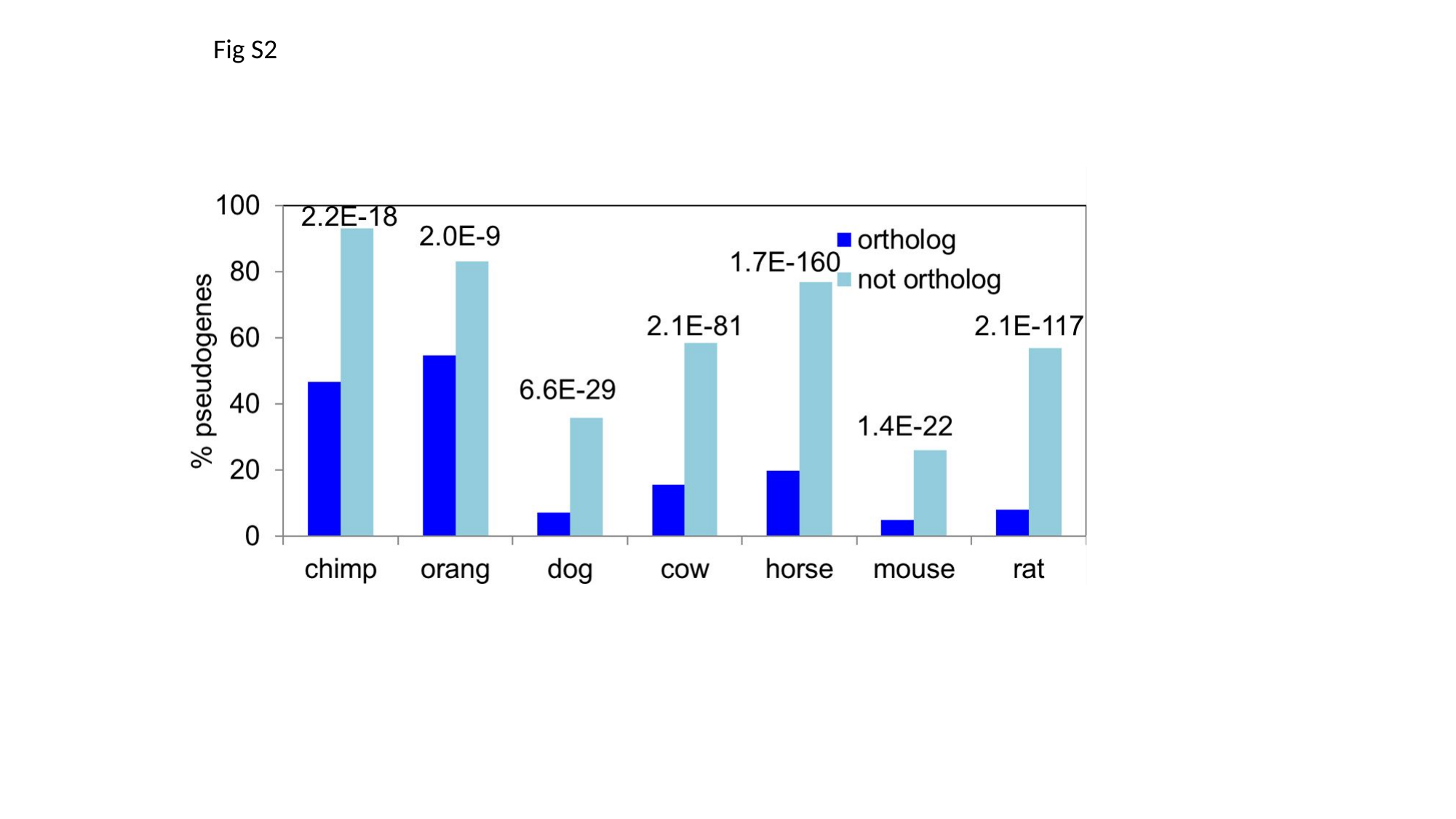

Fig S2

## Slide 3
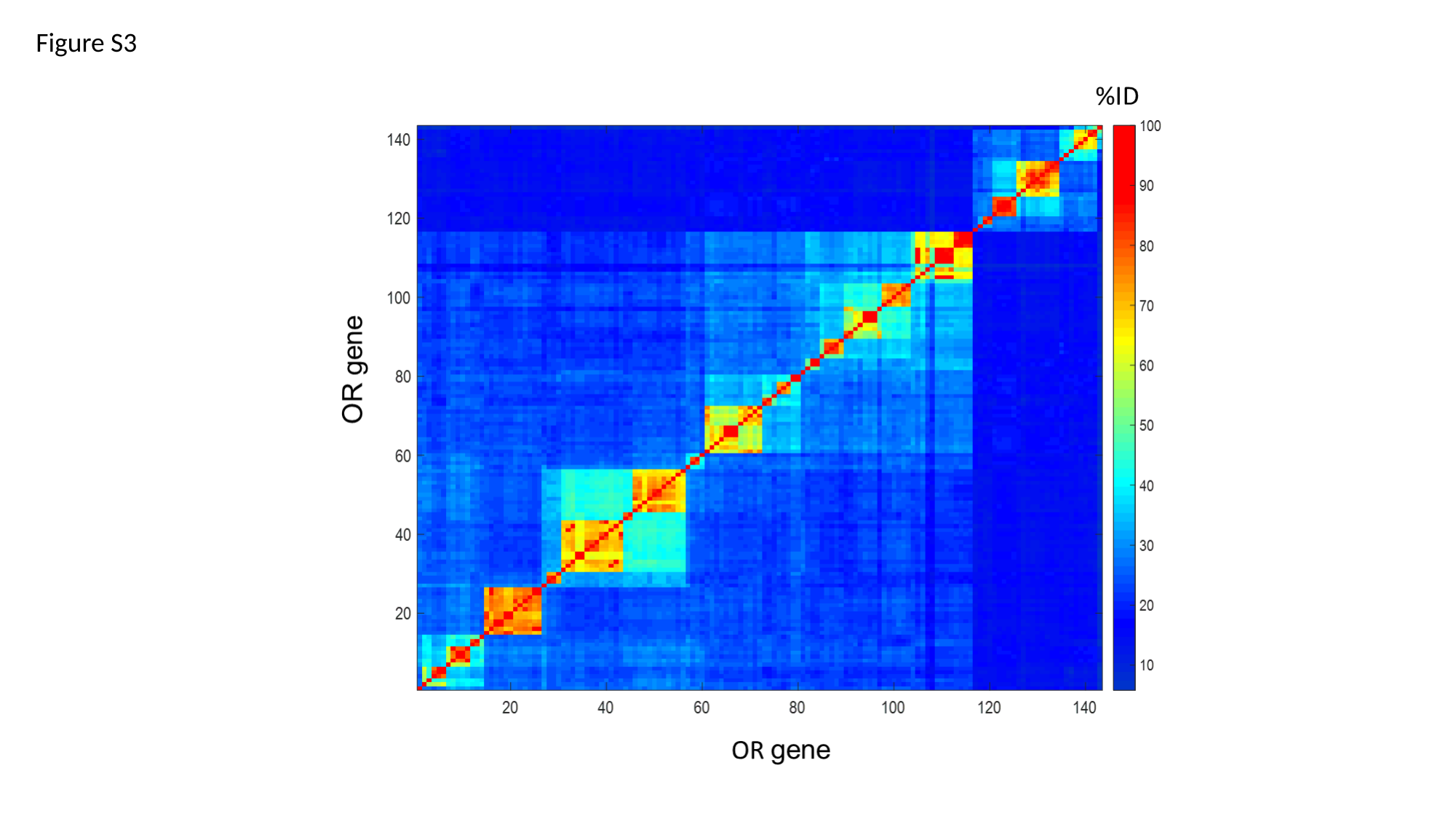

Figure S3

## Slide 4
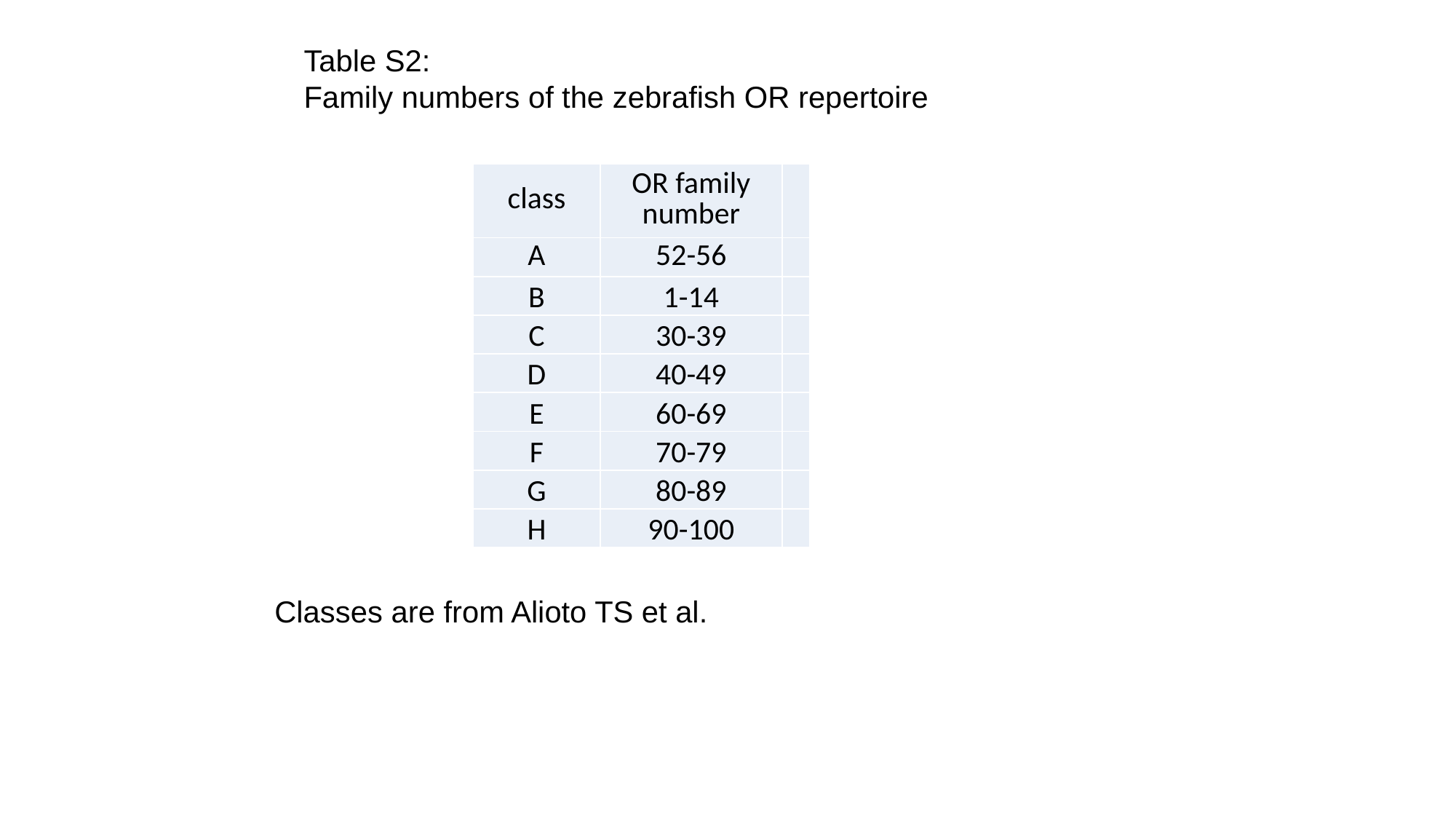

Table S2:
Family numbers of the zebrafish OR repertoire
| class | OR family number | |
| --- | --- | --- |
| A | 52-56 | |
| B | 1-14 | |
| C | 30-39 | |
| D | 40-49 | |
| E | 60-69 | |
| F | 70-79 | |
| G | 80-89 | |
| H | 90-100 | |
Classes are from Alioto TS et al.
