# Supplementary material for: A unified nomenclature for vertebrate olfactory receptors
Source: BMC Evol Biol. 2020 Apr 15;20:42. doi: 10.1186/s12862-020-01607-6 (PMC7160942; doi:10.1186/s12862-020-01607-6)
Supplement: Supplementary file 5 — Additional file 5. Phylogenetic analysis of the 50 largest OR subfamilies using Bayesian inference. Bayesian phylogenies were generated for the 50 largest OR subfamilies as described in the Methods. Bayesian trees are displayed with posterior probabilities on the branch nodes. ORs that were renamed manually are annotated with an asterisk (*). Scale bar represents the number of amino acid changes per site. [file 12862_2020_1607_MOESM5_ESM.pdf]

Additional File 5 – Bayesian phylogenetic analysis of the 50 largest subfamilies.

Bayesian phylogenies were generated for the 50 largest OR subfamilies. For each subfamily, Bayesian phylogenetic analysis was performed on the conceptual translation of all members of the subfamily from the mammalian OR repertoires classified in this study (OR pseudogenes with more than two frame disruptions were excluded). Human OR51E1 was used as the outgroup for Class II OR subfamilies, and human OR1E2 was used as the outgroup for Class I OR subfamilies. Alignments were trimmed as described in the methods. Trees were inferred using MrBayes v3.2 {Ronquist, 2012 #65}, with amino acid model prior set to mixed, number of generations = 1,000,000, and burn-in set to 25%. Bayesian trees are displayed with posterior probabilities on the branch nodes. ORs that were renamed manually are annotated with an asterisk (*). Scale bar represents the number of amino acid changes per site.

Table of Contents

Page	*OR subfamily*
3	OR1E
4	OR1F
5	OR1J
6	OR1L
7	OR2A
8	OR2B
9	OR2L
10	OR2T
11	OR2W
12	OR4A
13	OR4C
14	OR4D
15	OR4F
16	OR4K
17	OR4P
18	OR5AN
19	OR5B
20	OR5D
21	OR5H
22	OR5M
23	OR5P
24	OR5T
25	OR5W
26	OR6C
27	OR7A
28	OR7G
29	OR8B
30	OR8C
31	OR8G

Page	*OR subfamily*
32	OR8K
33	OR8S
34	OR9G
35	OR9S
36	OR10AG
37	OR10AL
38	OR10A
39	OR10D
40	OR10G
41	OR10H
42	OR10J
43	OR11H
44	OR13C
45	OR51A
46	OR51F
47	OR52A
48	OR52B
49	OR52E
50	OR52N
51	OR56A
52	OR56B

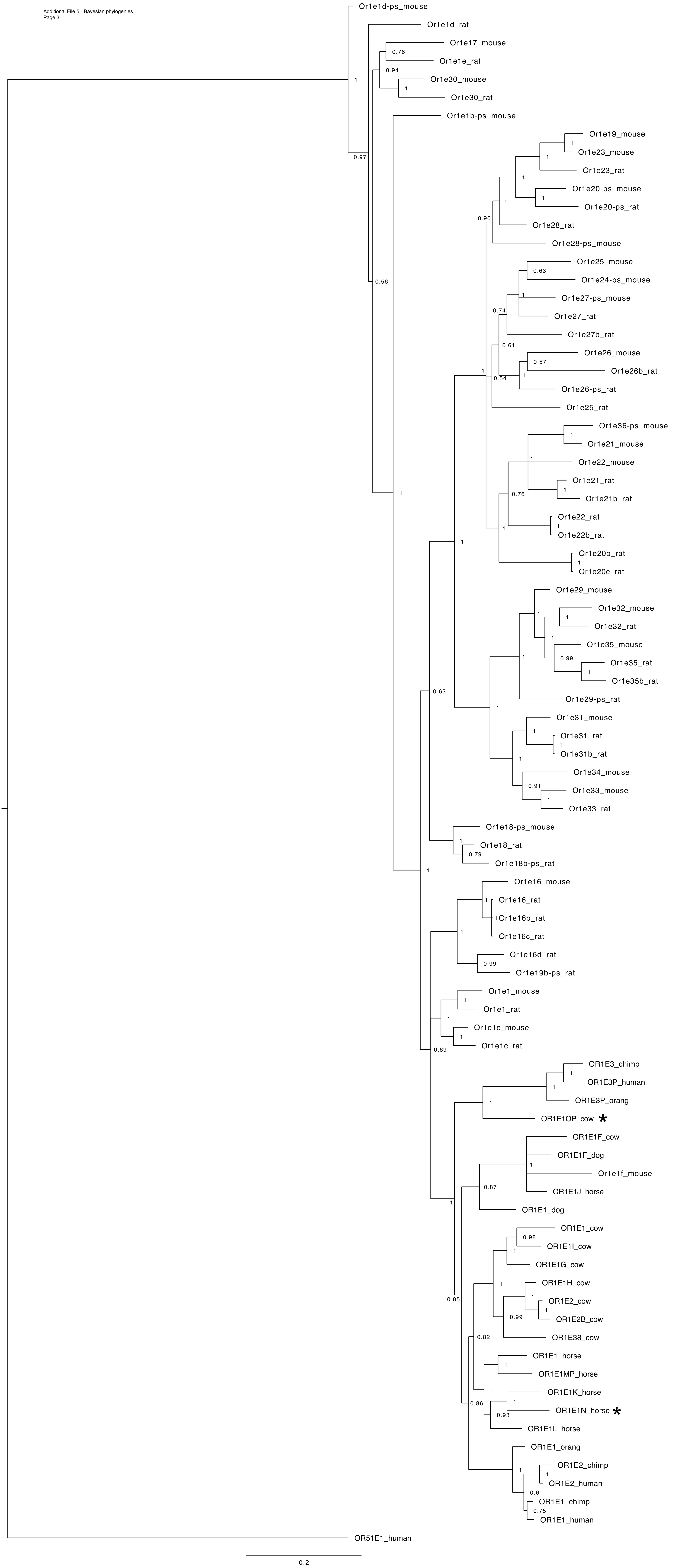
Or1e1d-ps_mouse
Or1e1d_rat
Or1e17_mouse
Or1e1e_rat
Or1e30_mouse
Or1e30_rat
Or1e1b-ps_mouse
Or1e19_mouse
Or1e23_mouse
Or1e23_rat
Or1e20-ps_mouse
Or1e20-ps_rat
Or1e28_rat
Or1e28-ps_mouse
Or1e25_mouse
Or1e24-ps_mouse
Or1e27-ps_mouse
Or1e27_rat
Or1e27b_rat
Or1e26_mouse
Or1e26b_rat
Or1e26-ps_rat
Or1e25_rat
Or1e36-ps_mouse
Or1e21_mouse
Or1e22_mouse
Or1e21_rat
Or1e21b_rat
Or1e22_rat
Or1e22b_rat
Or1e20b_rat
Or1e20c_rat
Or1e29_mouse
Or1e32_mouse
Or1e32_rat
Or1e35_mouse
Or1e35_rat
Or1e35b_rat
Or1e29-ps_rat
Or1e31_mouse
Or1e31_rat
Or1e31b_rat
Or1e34_mouse
Or1e33_mouse
Or1e33_rat
Or1e18-ps_mouse
Or1e18_rat
Or1e18b-ps_rat
Or1e16_mouse
Or1e16_rat
Or1e16b_rat
Or1e16c_rat
Or1e16d_rat
Or1e19b-ps_rat
Or1e1_mouse
Or1e1_rat
Or1e1c_mouse
Or1e1c_rat
OR1E3_chimp
OR1E3P_human
OR1E3P_orang
OR1E1OP_cow *
OR1E1F_cow
OR1E1F_dog
Or1e1f_mouse
OR1E1J_horse
OR1E1_dog
OR1E1_cow
OR1E1I_cow
OR1E1G_cow
OR1E1H_cow
OR1E2_cow
OR1E2B_cow
OR1E38_cow
OR1E1_horse
OR1E1MP_horse
OR1E1K_horse
OR1E1N_horse *
OR1E1L_horse
OR1E1_orang
OR1E2_chimp
OR1E2_human
OR1E1_chimp
OR1E1_human
OR51E1_human
0.2

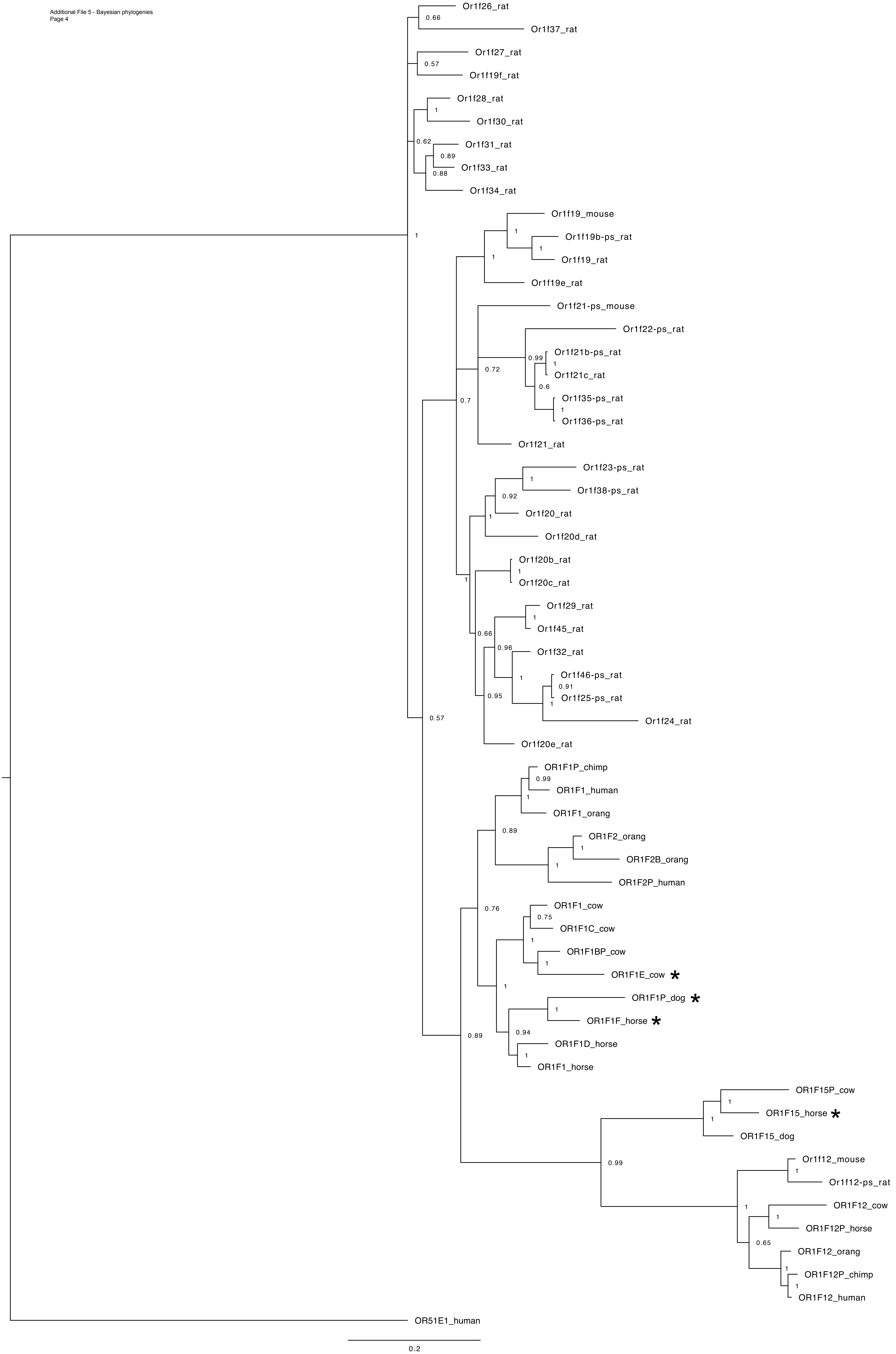
Or1f26_rat
Or1f37_rat
Or1f27_rat
Or1f19f_rat
Or1f28_rat
Or1f30_rat
Or1f31_rat
Or1f33_rat
Or1f34_rat
Or1f19_mouse
Or1f19b-ps_rat
Or1f19_rat
Or1f19e_rat
Or1f21-ps_mouse
Or1f22-ps_rat
Or1f21b-ps_rat
Or1f21c_rat
Or1f35-ps_rat
Or1f36-ps_rat
Or1f21_rat
Or1f23-ps_rat
Or1f38-ps_rat
Or1f20_rat
Or1f20d_rat
Or1f20b_rat
Or1f20c_rat
Or1f29_rat
Or1f45_rat
Or1f32_rat
Or1f46-ps_rat
Or1f25-ps_rat
Or1f24_rat
Or1f20e_rat
OR1F1P_chimp
OR1F1_human
OR1F1_orang
OR1F2_orang
OR1F2B_orang
OR1F2P_human
OR1F1_cow
OR1F1C_cow
OR1F1BP_cow
OR1F1E_cow *
OR1F1P_dog *
OR1F1F_horse *
OR1F1D_horse
OR1F1_horse
OR1F15P_cow
OR1F15_horse *
OR1F15_dog
Or1f12_mouse
Or1f12-ps_rat
OR1F12_cow
OR1F12P_horse
OR1F12_orang
OR1F12P_chimp
OR1F12_human
OR51E1_human
0.2

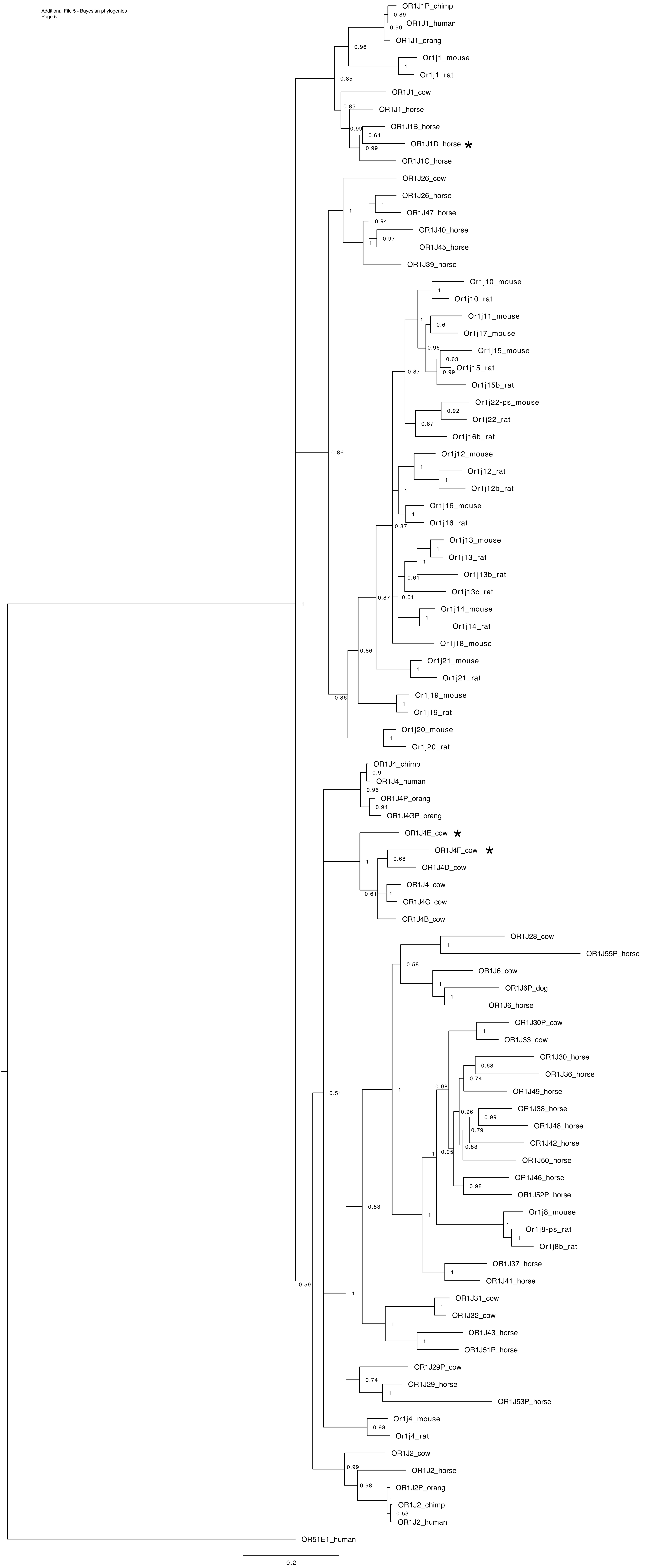
OR1J1P_chimp
OR1J1_human
OR1J1_orang
Or1j1_mouse
Or1j1_rat
OR1J1_cow
OR1J1_horse
OR1J1B_horse
OR1J1D_horse *
OR1J1C_horse
OR1J26_cow
OR1J26_horse
OR1J47_horse
OR1J40_horse
OR1J45_horse
OR1J39_horse
Or1j10_mouse
Or1j10_rat
Or1j11_mouse
Or1j17_mouse
Or1j15_mouse
Or1j15_rat
Or1j15b_rat
Or1j22-ps_mouse
Or1j22_rat
Or1j16b_rat
Or1j12_mouse
Or1j12_rat
Or1j12b_rat
Or1j16_mouse
Or1j16_rat
Or1j13_mouse
Or1j13_rat
Or1j13b_rat
Or1j13c_rat
Or1j14_mouse
Or1j14_rat
Or1j18_mouse
Or1j21_mouse
Or1j21_rat
Or1j19_mouse
Or1j19_rat
Or1j20_mouse
Or1j20_rat
OR1J4_chimp
OR1J4_human
OR1J4P_orang
OR1J4GP_orang
OR1J4E_cow *
OR1J4F_cow *
OR1J4D_cow
OR1J4_cow
OR1J4C_cow
OR1J4B_cow
OR1J28_cow
OR1J55P_horse
OR1J6_cow
OR1J6P_dog
OR1J6_horse
OR1J30P_cow
OR1J33_cow
OR1J30_horse
OR1J36_horse
OR1J49_horse
OR1J38_horse
OR1J48_horse
OR1J42_horse
OR1J50_horse
OR1J46_horse
OR1J52P_horse
Or1j8_mouse
Or1j8-ps_rat
Or1j8b_rat
OR1J37_horse
OR1J41_horse
OR1J31_cow
OR1J32_cow
OR1J43_horse
OR1J51P_horse
OR1J29P_cow
OR1J29_horse
OR1J53P_horse
Or1j4_mouse
Or1j4_rat
OR1J2_cow
OR1J2_horse
OR1J2P_orang
OR1J2_chimp
OR1J2_human
OR51E1_human
0.89
0.99
0.96
0.85
0.85
0.99
0.64
0.99
1
0.94
0.97
0.6
0.96
0.63
0.99
0.87
0.92
0.87
0.86
0.61
0.61
0.86
0.9
0.95
0.94
0.68
0.61
0.58
0.51
0.98
0.74
0.96
0.99
0.79
0.83
0.95
0.98
0.83
0.59
0.74
0.98
0.99
0.98
0.53
0.2

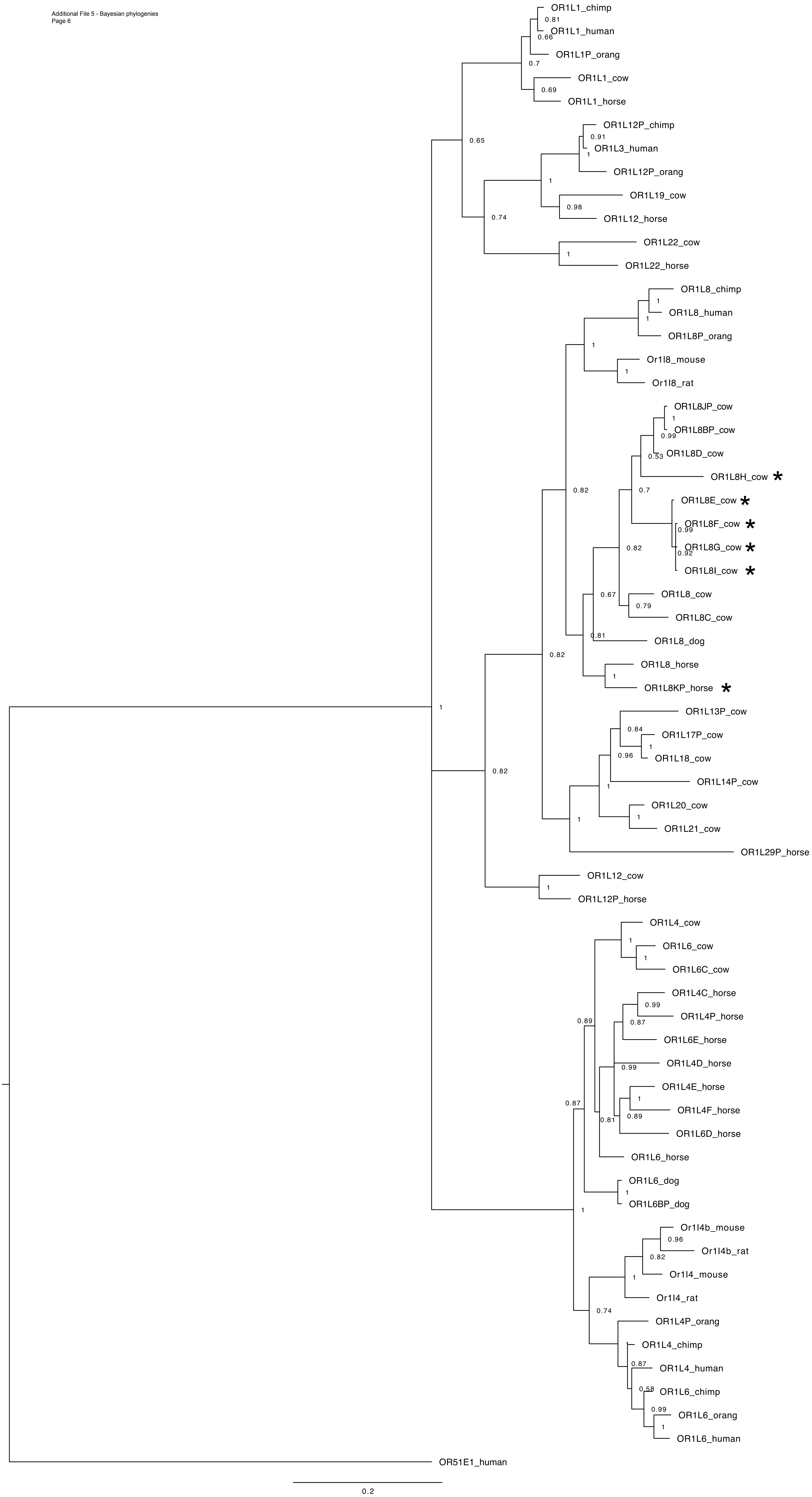
OR1L1_chimp
0.81
OR1L1_human
0.66
OR1L1P_orang
0.7
OR1L1_cow
0.69
OR1L1_horse
0.65
OR1L12P_chimp
0.91
OR1L3_human
1
OR1L12P_orang
1
OR1L19_cow
0.98
OR1L12_horse
0.74
OR1L22_cow
1
OR1L22_horse
OR1L8_chimp
1
OR1L8_human
1
OR1L8P_orang
1
Or1l8_mouse
1
Or1l8_rat
OR1L8JP_cow
1
OR1L8BP_cow
0.99
OR1L8D_cow
0.53
OR1L8H_cow *
0.7
0.82
OR1L8E_cow *
OR1L8F_cow *
0.99
OR1L8G_cow *
0.92
OR1L8I_cow *
0.82
0.67
OR1L8_cow
0.79
OR1L8C_cow
0.81
OR1L8_dog
0.82
OR1L8_horse
1
OR1L8KP_horse *
1
OR1L13P_cow
0.84
OR1L17P_cow
1
0.96
OR1L18_cow
OR1L14P_cow
1
OR1L20_cow
1
OR1L21_cow
1
OR1L29P_horse
0.82
OR1L12_cow
1
OR1L12P_horse
OR1L4_cow
1
OR1L6_cow
1
OR1L6C_cow
OR1L4C_horse
0.99
OR1L4P_horse
0.89
0.87
OR1L6E_horse
OR1L4D_horse
0.99
OR1L4E_horse
1
OR1L4F_horse
0.89
0.87
0.81
OR1L6D_horse
OR1L6_horse
OR1L6_dog
1
OR1L6BP_dog
1
Or1l4b_mouse
0.96
Or1l4b_rat
0.82
Or1l4_mouse
1
Or1l4_rat
0.74
OR1L4P_orang
1
OR1L4_chimp
0.87
OR1L4_human
0.58
OR1L6_chimp
0.99
OR1L6_orang
1
OR1L6_human
OR51E1_human
0.2

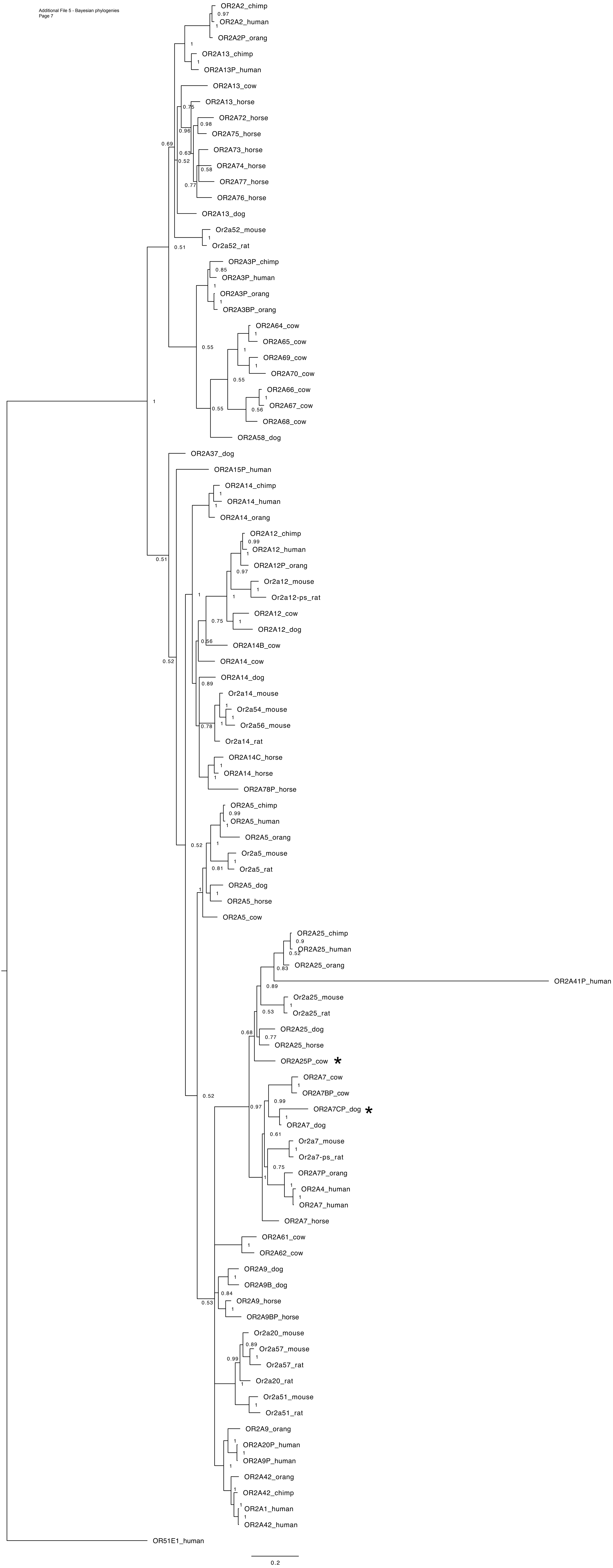
OR2A2_chimp
OR2A2_human
OR2A2P_orang
OR2A13_chimp
OR2A13P_human
OR2A13_cow
OR2A13_horse
OR2A72_horse
OR2A75_horse
OR2A73_horse
OR2A74_horse
OR2A77_horse
OR2A76_horse
OR2A13_dog
Or2a52_mouse
Or2a52_rat
OR2A3P_chimp
OR2A3P_human
OR2A3P_orang
OR2A3BP_orang
OR2A64_cow
OR2A65_cow
OR2A69_cow
OR2A70_cow
OR2A66_cow
OR2A67_cow
OR2A68_cow
OR2A58_dog
OR2A37_dog
OR2A15P_human
OR2A14_chimp
OR2A14_human
OR2A14_orang
OR2A12_chimp
OR2A12_human
OR2A12P_orang
Or2a12_mouse
Or2a12-ps_rat
OR2A12_cow
OR2A12_dog
OR2A14B_cow
OR2A14_cow
OR2A14_dog
Or2a14_mouse
Or2a54_mouse
Or2a56_mouse
Or2a14_rat
OR2A14C_horse
OR2A14_horse
OR2A78P_horse
OR2A5_chimp
OR2A5_human
OR2A5_orang
Or2a5_mouse
Or2a5_rat
OR2A5_dog
OR2A5_horse
OR2A5_cow
OR2A25_chimp
OR2A25_human
OR2A25_orang
OR2A41P_human
Or2a25_mouse
Or2a25_rat
OR2A25_dog
OR2A25_horse
OR2A25P_cow *
OR2A7_cow
OR2A7BP_cow
OR2A7CP_dog *
OR2A7_dog
Or2a7_mouse
Or2a7-ps_rat
OR2A7P_orang
OR2A4_human
OR2A7_human
OR2A7_horse
OR2A61_cow
OR2A62_cow
OR2A9_dog
OR2A9B_dog
OR2A9_horse
OR2A9BP_horse
Or2a20_mouse
Or2a57_mouse
Or2a57_rat
Or2a20_rat
Or2a51_mouse
Or2a51_rat
OR2A9_orang
OR2A20P_human
OR2A9P_human
OR2A42_orang
OR2A42_chimp
OR2A1_human
OR2A42_human
OR51E1_human
0.2

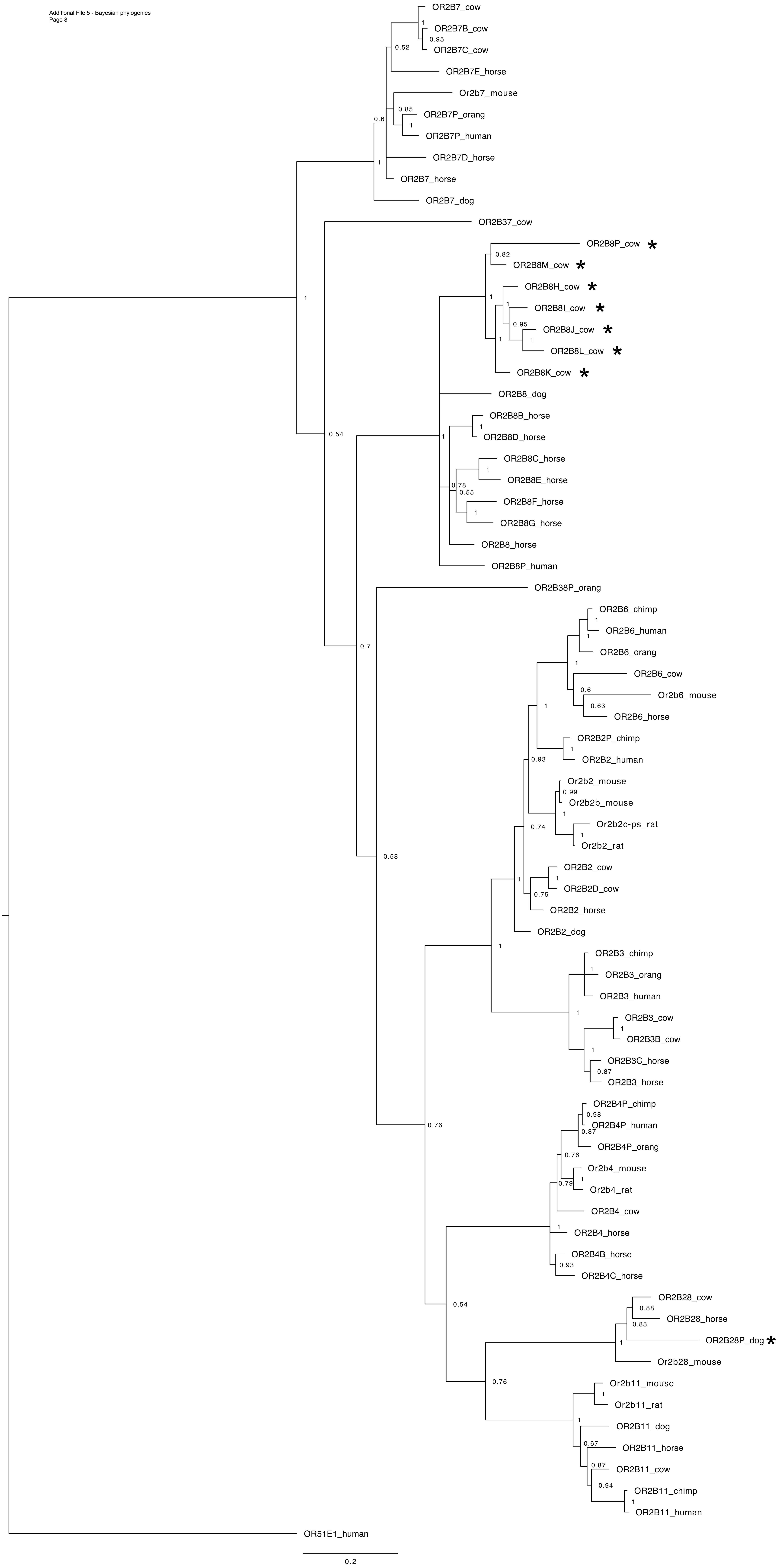
OR2B7_cow
OR2B7B_cow
OR2B7C_cow
OR2B7E_horse
Or2b7_mouse
OR2B7P_orang
OR2B7P_human
OR2B7D_horse
OR2B7_horse
OR2B7_dog
OR2B37_cow
OR2B8P_cow *
OR2B8M_cow *
OR2B8H_cow *
OR2B8I_cow *
OR2B8J_cow *
OR2B8L_cow *
OR2B8K_cow *
OR2B8_dog
OR2B8B_horse
OR2B8D_horse
OR2B8C_horse
OR2B8E_horse
OR2B8F_horse
OR2B8G_horse
OR2B8_horse
OR2B8P_human
OR2B38P_orang
OR2B6_chimp
OR2B6_human
OR2B6_orang
OR2B6_cow
Or2b6_mouse
OR2B6_horse
OR2B2P_chimp
OR2B2_human
Or2b2_mouse
Or2b2b_mouse
Or2b2c-ps_rat
Or2b2_rat
OR2B2_cow
OR2B2D_cow
OR2B2_horse
OR2B2_dog
OR2B3_chimp
OR2B3_orang
OR2B3_human
OR2B3_cow
OR2B3B_cow
OR2B3C_horse
OR2B3_horse
OR2B4P_chimp
OR2B4P_human
OR2B4P_orang
Or2b4_mouse
Or2b4_rat
OR2B4_cow
OR2B4_horse
OR2B4B_horse
OR2B4C_horse
OR2B28_cow
OR2B28_horse
OR2B28P_dog *
Or2b28_mouse
Or2b11_mouse
Or2b11_rat
OR2B11_dog
OR2B11_horse
OR2B11_cow
OR2B11_chimp
OR2B11_human
OR51E1_human
0.2

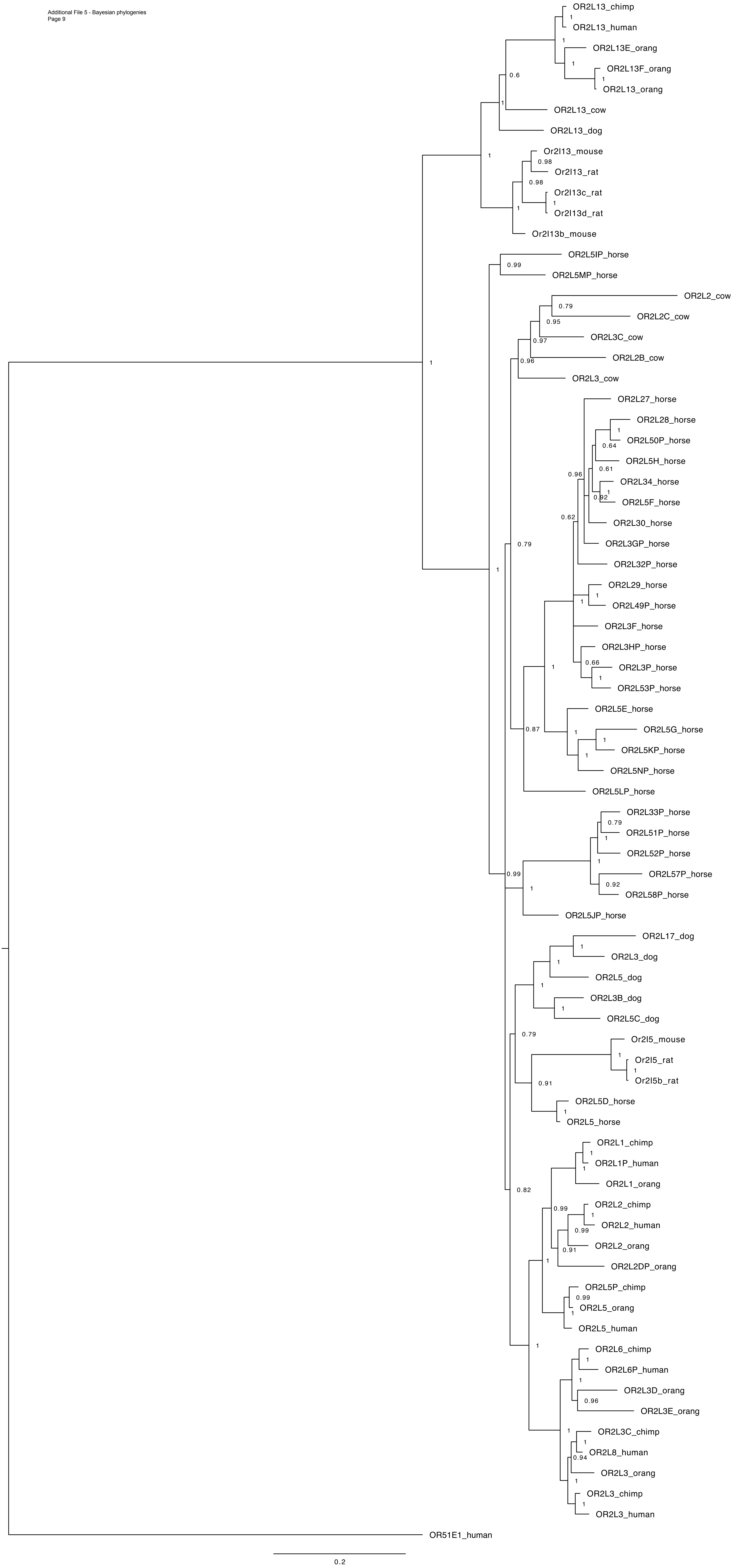
OR2L13_chimp
OR2L13_human
OR2L13E_orang
OR2L13F_orang
OR2L13_orang
OR2L13_cow
OR2L13_dog
Or2l13_mouse
Or2l13_rat
Or2l13c_rat
Or2l13d_rat
Or2l13b_mouse
OR2L5IP_horse
OR2L5MP_horse
OR2L2_cow
OR2L2C_cow
OR2L3C_cow
OR2L2B_cow
OR2L3_cow
OR2L27_horse
OR2L28_horse
OR2L50P_horse
OR2L5H_horse
OR2L34_horse
OR2L5F_horse
OR2L30_horse
OR2L3GP_horse
OR2L32P_horse
OR2L29_horse
OR2L49P_horse
OR2L3F_horse
OR2L3HP_horse
OR2L3P_horse
OR2L53P_horse
OR2L5E_horse
OR2L5G_horse
OR2L5KP_horse
OR2L5NP_horse
OR2L5LP_horse
OR2L33P_horse
OR2L51P_horse
OR2L52P_horse
OR2L57P_horse
OR2L58P_horse
OR2L5JP_horse
OR2L17_dog
OR2L3_dog
OR2L5_dog
OR2L3B_dog
OR2L5C_dog
Or2l5_mouse
Or2l5_rat
Or2l5b_rat
OR2L5D_horse
OR2L5_horse
OR2L1_chimp
OR2L1P_human
OR2L1_orang
OR2L2_chimp
OR2L2_human
OR2L2_orang
OR2L2DP_orang
OR2L5P_chimp
OR2L5_orang
OR2L5_human
OR2L6_chimp
OR2L6P_human
OR2L3D_orang
OR2L3E_orang
OR2L3C_chimp
OR2L8_human
OR2L3_orang
OR2L3_chimp
OR2L3_human
OR51E1_human
0.2

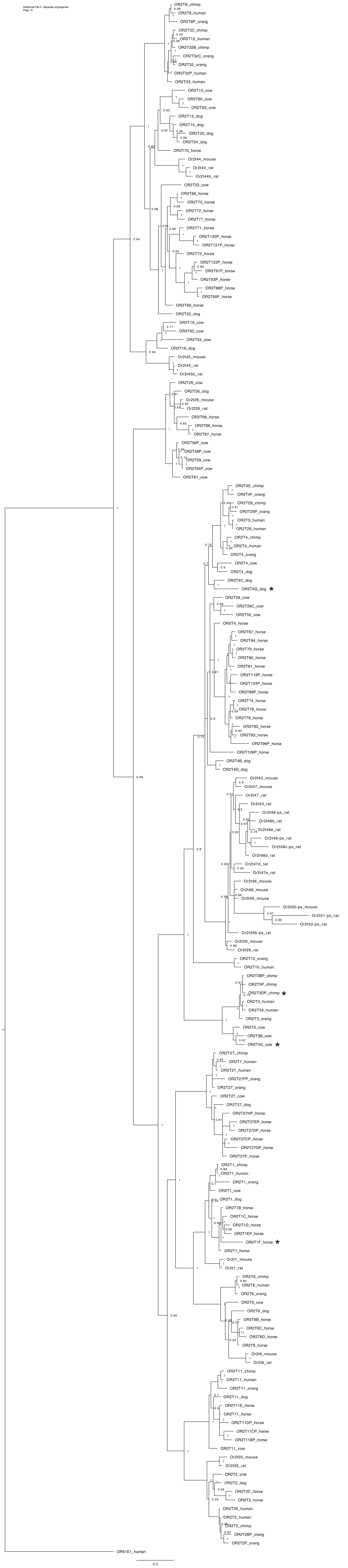

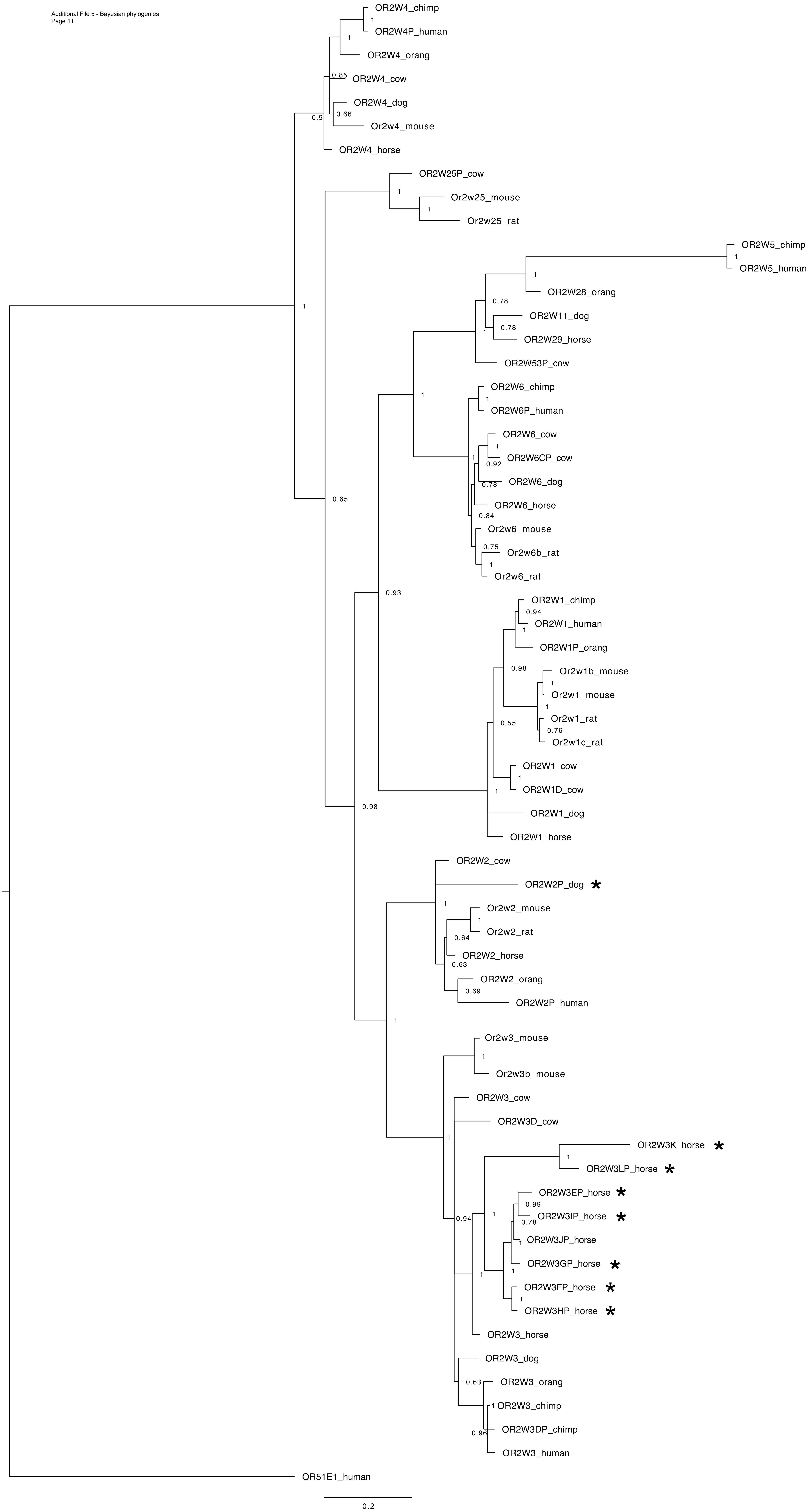
OR2W4_chimp
OR2W4P_human
OR2W4_orang
OR2W4_cow
OR2W4_dog
Or2w4_mouse
OR2W4_horse
OR2W25P_cow
Or2w25_mouse
Or2w25_rat
OR2W5_chimp
OR2W5_human
OR2W28_orang
OR2W11_dog
OR2W29_horse
OR2W53P_cow
OR2W6_chimp
OR2W6P_human
OR2W6_cow
OR2W6CP_cow
OR2W6_dog
OR2W6_horse
Or2w6_mouse
Or2w6b_rat
Or2w6_rat
OR2W1_chimp
OR2W1_human
OR2W1P_orang
Or2w1b_mouse
Or2w1_mouse
Or2w1_rat
Or2w1c_rat
OR2W1_cow
OR2W1D_cow
OR2W1_dog
OR2W1_horse
OR2W2_cow
OR2W2P_dog *
Or2w2_mouse
Or2w2_rat
OR2W2_horse
OR2W2_orang
OR2W2P_human
Or2w3_mouse
Or2w3b_mouse
OR2W3_cow
OR2W3D_cow
OR2W3K_horse *
OR2W3LP_horse *
OR2W3EP_horse *
OR2W3IP_horse *
OR2W3JP_horse
OR2W3GP_horse *
OR2W3FP_horse *
OR2W3HP_horse *
OR2W3_horse
OR2W3_dog
OR2W3_orang
OR2W3_chimp
OR2W3DP_chimp
OR2W3_human
OR51E1_human
0.2

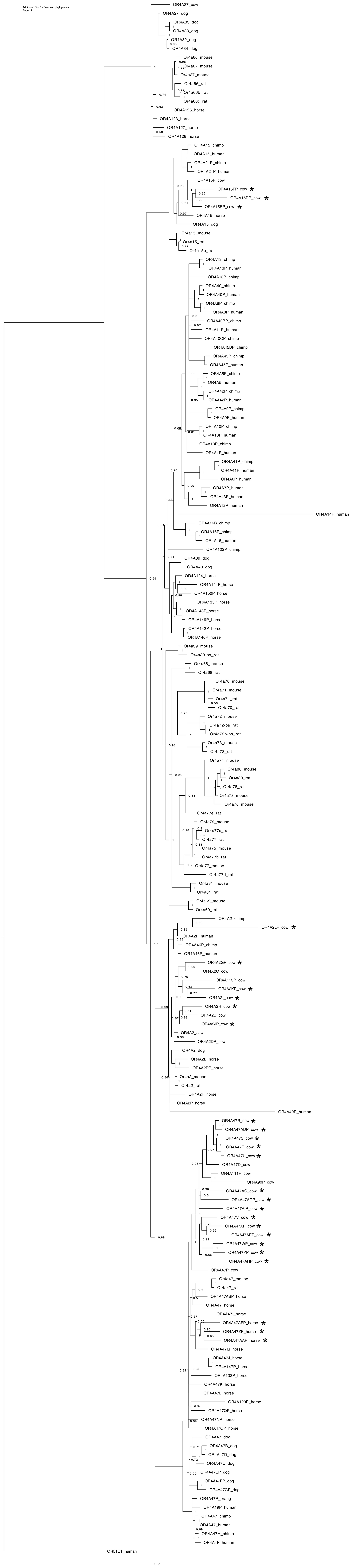
OR4A27_cow
OR4A27_dog
OR4A33_dog
OR4A83_dog
OR4A82_dog
OR4A84_dog
Or4a66_mouse
Or4a67_mouse
Or4a27_mouse
Or4a66_rat
Or4a66b_rat
Or4a66c_rat
OR4A126_horse
OR4A123_horse
OR4A127_horse
OR4A128_horse
OR4A15_chimp
OR4A15_human
OR4A21P_chimp
OR4A21P_human
OR4A15P_cow
OR4A15FP_cow
OR4A15DP_cow
OR4A15EP_cow
OR4A15_horse
OR4A15_dog
Or4a15_mouse
Or4a15_rat
Or4a15b_rat
OR4A13_chimp
OR4A13P_human
OR4A13B_chimp
OR4A40_chimp
OR4A40P_human
OR4A8P_chimp
OR4A8P_human
OR4A40BP_chimp
OR4A11P_human
OR4A40CP_chimp
OR4A45BP_chimp
OR4A45P_chimp
OR4A45P_human
OR4A5P_chimp
OR4A5_human
OR4A42P_chimp
OR4A42P_human
OR4A9P_chimp
OR4A9P_human
OR4A10P_chimp
OR4A10P_human
OR4A13P_chimp
OR4A1P_human
OR4A41P_chimp
OR4A41P_human
OR4A6P_human
OR4A7P_human
OR4A43P_human
OR4A12P_human
OR4A14P_human
OR4A16B_chimp
OR4A16P_chimp
OR4A16_human
OR4A122P_chimp
OR4A39_dog
OR4A40_dog
OR4A124_horse
OR4A144P_horse
OR4A150P_horse
OR4A135P_horse
OR4A148P_horse
OR4A149P_horse
OR4A142P_horse
OR4A146P_horse
Or4a39_mouse
Or4a39-ps_rat
Or4a68_mouse
Or4a68_rat
Or4a70_mouse
Or4a71_mouse
Or4a71_rat
Or4a70_rat
Or4a72_mouse
Or4a72-ps_rat
Or4a72b-ps_rat
Or4a73_mouse
Or4a73_rat
Or4a74_mouse
Or4a80_mouse
Or4a80_rat
Or4a78_rat
Or4a78_mouse
Or4a76_mouse
Or4a77e_rat
Or4a79_mouse
Or4a77c_rat
Or4a77_rat
Or4a75_mouse
Or4a77b_rat
Or4a77_mouse
Or4a77d_rat
Or4a81_mouse
Or4a81_rat
Or4a69_mouse
Or4a69_rat
OR4A2_chimp
OR4A2LP_cow
OR4A2P_human
OR4A46P_chimp
OR4A46P_human
OR4A2GP_cow
OR4A2C_cow
OR4A113P_cow
OR4A2KP_cow
OR4A2I_cow
OR4A2H_cow
OR4A2B_cow
OR4A2JP_cow
OR4A2_cow
OR4A2DP_cow
OR4A2_dog
OR4A2E_horse
OR4A2DP_horse
Or4a2_mouse
Or4a2_rat
OR4A2F_horse
OR4A2P_horse
OR4A49P_human
OR4A47R_cow
OR4A47ADP_cow
OR4A47S_cow
OR4A47T_cow
OR4A47U_cow
OR4A47D_cow
OR4A111P_cow
OR4A90P_cow
OR4A47AC_cow
OR4A47AGP_cow
OR4A47AIP_cow
OR4A47V_cow
OR4A47XP_cow
OR4A47AEP_cow
OR4A47WP_cow
OR4A47YP_cow
OR4A47AHP_cow
OR4A47P_cow
Or4a47_mouse
Or4a47_rat
OR4A47ABP_horse
OR4A47_horse
OR4A47I_horse
OR4A47AFP_horse
OR4A47ZP_horse
OR4A47AAP_horse
OR4A47M_horse
OR4A47J_horse
OR4A147P_horse
OR4A132P_horse
OR4A47K_horse
OR4A47L_horse
OR4A129P_horse
OR4A47QP_horse
OR4A47NP_horse
OR4A47OP_horse
OR4A47_dog
OR4A47B_dog
OR4A47D_dog
OR4A47C_dog
OR4A47EP_dog
OR4A47FP_dog
OR4A47GP_dog
OR4A47P_orang
OR4A19P_human
OR4A47_chimp
OR4A47_human
OR4A47H_chimp
OR4A4P_human
OR51E1_human
0.2

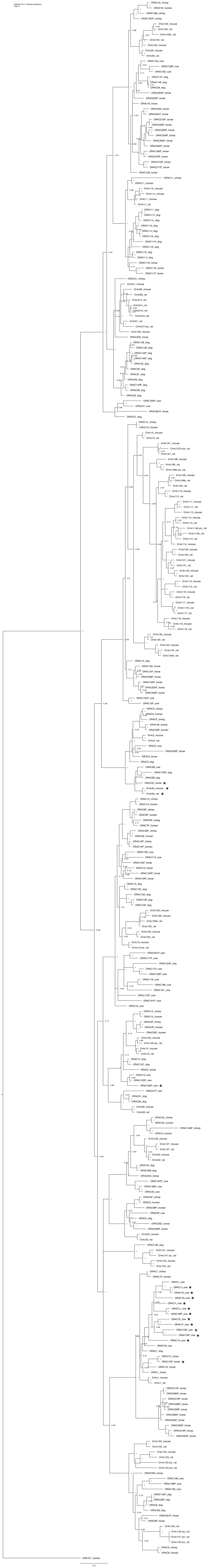

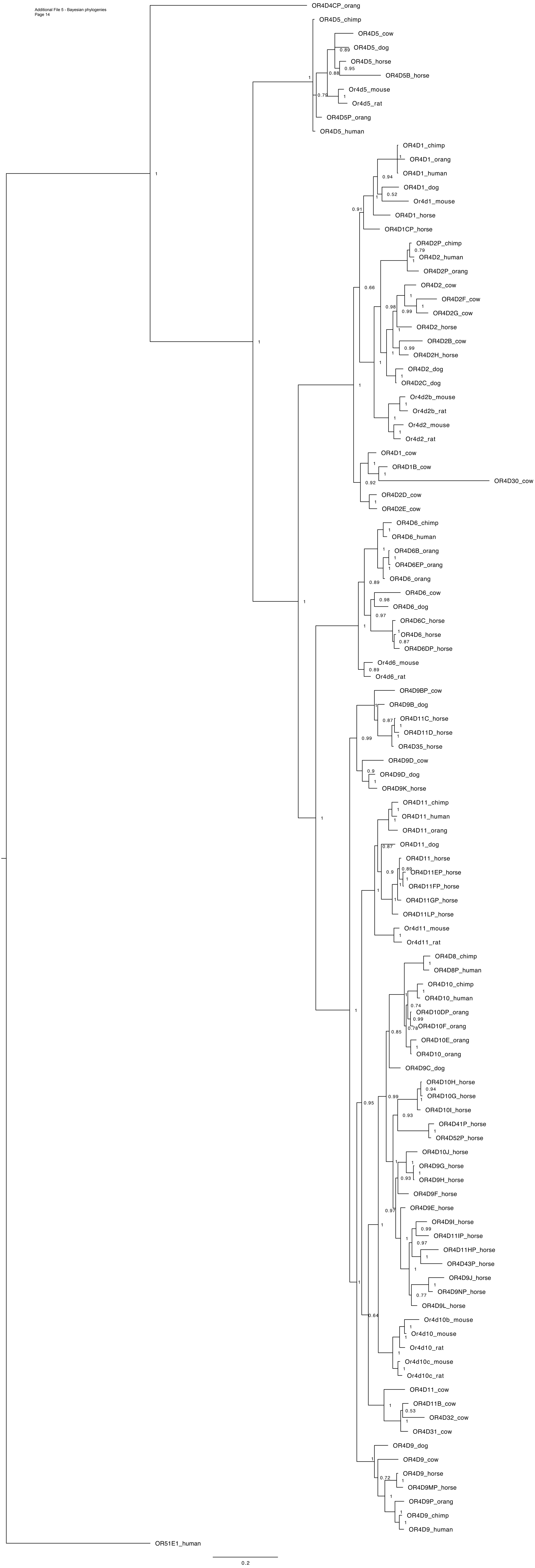
OR4D4CP_orang
OR4D5_chimp
OR4D5_cow
OR4D5_dog
OR4D5_horse
OR4D5B_horse
Or4d5_mouse
Or4d5_rat
OR4D5P_orang
OR4D5_human
OR4D1_chimp
OR4D1_orang
OR4D1_human
OR4D1_dog
Or4d1_mouse
OR4D1_horse
OR4D1CP_horse
OR4D2P_chimp
OR4D2_human
OR4D2P_orang
OR4D2_cow
OR4D2F_cow
OR4D2G_cow
OR4D2_horse
OR4D2B_cow
OR4D2H_horse
OR4D2_dog
OR4D2C_dog
Or4d2b_mouse
Or4d2b_rat
Or4d2_mouse
Or4d2_rat
OR4D1_cow
OR4D1B_cow
OR4D30_cow
OR4D2D_cow
OR4D2E_cow
OR4D6_chimp
OR4D6_human
OR4D6B_orang
OR4D6EP_orang
OR4D6_orang
OR4D6_cow
OR4D6_dog
OR4D6C_horse
OR4D6_horse
OR4D6DP_horse
Or4d6_mouse
Or4d6_rat
OR4D9BP_cow
OR4D9B_dog
OR4D11C_horse
OR4D11D_horse
OR4D35_horse
OR4D9D_cow
OR4D9D_dog
OR4D9K_horse
OR4D11_chimp
OR4D11_human
OR4D11_orang
OR4D11_dog
OR4D11_horse
OR4D11EP_horse
OR4D11FP_horse
OR4D11GP_horse
OR4D11LP_horse
Or4d11_mouse
Or4d11_rat
OR4D8_chimp
OR4D8P_human
OR4D10_chimp
OR4D10_human
OR4D10DP_orang
OR4D10F_orang
OR4D10E_orang
OR4D10_orang
OR4D9C_dog
OR4D10H_horse
OR4D10G_horse
OR4D10I_horse
OR4D41P_horse
OR4D52P_horse
OR4D10J_horse
OR4D9G_horse
OR4D9H_horse
OR4D9F_horse
OR4D9E_horse
OR4D9I_horse
OR4D11IP_horse
OR4D11HP_horse
OR4D43P_horse
OR4D9J_horse
OR4D9NP_horse
OR4D9L_horse
Or4d10b_mouse
Or4d10_mouse
Or4d10_rat
Or4d10c_mouse
Or4d10c_rat
OR4D11_cow
OR4D11B_cow
OR4D32_cow
OR4D31_cow
OR4D9_dog
OR4D9_cow
OR4D9_horse
OR4D9MP_horse
OR4D9P_orang
OR4D9_chimp
OR4D9_human
OR51E1_human
0.2

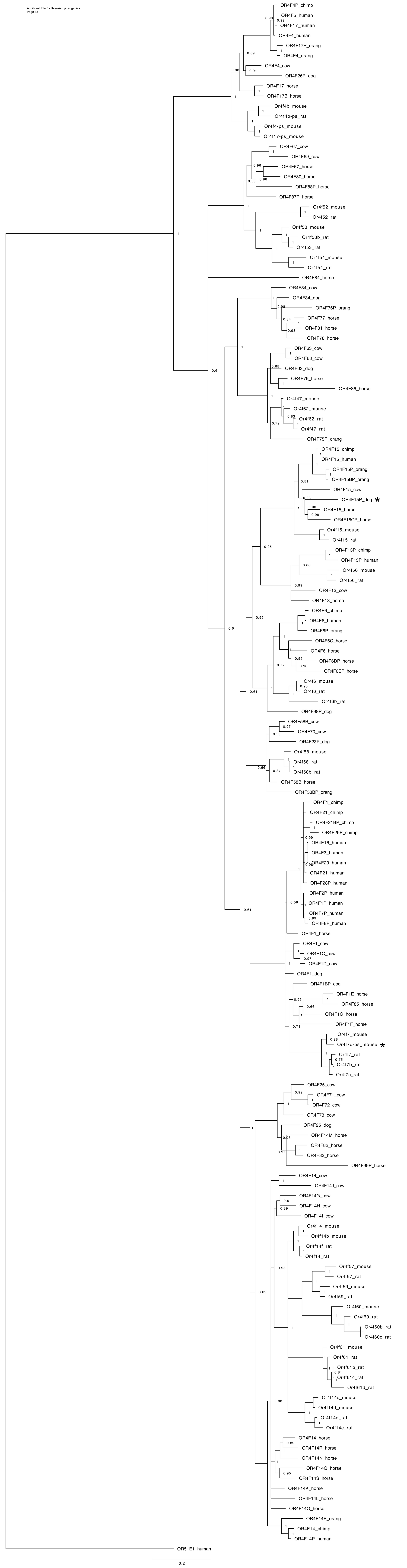
OR4F4P_chimp
OR4F5_human
OR4F17_human
OR4F4_human
OR4F17P_orang
OR4F4_orang
OR4F4_cow
OR4F26P_dog
OR4F17_horse
OR4F17B_horse
Or4f4b_mouse
Or4f4b-ps_rat
Or4f4-ps_mouse
Or4f17-ps_mouse
OR4F67_cow
OR4F69_cow
OR4F67_horse
OR4F80_horse
OR4F88P_horse
OR4F87P_horse
Or4f52_mouse
Or4f52_rat
Or4f53_mouse
Or4f53b_rat
Or4f53_rat
Or4f54_mouse
Or4f54_rat
OR4F84_horse
OR4F34_cow
OR4F34_dog
OR4F76P_orang
OR4F77_horse
OR4F81_horse
OR4F78_horse
OR4F63_cow
OR4F68_cow
OR4F63_dog
OR4F79_horse
OR4F86_horse
Or4f47_mouse
Or4f62_mouse
Or4f62_rat
Or4f47_rat
OR4F75P_orang
OR4F15_chimp
OR4F15_human
OR4F15P_orang
OR4F15BP_orang
OR4F15_cow
OR4F15P_dog *
OR4F15_horse
OR4F15CP_horse
Or4f15_mouse
Or4f15_rat
OR4F13P_chimp
OR4F13P_human
Or4f56_mouse
Or4f56_rat
OR4F13_cow
OR4F13_horse
OR4F6_chimp
OR4F6_human
OR4F6P_orang
OR4F6C_horse
OR4F6_horse
OR4F6DP_horse
OR4F6EP_horse
Or4f6_mouse
Or4f6_rat
Or4f6b_rat
OR4F98P_dog
OR4F58B_cow
OR4F70_cow
OR4F23P_dog
Or4f58_mouse
Or4f58_rat
Or4f58b_rat
OR4F58B_horse
OR4F58BP_orang
OR4F1_chimp
OR4F21_chimp
OR4F21BP_chimp
OR4F29P_chimp
OR4F16_human
OR4F3_human
OR4F29_human
OR4F21_human
OR4F28P_human
OR4F2P_human
OR4F1P_human
OR4F7P_human
OR4F8P_human
OR4F1_horse
OR4F1_cow
OR4F1C_cow
OR4F1D_cow
OR4F1_dog
OR4F1BP_dog
OR4F1E_horse
OR4F85_horse
OR4F1G_horse
OR4F1F_horse
Or4f7_mouse
Or4f7d-ps_mouse *
Or4f7_rat
Or4f7b_rat
Or4f7c_rat
OR4F25_cow
OR4F71_cow
OR4F72_cow
OR4F73_cow
OR4F25_dog
OR4F14M_horse
OR4F82_horse
OR4F83_horse
OR4F99P_horse
OR4F14_cow
OR4F14J_cow
OR4F14G_cow
OR4F14H_cow
OR4F14I_cow
Or4f14_mouse
Or4f14b_mouse
Or4f14f_rat
Or4f14_rat
Or4f57_mouse
Or4f57_rat
Or4f59_mouse
Or4f59_rat
Or4f60_mouse
Or4f60_rat
Or4f60b_rat
Or4f60c_rat
Or4f61_mouse
Or4f61_rat
Or4f61b_rat
Or4f61c_rat
Or4f61d_rat
Or4f14c_mouse
Or4f14d_mouse
Or4f14d_rat
Or4f14e_rat
OR4F14_horse
OR4F14R_horse
OR4F14N_horse
OR4F14Q_horse
OR4F14S_horse
OR4F14K_horse
OR4F14L_horse
OR4F14O_horse
OR4F14P_orang
OR4F14_chimp
OR4F14P_human
OR51E1_human
0.2

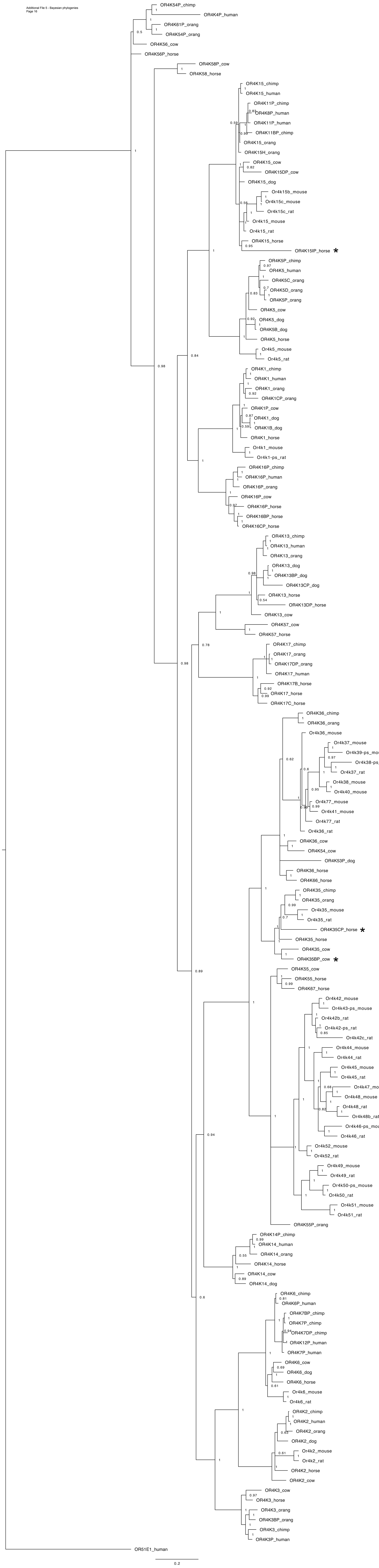
OR4K54P_chimp
OR4K4P_human
OR4K61P_orang
OR4K54P_orang
OR4K56_cow
OR4K56P_horse
OR4K58P_cow
OR4K58_horse
OR4K15_chimp
OR4K15_human
OR4K11P_chimp
OR4K8P_human
OR4K11P_human
OR4K11BP_chimp
OR4K15_orang
OR4K15H_orang
OR4K15_cow
OR4K15DP_cow
OR4K15_dog
Or4k15b_mouse
Or4k15c_mouse
Or4k15c_rat
Or4k15_mouse
Or4k15_rat
OR4K15_horse
OR4K15IP_horse *
OR4K5P_chimp
OR4K5_human
OR4K5C_orang
OR4K5D_orang
OR4K5P_orang
OR4K5_cow
OR4K5_dog
OR4K5B_dog
OR4K5_horse
Or4k5_mouse
Or4k5_rat
OR4K1_chimp
OR4K1_human
OR4K1_orang
OR4K1CP_orang
OR4K1P_cow
OR4K1_dog
OR4K1B_dog
OR4K1_horse
Or4k1_mouse
Or4k1-ps_rat
OR4K16P_chimp
OR4K16P_human
OR4K16P_orang
OR4K16P_cow
OR4K16P_horse
OR4K16BP_horse
OR4K16CP_horse
OR4K13_chimp
OR4K13_human
OR4K13_orang
OR4K13_dog
OR4K13BP_dog
OR4K13CP_dog
OR4K13_horse
OR4K13DP_horse
OR4K13_cow
OR4K57_cow
OR4K57_horse
OR4K17_chimp
OR4K17_orang
OR4K17DP_orang
OR4K17_human
OR4K17B_horse
OR4K17_horse
OR4K17C_horse
OR4K36_chimp
OR4K36_orang
Or4k36_mouse
Or4k37_mouse
Or4k39-ps_mouse
Or4k38-ps_rat
Or4k37_rat
Or4k38_mouse
Or4k40_mouse
Or4k77_mouse
Or4k41_mouse
Or4k77_rat
Or4k36_rat
OR4K36_cow
OR4K54_cow
OR4K53P_dog
OR4K36_horse
OR4K66_horse
OR4K35_chimp
OR4K35_orang
Or4k35_mouse
Or4k35_rat
OR4K35CP_horse *
OR4K35_horse
OR4K35_cow
OR4K35BP_cow *
OR4K55_cow
OR4K55_horse
OR4K67_horse
Or4k42_mouse
Or4k43-ps_mouse
Or4k42b_rat
Or4k42-ps_rat
Or4k42c_rat
Or4k44_mouse
Or4k44_rat
Or4k45_mouse
Or4k45_rat
Or4k47_mouse
Or4k48_mouse
Or4k48_rat
Or4k48b_rat
Or4k46-ps_mouse
Or4k46_rat
Or4k52_mouse
Or4k52_rat
Or4k49_mouse
Or4k49_rat
Or4k50-ps_mouse
Or4k50_rat
Or4k51_mouse
Or4k51_rat
OR4K55P_orang
OR4K14P_chimp
OR4K14_human
OR4K14_orang
OR4K14_horse
OR4K14_cow
OR4K14_dog
OR4K6_chimp
OR4K6P_human
OR4K7BP_chimp
OR4K7P_chimp
OR4K7DP_chimp
OR4K12P_human
OR4K7P_human
OR4K6_cow
OR4K6_dog
OR4K6_horse
Or4k6_mouse
Or4k6_rat
OR4K2_chimp
OR4K2_human
OR4K2_orang
OR4K2_dog
Or4k2_mouse
Or4k2_rat
OR4K2_horse
OR4K2_cow
OR4K3_cow
OR4K3_horse
OR4K3_orang
OR4K3BP_orang
OR4K3_chimp
OR4K3P_human
OR51E1_human
0.2

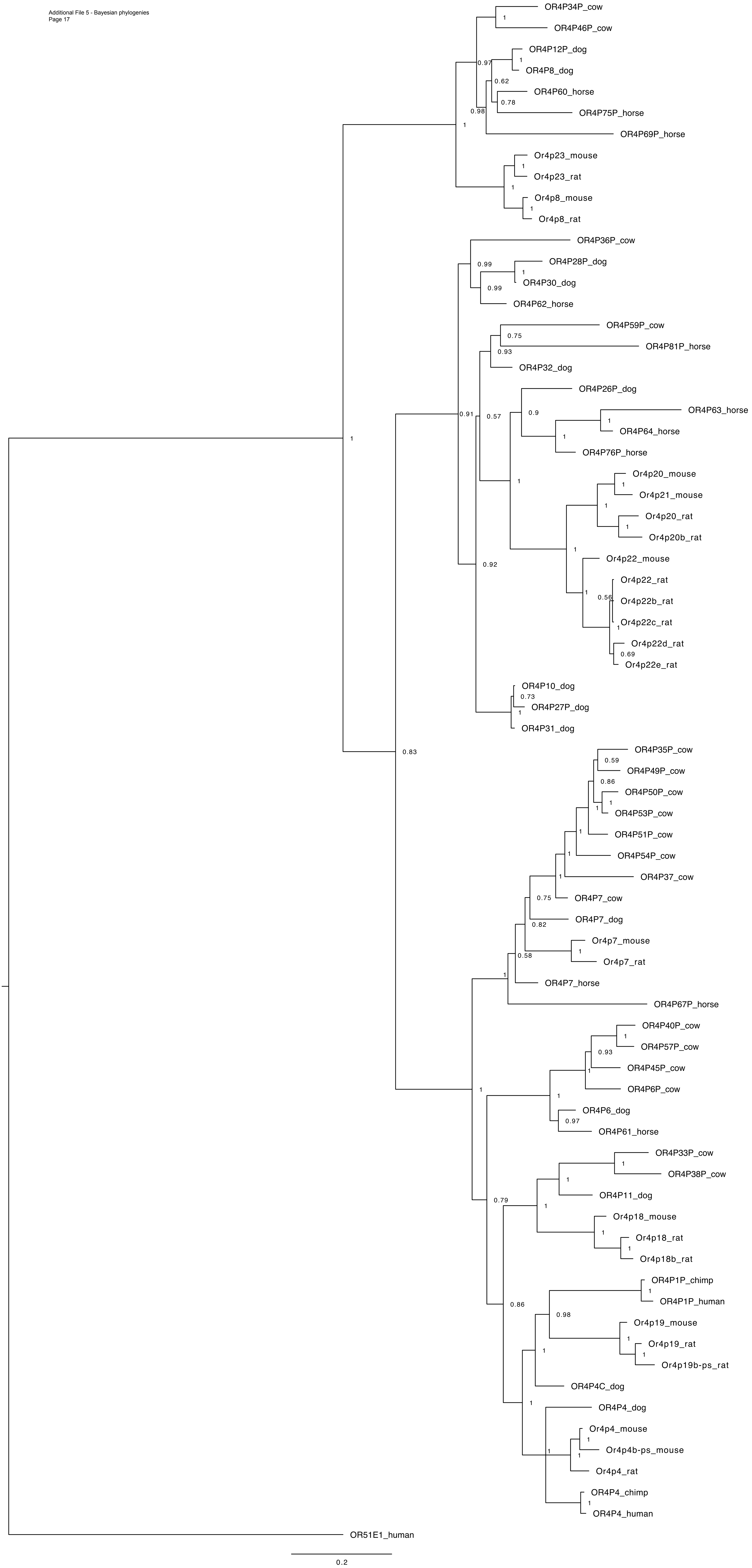
OR4P34P_cow
OR4P46P_cow
OR4P12P_dog
OR4P8_dog
OR4P60_horse
OR4P75P_horse
OR4P69P_horse
Or4p23_mouse
Or4p23_rat
Or4p8_mouse
Or4p8_rat
OR4P36P_cow
OR4P28P_dog
OR4P30_dog
OR4P62_horse
OR4P59P_cow
OR4P81P_horse
OR4P32_dog
OR4P26P_dog
OR4P63_horse
OR4P64_horse
OR4P76P_horse
Or4p20_mouse
Or4p21_mouse
Or4p20_rat
Or4p20b_rat
Or4p22_mouse
Or4p22_rat
Or4p22b_rat
Or4p22c_rat
Or4p22d_rat
Or4p22e_rat
OR4P10_dog
OR4P27P_dog
OR4P31_dog
OR4P35P_cow
OR4P49P_cow
OR4P50P_cow
OR4P53P_cow
OR4P51P_cow
OR4P54P_cow
OR4P37_cow
OR4P7_cow
OR4P7_dog
Or4p7_mouse
Or4p7_rat
OR4P7_horse
OR4P67P_horse
OR4P40P_cow
OR4P57P_cow
OR4P45P_cow
OR4P6P_cow
OR4P6_dog
OR4P61_horse
OR4P33P_cow
OR4P38P_cow
OR4P11_dog
Or4p18_mouse
Or4p18_rat
Or4p18b_rat
OR4P1P_chimp
OR4P1P_human
Or4p19_mouse
Or4p19_rat
Or4p19b-ps_rat
OR4P4C_dog
OR4P4_dog
Or4p4_mouse
Or4p4b-ps_mouse
Or4p4_rat
OR4P4_chimp
OR4P4_human
OR51E1_human
0.97
0.62
0.78
0.98
0.99
0.99
0.75
0.93
0.91
0.57
0.9
0.56
0.69
0.92
0.73
0.83
0.59
0.86
0.75
0.82
0.58
0.93
0.97
0.79
0.98
0.86
0.2

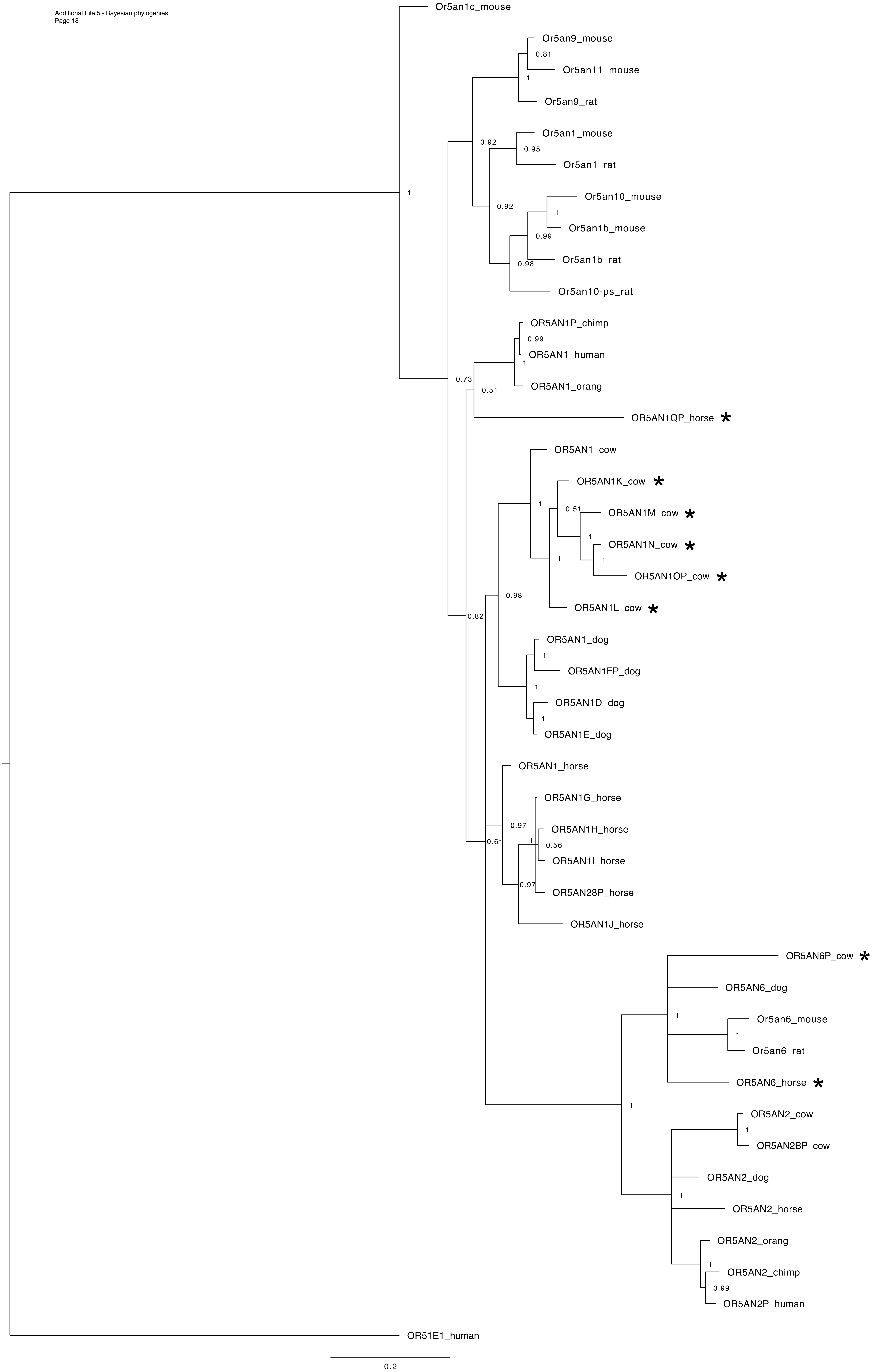
Or5an1c_mouse
Or5an9_mouse
Or5an11_mouse
Or5an9_rat
Or5an1_mouse
Or5an1_rat
Or5an10_mouse
Or5an1b_mouse
Or5an1b_rat
Or5an10-ps_rat
OR5AN1P_chimp
OR5AN1_human
OR5AN1_orang
OR5AN1QP_horse *
OR5AN1_cow
OR5AN1K_cow *
OR5AN1M_cow *
OR5AN1N_cow *
OR5AN1OP_cow *
OR5AN1L_cow *
OR5AN1_dog
OR5AN1FP_dog
OR5AN1D_dog
OR5AN1E_dog
OR5AN1_horse
OR5AN1G_horse
OR5AN1H_horse
OR5AN1I_horse
OR5AN28P_horse
OR5AN1J_horse
OR5AN6P_cow *
OR5AN6_dog
Or5an6_mouse
Or5an6_rat
OR5AN6_horse *
OR5AN2_cow
OR5AN2BP_cow
OR5AN2_dog
OR5AN2_horse
OR5AN2_orang
OR5AN2_chimp
OR5AN2P_human
OR51E1_human
0.2

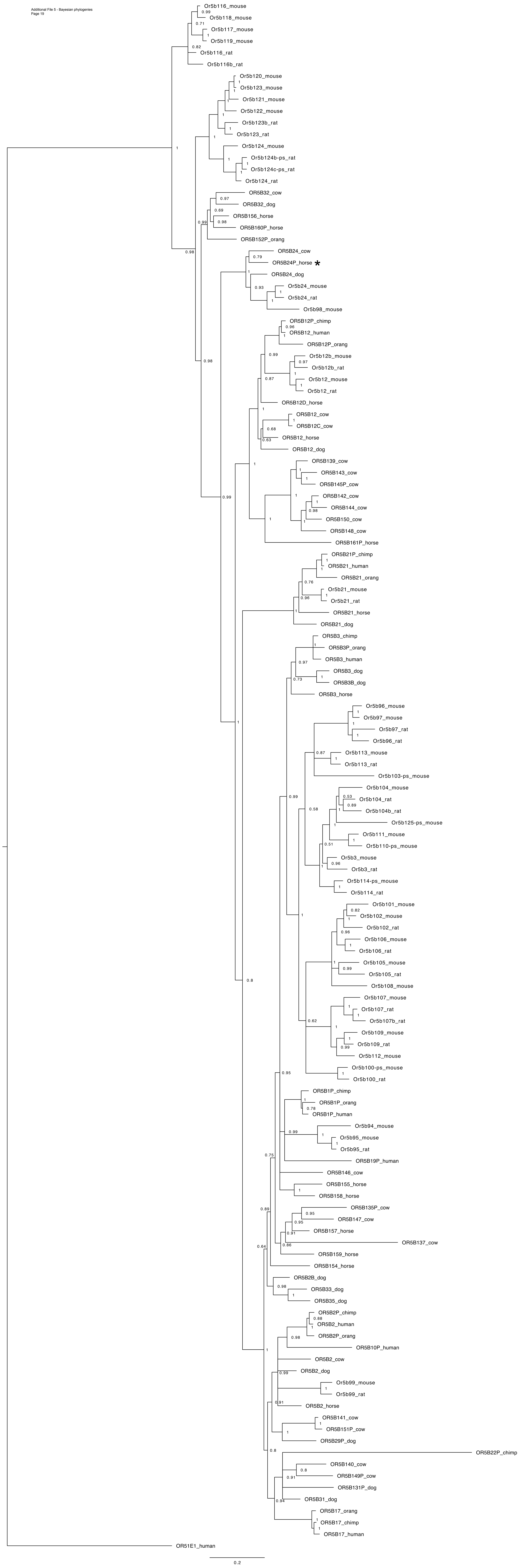
Or5b116_mouse
Or5b118_mouse
Or5b117_mouse
Or5b119_mouse
Or5b116_rat
Or5b116b_rat
Or5b120_mouse
Or5b123_mouse
Or5b121_mouse
Or5b122_mouse
Or5b123b_rat
Or5b123_rat
Or5b124_mouse
Or5b124b-ps_rat
Or5b124c-ps_rat
Or5b124_rat
OR5B32_cow
OR5B32_dog
OR5B156_horse
OR5B160P_horse
OR5B152P_orang
OR5B24_cow
OR5B24P_horse *
OR5B24_dog
Or5b24_mouse
Or5b24_rat
Or5b98_mouse
OR5B12P_chimp
OR5B12_human
OR5B12P_orang
Or5b12b_mouse
Or5b12b_rat
Or5b12_mouse
Or5b12_rat
OR5B12D_horse
OR5B12_cow
OR5B12C_cow
OR5B12_horse
OR5B12_dog
OR5B139_cow
OR5B143_cow
OR5B145P_cow
OR5B142_cow
OR5B144_cow
OR5B150_cow
OR5B148_cow
OR5B161P_horse
OR5B21P_chimp
OR5B21_human
OR5B21_orang
Or5b21_mouse
Or5b21_rat
OR5B21_horse
OR5B21_dog
OR5B3_chimp
OR5B3P_orang
OR5B3_human
OR5B3_dog
OR5B3B_dog
OR5B3_horse
Or5b96_mouse
Or5b97_mouse
Or5b97_rat
Or5b96_rat
Or5b113_mouse
Or5b113_rat
Or5b103-ps_mouse
Or5b104_mouse
Or5b104_rat
Or5b104b_rat
Or5b125-ps_mouse
Or5b111_mouse
Or5b110-ps_mouse
Or5b3_mouse
Or5b3_rat
Or5b114-ps_mouse
Or5b114_rat
Or5b101_mouse
Or5b102_mouse
Or5b102_rat
Or5b106_mouse
Or5b106_rat
Or5b105_mouse
Or5b105_rat
Or5b108_mouse
Or5b107_mouse
Or5b107_rat
Or5b107b_rat
Or5b109_mouse
Or5b109_rat
Or5b112_mouse
Or5b100-ps_mouse
Or5b100_rat
OR5B1P_chimp
OR5B1P_orang
OR5B1P_human
Or5b94_mouse
Or5b95_mouse
Or5b95_rat
OR5B19P_human
OR5B146_cow
OR5B155_horse
OR5B158_horse
OR5B135P_cow
OR5B147_cow
OR5B157_horse
OR5B137_cow
OR5B159_horse
OR5B154_horse
OR5B2B_dog
OR5B33_dog
OR5B35_dog
OR5B2P_chimp
OR5B2_human
OR5B2P_orang
OR5B10P_human
OR5B2_cow
OR5B2_dog
Or5b99_mouse
Or5b99_rat
OR5B2_horse
OR5B141_cow
OR5B151P_cow
OR5B29P_dog
OR5B22P_chimp
OR5B140_cow
OR5B149P_cow
OR5B131P_dog
OR5B31_dog
OR5B17_orang
OR5B17_chimp
OR5B17_human
OR51E1_human
0.2

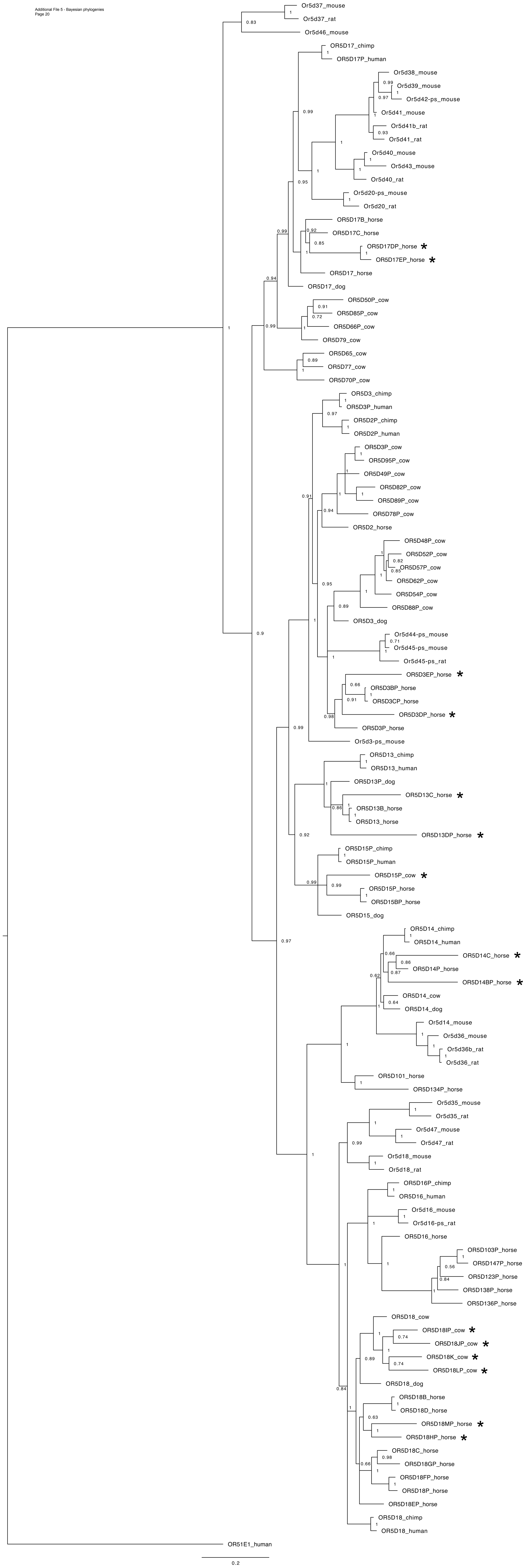
Or5d37_mouse
Or5d37_rat
Or5d46_mouse
OR5D17_chimp
OR5D17P_human
Or5d38_mouse
Or5d39_mouse
Or5d42-ps_mouse
Or5d41_mouse
Or5d41b_rat
Or5d41_rat
Or5d40_mouse
Or5d43_mouse
Or5d40_rat
Or5d20-ps_mouse
Or5d20_rat
OR5D17B_horse
OR5D17C_horse
OR5D17DP_horse *
OR5D17EP_horse *
OR5D17_horse
OR5D17_dog
OR5D50P_cow
OR5D85P_cow
OR5D66P_cow
OR5D79_cow
OR5D65_cow
OR5D77_cow
OR5D70P_cow
OR5D3_chimp
OR5D3P_human
OR5D2P_chimp
OR5D2P_human
OR5D3P_cow
OR5D95P_cow
OR5D49P_cow
OR5D82P_cow
OR5D89P_cow
OR5D78P_cow
OR5D2_horse
OR5D48P_cow
OR5D52P_cow
OR5D57P_cow
OR5D62P_cow
OR5D54P_cow
OR5D88P_cow
OR5D3_dog
Or5d44-ps_mouse
Or5d45-ps_mouse
Or5d45-ps_rat
OR5D3EP_horse *
OR5D3BP_horse
OR5D3CP_horse
OR5D3DP_horse *
OR5D3P_horse
Or5d3-ps_mouse
OR5D13_chimp
OR5D13_human
OR5D13P_dog
OR5D13C_horse *
OR5D13B_horse
OR5D13_horse
OR5D13DP_horse *
OR5D15P_chimp
OR5D15P_human
OR5D15P_cow *
OR5D15P_horse
OR5D15BP_horse
OR5D15_dog
OR5D14_chimp
OR5D14_human
OR5D14C_horse *
OR5D14P_horse
OR5D14BP_horse *
OR5D14_cow
OR5D14_dog
Or5d14_mouse
Or5d36_mouse
Or5d36b_rat
Or5d36_rat
OR5D101_horse
OR5D134P_horse
Or5d35_mouse
Or5d35_rat
Or5d47_mouse
Or5d47_rat
Or5d18_mouse
Or5d18_rat
OR5D16P_chimp
OR5D16_human
Or5d16_mouse
Or5d16-ps_rat
OR5D16_horse
OR5D103P_horse
OR5D147P_horse
OR5D123P_horse
OR5D138P_horse
OR5D136P_horse
OR5D18_cow
OR5D18IP_cow *
OR5D18JP_cow *
OR5D18K_cow *
OR5D18LP_cow *
OR5D18_dog
OR5D18B_horse
OR5D18D_horse
OR5D18MP_horse *
OR5D18HP_horse *
OR5D18C_horse
OR5D18GP_horse
OR5D18FP_horse
OR5D18P_horse
OR5D18EP_horse
OR5D18_chimp
OR5D18_human
OR51E1_human
0.2

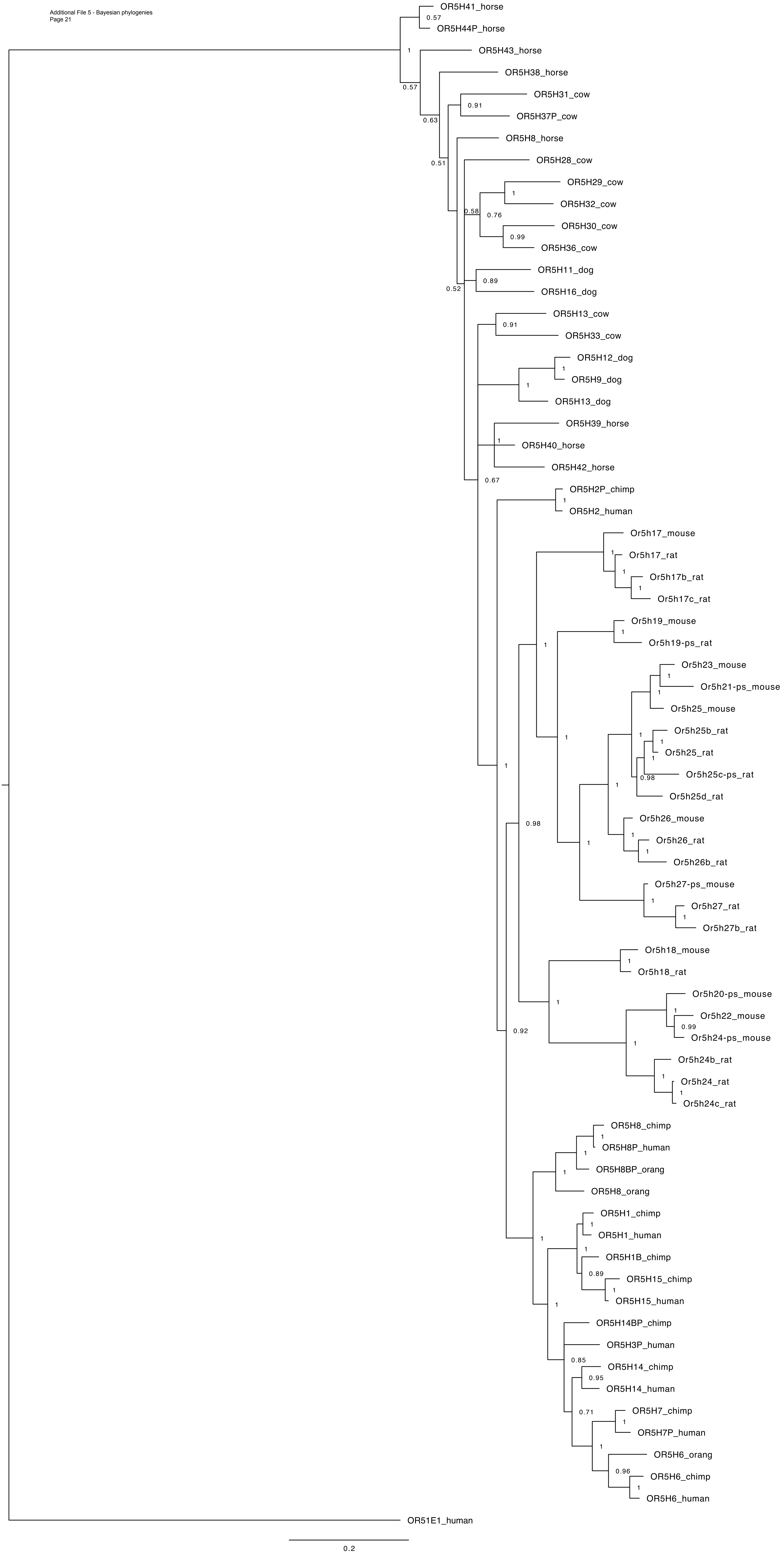
OR5H41_horse
OR5H44P_horse
OR5H43_horse
OR5H38_horse
OR5H31_cow
OR5H37P_cow
OR5H8_horse
OR5H28_cow
OR5H29_cow
OR5H32_cow
OR5H30_cow
OR5H36_cow
OR5H11_dog
OR5H16_dog
OR5H13_cow
OR5H33_cow
OR5H12_dog
OR5H9_dog
OR5H13_dog
OR5H39_horse
OR5H40_horse
OR5H42_horse
OR5H2P_chimp
OR5H2_human
Or5h17_mouse
Or5h17_rat
Or5h17b_rat
Or5h17c_rat
Or5h19_mouse
Or5h19-ps_rat
Or5h23_mouse
Or5h21-ps_mouse
Or5h25_mouse
Or5h25b_rat
Or5h25_rat
Or5h25c-ps_rat
Or5h25d_rat
Or5h26_mouse
Or5h26_rat
Or5h26b_rat
Or5h27-ps_mouse
Or5h27_rat
Or5h27b_rat
Or5h18_mouse
Or5h18_rat
Or5h20-ps_mouse
Or5h22_mouse
Or5h24-ps_mouse
Or5h24b_rat
Or5h24_rat
Or5h24c_rat
OR5H8_chimp
OR5H8P_human
OR5H8BP_orang
OR5H8_orang
OR5H1_chimp
OR5H1_human
OR5H1B_chimp
OR5H15_chimp
OR5H15_human
OR5H14BP_chimp
OR5H3P_human
OR5H14_chimp
OR5H14_human
OR5H7_chimp
OR5H7P_human
OR5H6_orang
OR5H6_chimp
OR5H6_human
OR51E1_human
0.2

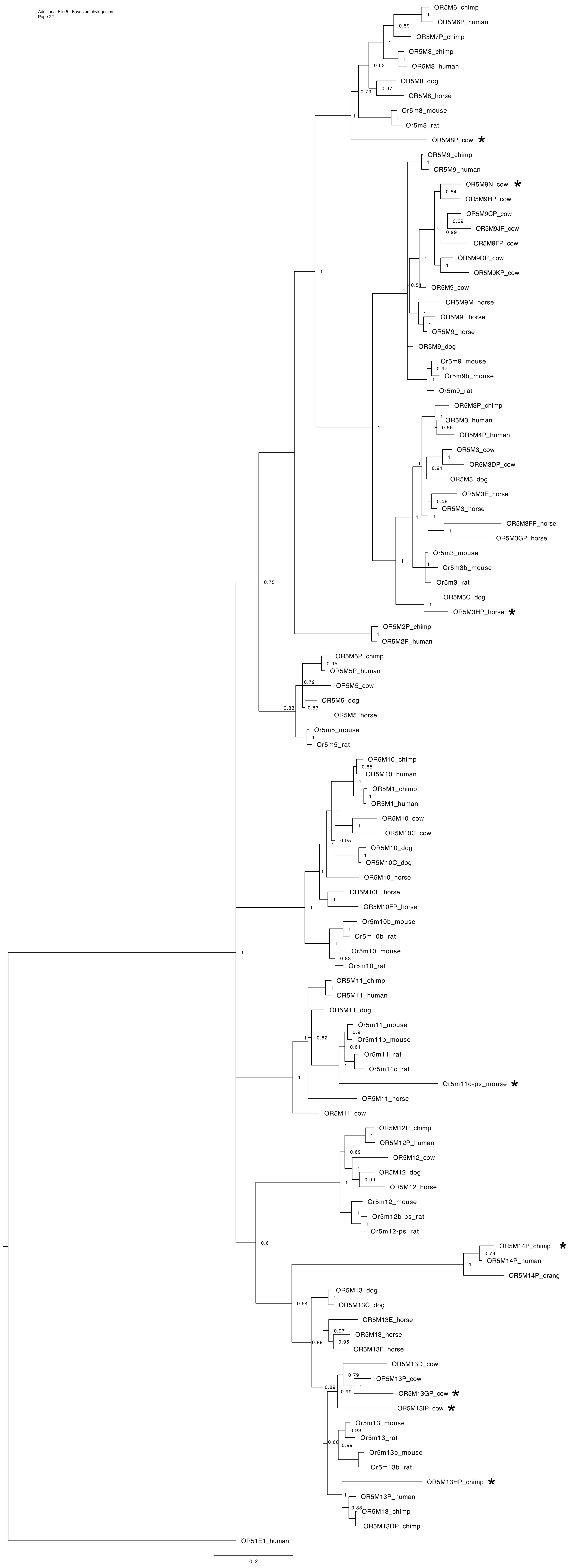
OR5M6_chimp
OR5M6P_human
OR5M7P_chimp
OR5M8_chimp
OR5M8_human
OR5M8_dog
OR5M8_horse
Or5m8_mouse
Or5m8_rat
OR5M8P_cow *
OR5M9_chimp
OR5M9_human
OR5M9N_cow *
OR5M9HP_cow
OR5M9CP_cow
OR5M9JP_cow
OR5M9FP_cow
OR5M9DP_cow
OR5M9KP_cow
OR5M9_cow
OR5M9M_horse
OR5M9I_horse
OR5M9_horse
OR5M9_dog
Or5m9_mouse
Or5m9b_mouse
Or5m9_rat
OR5M3P_chimp
OR5M3_human
OR5M4P_human
OR5M3_cow
OR5M3DP_cow
OR5M3_dog
OR5M3E_horse
OR5M3_horse
OR5M3FP_horse
OR5M3GP_horse
Or5m3_mouse
Or5m3b_mouse
Or5m3_rat
OR5M3C_dog
OR5M3HP_horse *
OR5M2P_chimp
OR5M2P_human
OR5M5P_chimp
OR5M5P_human
OR5M5_cow
OR5M5_dog
OR5M5_horse
Or5m5_mouse
Or5m5_rat
OR5M10_chimp
OR5M10_human
OR5M1_chimp
OR5M1_human
OR5M10_cow
OR5M10C_cow
OR5M10_dog
OR5M10C_dog
OR5M10_horse
OR5M10E_horse
OR5M10FP_horse
Or5m10b_mouse
Or5m10b_rat
Or5m10_mouse
Or5m10_rat
OR5M11_chimp
OR5M11_human
OR5M11_dog
Or5m11_mouse
Or5m11b_mouse
Or5m11_rat
Or5m11c_rat
Or5m11d-ps_mouse *
OR5M11_horse
OR5M11_cow
OR5M12P_chimp
OR5M12P_human
OR5M12_cow
OR5M12_dog
OR5M12_horse
Or5m12_mouse
Or5m12b-ps_rat
Or5m12-ps_rat
OR5M14P_chimp *
OR5M14P_human
OR5M14P_orang
OR5M13_dog
OR5M13C_dog
OR5M13E_horse
OR5M13_horse
OR5M13F_horse
OR5M13D_cow
OR5M13P_cow
OR5M13GP_cow *
OR5M13IP_cow *
Or5m13_mouse
Or5m13_rat
Or5m13b_mouse
Or5m13b_rat
OR5M13HP_chimp *
OR5M13P_human
OR5M13_chimp
OR5M13DP_chimp
OR51E1_human
0.2

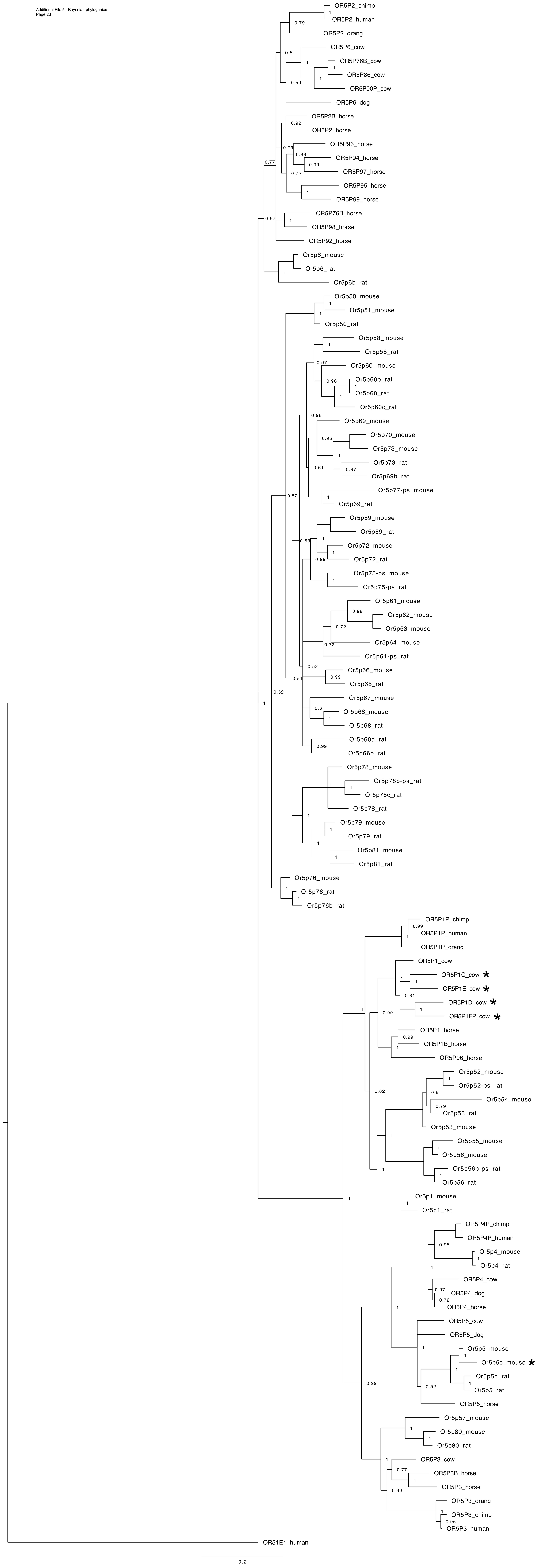
OR5P2_chimp
OR5P2_human
OR5P2_orang
OR5P6_cow
OR5P76B_cow
OR5P86_cow
OR5P90P_cow
OR5P6_dog
OR5P2B_horse
OR5P2_horse
OR5P93_horse
OR5P94_horse
OR5P97_horse
OR5P95_horse
OR5P99_horse
OR5P76B_horse
OR5P98_horse
OR5P92_horse
Or5p6_mouse
Or5p6_rat
Or5p6b_rat
Or5p50_mouse
Or5p51_mouse
Or5p50_rat
Or5p58_mouse
Or5p58_rat
Or5p60_mouse
Or5p60b_rat
Or5p60_rat
Or5p60c_rat
Or5p69_mouse
Or5p70_mouse
Or5p73_mouse
Or5p73_rat
Or5p69b_rat
Or5p77-ps_mouse
Or5p69_rat
Or5p59_mouse
Or5p59_rat
Or5p72_mouse
Or5p72_rat
Or5p75-ps_mouse
Or5p75-ps_rat
Or5p61_mouse
Or5p62_mouse
Or5p63_mouse
Or5p64_mouse
Or5p61-ps_rat
Or5p66_mouse
Or5p66_rat
Or5p67_mouse
Or5p68_mouse
Or5p68_rat
Or5p60d_rat
Or5p66b_rat
Or5p78_mouse
Or5p78b-ps_rat
Or5p78c_rat
Or5p78_rat
Or5p79_mouse
Or5p79_rat
Or5p81_mouse
Or5p81_rat
Or5p76_mouse
Or5p76_rat
Or5p76b_rat
OR5P1P_chimp
OR5P1P_human
OR5P1P_orang
OR5P1_cow
OR5P1C_cow *
OR5P1E_cow *
OR5P1D_cow *
OR5P1FP_cow *
OR5P1_horse
OR5P1B_horse
OR5P96_horse
Or5p52_mouse
Or5p52-ps_rat
Or5p54_mouse
Or5p53_rat
Or5p53_mouse
Or5p55_mouse
Or5p56_mouse
Or5p56b-ps_rat
Or5p56_rat
Or5p1_mouse
Or5p1_rat
OR5P4P_chimp
OR5P4P_human
Or5p4_mouse
Or5p4_rat
OR5P4_cow
OR5P4_dog
OR5P4_horse
OR5P5_cow
OR5P5_dog
Or5p5_mouse
Or5p5c_mouse *
Or5p5b_rat
Or5p5_rat
OR5P5_horse
Or5p57_mouse
Or5p80_mouse
Or5p80_rat
OR5P3_cow
OR5P3B_horse
OR5P3_horse
OR5P3_orang
OR5P3_chimp
OR5P3_human
OR51E1_human
0.2

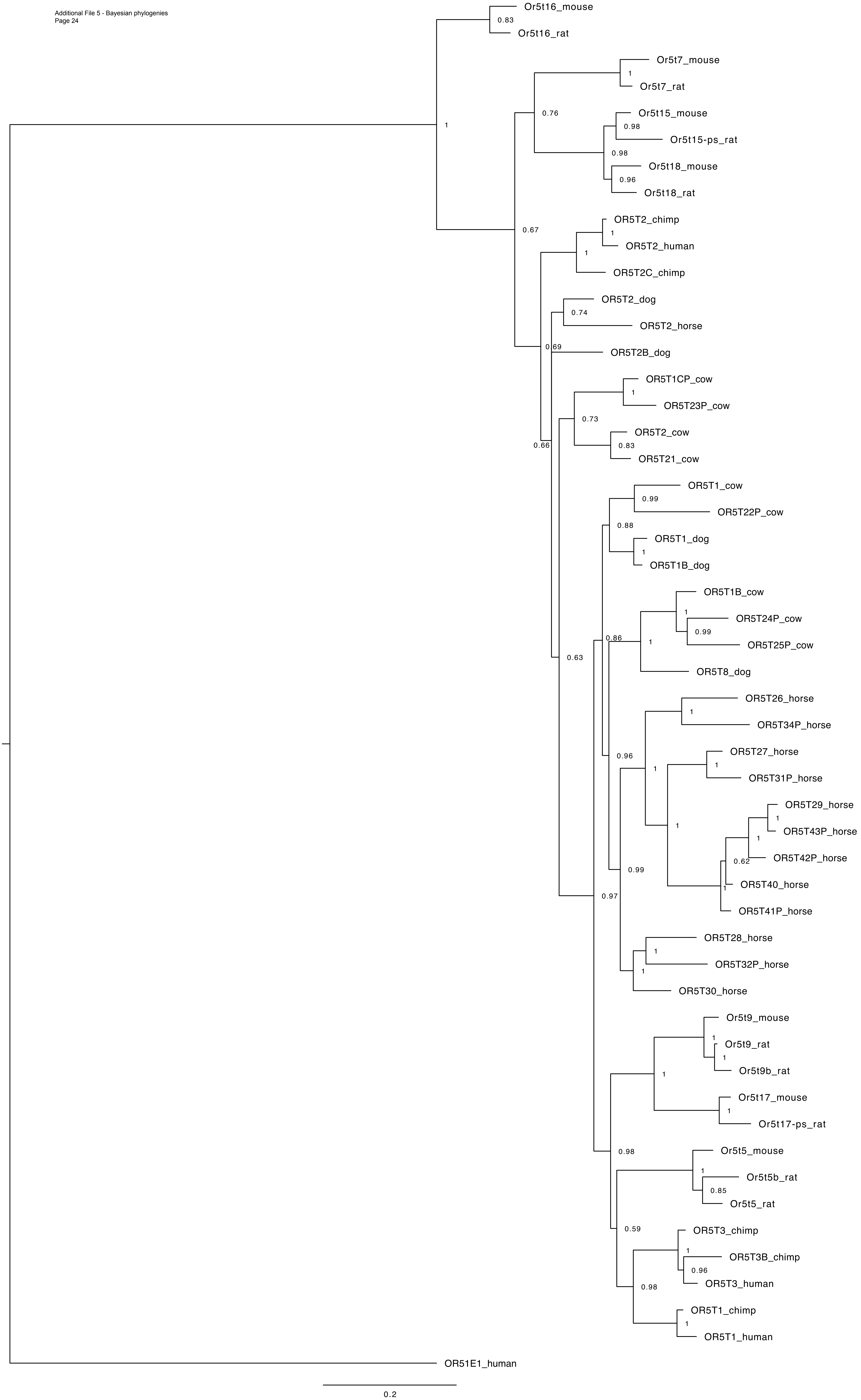
Or5t16_mouse
0.83
Or5t16_rat
Or5t7_mouse
1
Or5t7_rat
0.76
Or5t15_mouse
0.98
Or5t15-ps_rat
1
0.98
Or5t18_mouse
0.96
Or5t18_rat
OR5T2_chimp
1
OR5T2_human
0.67
1
OR5T2C_chimp
OR5T2_dog
0.74
OR5T2_horse
0.69
OR5T2B_dog
OR5T1CP_cow
1
OR5T23P_cow
0.73
OR5T2_cow
0.83
0.66
OR5T21_cow
OR5T1_cow
0.99
OR5T22P_cow
0.88
OR5T1_dog
1
OR5T1B_dog
OR5T1B_cow
1
OR5T24P_cow
0.99
0.86
1
OR5T25P_cow
0.63
OR5T8_dog
OR5T26_horse
1
OR5T34P_horse
0.96
OR5T27_horse
1
1
OR5T31P_horse
OR5T29_horse
1
1
OR5T43P_horse
0.62
OR5T42P_horse
0.99
OR5T40_horse
1
0.97
OR5T41P_horse
OR5T28_horse
1
OR5T32P_horse
1
OR5T30_horse
Or5t9_mouse
1
Or5t9_rat
1
1
Or5t9b_rat
Or5t17_mouse
1
Or5t17-ps_rat
0.98
Or5t5_mouse
1
Or5t5b_rat
0.85
Or5t5_rat
0.59
OR5T3_chimp
1
OR5T3B_chimp
0.96
0.98
OR5T3_human
OR5T1_chimp
1
OR5T1_human
OR51E1_human
0.2

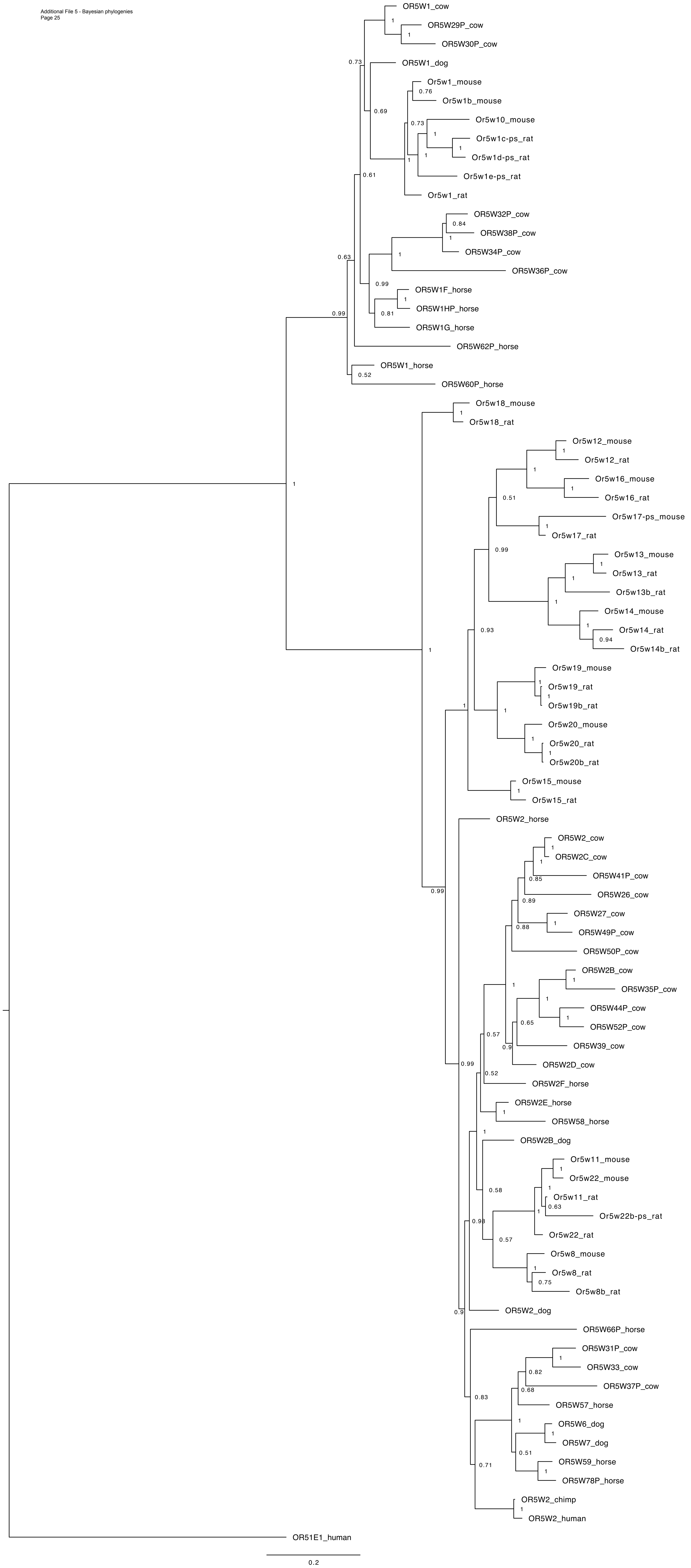
OR5W1_cow
OR5W29P_cow
OR5W30P_cow
OR5W1_dog
Or5w1_mouse
Or5w1b_mouse
Or5w10_mouse
Or5w1c-ps_rat
Or5w1d-ps_rat
Or5w1e-ps_rat
Or5w1_rat
OR5W32P_cow
OR5W38P_cow
OR5W34P_cow
OR5W36P_cow
OR5W1F_horse
OR5W1HP_horse
OR5W1G_horse
OR5W62P_horse
OR5W1_horse
OR5W60P_horse
Or5w18_mouse
Or5w18_rat
Or5w12_mouse
Or5w12_rat
Or5w16_mouse
Or5w16_rat
Or5w17-ps_mouse
Or5w17_rat
Or5w13_mouse
Or5w13_rat
Or5w13b_rat
Or5w14_mouse
Or5w14_rat
Or5w14b_rat
Or5w19_mouse
Or5w19_rat
Or5w19b_rat
Or5w20_mouse
Or5w20_rat
Or5w20b_rat
Or5w15_mouse
Or5w15_rat
OR5W2_horse
OR5W2_cow
OR5W2C_cow
OR5W41P_cow
OR5W26_cow
OR5W27_cow
OR5W49P_cow
OR5W50P_cow
OR5W2B_cow
OR5W35P_cow
OR5W44P_cow
OR5W52P_cow
OR5W39_cow
OR5W2D_cow
OR5W2F_horse
OR5W2E_horse
OR5W58_horse
OR5W2B_dog
Or5w11_mouse
Or5w22_mouse
Or5w11_rat
Or5w22b-ps_rat
Or5w22_rat
Or5w8_mouse
Or5w8_rat
Or5w8b_rat
OR5W2_dog
OR5W66P_horse
OR5W31P_cow
OR5W33_cow
OR5W37P_cow
OR5W57_horse
OR5W6_dog
OR5W7_dog
OR5W59_horse
OR5W78P_horse
OR5W2_chimp
OR5W2_human
OR51E1_human
0.2

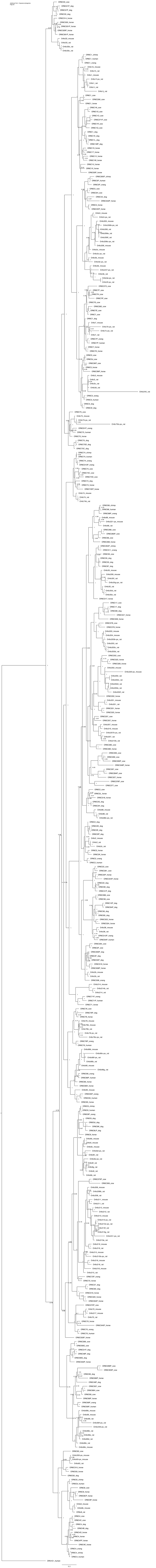

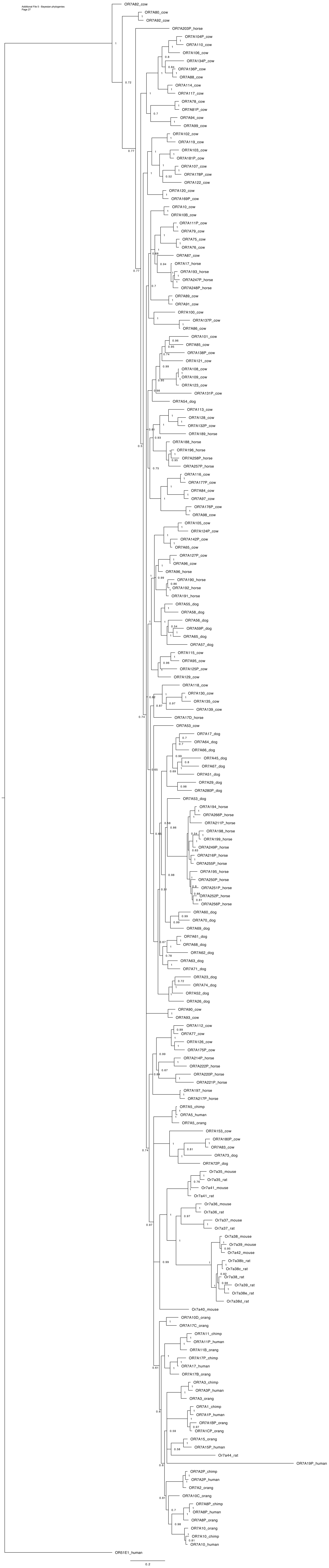
OR7A82_cow
OR7A80_cow
OR7A92_cow
OR7A203P_horse
OR7A104P_cow
OR7A110_cow
OR7A106_cow
OR7A134P_cow
OR7A136P_cow
OR7A88_cow
OR7A114_cow
OR7A117_cow
OR7A78_cow
OR7A81P_cow
OR7A94_cow
OR7A99_cow
OR7A102_cow
OR7A119_cow
OR7A103_cow
OR7A181P_cow
OR7A107_cow
OR7A178P_cow
OR7A122_cow
OR7A120_cow
OR7A169P_cow
OR7A10_cow
OR7A10B_cow
OR7A111P_cow
OR7A79_cow
OR7A75_cow
OR7A76_cow
OR7A87_cow
OR7A17_horse
OR7A193_horse
OR7A247P_horse
OR7A248P_horse
OR7A89_cow
OR7A91_cow
OR7A100_cow
OR7A137P_cow
OR7A86_cow
OR7A101_cow
OR7A85_cow
OR7A138P_cow
OR7A121_cow
OR7A108_cow
OR7A109_cow
OR7A123_cow
OR7A131P_cow
OR7A54_dog
OR7A113_cow
OR7A128_cow
OR7A132P_cow
OR7A189_horse
OR7A188_horse
OR7A196_horse
OR7A258P_horse
OR7A257P_horse
OR7A116_cow
OR7A177P_cow
OR7A84_cow
OR7A97_cow
OR7A176P_cow
OR7A98_cow
OR7A105_cow
OR7A124P_cow
OR7A142P_cow
OR7A65_cow
OR7A127P_cow
OR7A96_cow
OR7A96_horse
OR7A190_horse
OR7A192_horse
OR7A191_horse
OR7A55_dog
OR7A58_dog
OR7A56_dog
OR7A59P_dog
OR7A65_dog
OR7A57_dog
OR7A115_cow
OR7A95_cow
OR7A125P_cow
OR7A129_cow
OR7A118_cow
OR7A130_cow
OR7A135_cow
OR7A139_cow
OR7A17D_horse
OR7A53_cow
OR7A17_dog
OR7A64_dog
OR7A66_dog
OR7A45_dog
OR7A67_dog
OR7A51_dog
OR7A29_dog
OR7A280P_dog
OR7A53_dog
OR7A194_horse
OR7A266P_horse
OR7A211P_horse
OR7A198_horse
OR7A199_horse
OR7A249P_horse
OR7A216P_horse
OR7A255P_horse
OR7A195_horse
OR7A250P_horse
OR7A251P_horse
OR7A252P_horse
OR7A256P_horse
OR7A60_dog
OR7A70_dog
OR7A69_dog
OR7A61_dog
OR7A68_dog
OR7A62_dog
OR7A63_dog
OR7A71_dog
OR7A23_dog
OR7A74_dog
OR7A52_dog
OR7A26_dog
OR7A90_cow
OR7A93_cow
OR7A112_cow
OR7A77_cow
OR7A126_cow
OR7A175P_cow
OR7A214P_horse
OR7A222P_horse
OR7A220P_horse
OR7A221P_horse
OR7A197_horse
OR7A217P_horse
OR7A5_chimp
OR7A5_human
OR7A5_orang
OR7A153_cow
OR7A180P_cow
OR7A83_cow
OR7A73_dog
OR7A72P_dog
Or7a35_mouse
Or7a35_rat
Or7a41_mouse
Or7a41_rat
Or7a36_mouse
Or7a36_rat
Or7a37_mouse
Or7a37_rat
Or7a38_mouse
Or7a39_mouse
Or7a42_mouse
Or7a38b_rat
Or7a38c_rat
Or7a38_rat
Or7a39_rat
Or7a38e_rat
Or7a38d_rat
Or7a40_mouse
OR7A10D_orang
OR7A17C_orang
OR7A11_chimp
OR7A11P_human
OR7A11B_orang
OR7A17P_chimp
OR7A17_human
OR7A17B_orang
OR7A3_chimp
OR7A3P_human
OR7A3_orang
OR7A1_chimp
OR7A1P_human
OR7A1BP_orang
OR7A1CP_orang
OR7A15_orang
OR7A15P_human
Or7a44_rat
OR7A19P_human
OR7A2P_chimp
OR7A2P_human
OR7A2_orang
OR7A10C_orang
OR7A8P_chimp
OR7A8P_human
OR7A8P_orang
OR7A10_orang
OR7A10_chimp
OR7A10_human
OR51E1_human
0.2

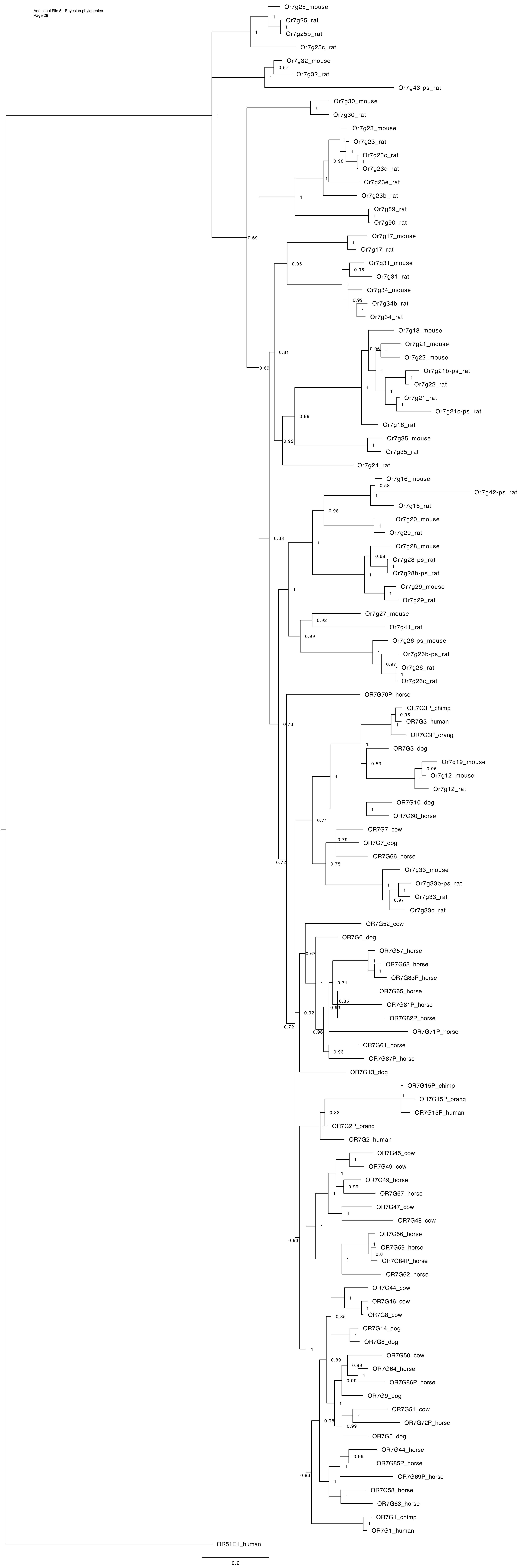
Or7g25_mouse
Or7g25_rat
Or7g25b_rat
Or7g25c_rat
Or7g32_mouse
Or7g32_rat
Or7g43-ps_rat
Or7g30_mouse
Or7g30_rat
Or7g23_mouse
Or7g23_rat
Or7g23c_rat
Or7g23d_rat
Or7g23e_rat
Or7g23b_rat
Or7g89_rat
Or7g90_rat
Or7g17_mouse
Or7g17_rat
Or7g31_mouse
Or7g31_rat
Or7g34_mouse
Or7g34b_rat
Or7g34_rat
Or7g18_mouse
Or7g21_mouse
Or7g22_mouse
Or7g21b-ps_rat
Or7g22_rat
Or7g21_rat
Or7g21c-ps_rat
Or7g18_rat
Or7g35_mouse
Or7g35_rat
Or7g24_rat
Or7g16_mouse
Or7g42-ps_rat
Or7g16_rat
Or7g20_mouse
Or7g20_rat
Or7g28_mouse
Or7g28-ps_rat
Or7g28b-ps_rat
Or7g29_mouse
Or7g29_rat
Or7g27_mouse
Or7g41_rat
Or7g26-ps_mouse
Or7g26b-ps_rat
Or7g26_rat
Or7g26c_rat
OR7G70P_horse
OR7G3P_chimp
OR7G3_human
OR7G3P_orang
OR7G3_dog
Or7g19_mouse
Or7g12_mouse
Or7g12_rat
OR7G10_dog
OR7G60_horse
OR7G7_cow
OR7G7_dog
OR7G66_horse
Or7g33_mouse
Or7g33b-ps_rat
Or7g33_rat
Or7g33c_rat
OR7G52_cow
OR7G6_dog
OR7G57_horse
OR7G68_horse
OR7G83P_horse
OR7G65_horse
OR7G81P_horse
OR7G82P_horse
OR7G71P_horse
OR7G61_horse
OR7G87P_horse
OR7G13_dog
OR7G15P_chimp
OR7G15P_orang
OR7G15P_human
OR7G2P_orang
OR7G2_human
OR7G45_cow
OR7G49_cow
OR7G49_horse
OR7G67_horse
OR7G47_cow
OR7G48_cow
OR7G56_horse
OR7G59_horse
OR7G84P_horse
OR7G62_horse
OR7G44_cow
OR7G46_cow
OR7G8_cow
OR7G14_dog
OR7G8_dog
OR7G50_cow
OR7G64_horse
OR7G86P_horse
OR7G9_dog
OR7G51_cow
OR7G72P_horse
OR7G5_dog
OR7G44_horse
OR7G85P_horse
OR7G69P_horse
OR7G58_horse
OR7G63_horse
OR7G1_chimp
OR7G1_human
OR51E1_human
0.2

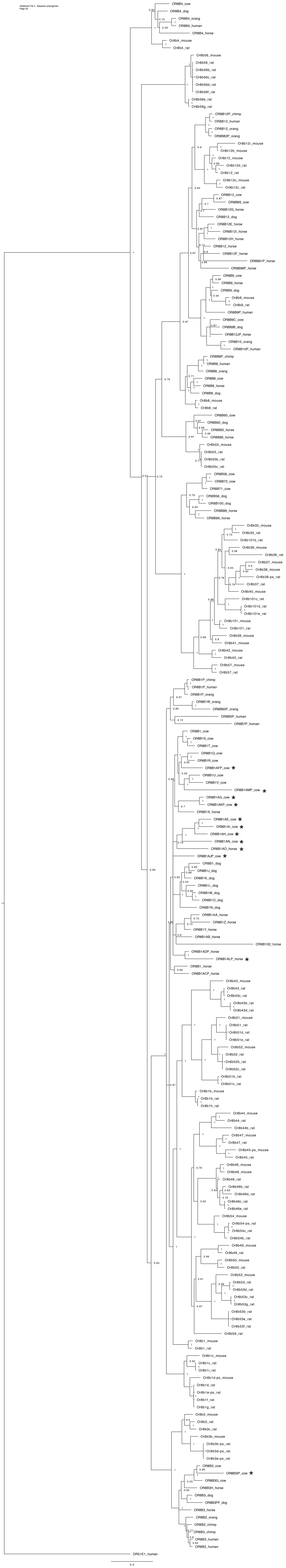
OR8B4_cow
OR8B4_dog
OR8B4_orang
OR8B4_human
OR8B4_horse
Or8b4_mouse
Or8b4_rat
Or8b56_mouse
Or8b56_rat
Or8b56b_rat
Or8b56c_rat
Or8b56d_rat
Or8b56f_rat
Or8b56e_rat
Or8b56g_rat
OR8B12P_chimp
OR8B12_human
OR8B12_orang
OR8B83P_orang
Or8b12i_mouse
Or8b12b_mouse
Or8b12_mouse
Or8b12d_rat
Or8b12_rat
Or8b12c_mouse
Or8b12c_rat
OR8B12_cow
OR8B69_cow
OR8B12G_horse
OR8B12_dog
OR8B12E_horse
OR8B12I_horse
OR8B12H_horse
OR8B12_horse
OR8B12F_horse
OR8B91P_horse
OR8B98P_horse
OR8B9_cow
OR8B9_horse
OR8B9_dog
Or8b9_mouse
Or8b9_rat
OR8B9P_human
OR8B9C_cow
OR8B98_dog
OR8B12JP_horse
OR8B10_orang
OR8B10P_human
OR8B8P_chimp
OR8B8_human
OR8B8_orang
OR8B8_cow
OR8B8_horse
OR8B8_dog
Or8b8_mouse
Or8b8_rat
OR8B60_cow
OR8B60_dog
OR8B60_horse
OR8B86_horse
Or8b55_mouse
Or8b55_rat
Or8b55b_rat
Or8b55c_rat
OR8B58_cow
OR8B72_cow
OR8B71_cow
OR8B58_dog
OR8B100_dog
OR8B88_horse
OR8B89_horse
Or8b35_mouse
Or8b35_rat
Or8b101b_rat
Or8b36_mouse
Or8b36_rat
Or8b37_mouse
Or8b38_mouse
Or8b38-ps_rat
Or8b37_rat
Or8b40_mouse
Or8b101c_rat
Or8b101d_rat
Or8b101e_rat
Or8b101_mouse
Or8b101_rat
Or8b39_mouse
Or8b41_mouse
Or8b42_mouse
Or8b42_rat
Or8b57_mouse
Or8b57_rat
OR8B1P_chimp
OR8B1P_human
OR8B1P_orang
OR8B1W_orang
OR8B85P_orang
OR8B5P_human
OR8B7P_human
OR8B1_cow
OR8B1S_cow
OR8B1T_cow
OR8B1Q_cow
OR8B1R_cow
OR8B1AFP_cow
OR8B1U_cow
OR8B1V_cow
OR8B1AMP_cow
OR8B1AG_cow
OR8B1AKP_cow
OR8B1X_horse
OR8B1AE_cow
OR8B1AI_cow
OR8B1AH_cow
OR8B1AN_cow
OR8B1AO_horse
OR8B1AJP_cow
OR8B1_dog
OR8B1J_dog
OR8B1K_dog
OR8B1L_dog
OR8B1M_dog
OR8B1O_dog
OR8B1N_dog
OR8B1AA_horse
OR8B1Z_horse
OR8B1Y_horse
OR8B1AB_horse
OR8B102_horse
OR8B1ADP_horse
OR8B1ALP_horse
OR8B1_horse
OR8B1ACP_horse
Or8b43_mouse
Or8b43_rat
Or8b43c_rat
Or8b43b_rat
Or8b43d_rat
Or8b51_mouse
Or8b51_rat
Or8b51d_rat
Or8b51e_rat
Or8b52_mouse
Or8b52_rat
Or8b52b_rat
Or8b52c_rat
Or8b51b_rat
Or8b51c_rat
Or8b1b_mouse
Or8b1b_rat
Or8b1h_rat
Or8b44_mouse
Or8b44_rat
Or8b44b_rat
Or8b47_mouse
Or8b47_rat
Or8b45-ps_mouse
Or8b45_rat
Or8b46_mouse
Or8b48_mouse
Or8b46_rat
Or8b46b_rat
Or8b46d_rat
Or8b46c_rat
Or8b46e_rat
Or8b54_mouse
Or8b54-ps_rat
Or8b54c_rat
Or8b54b_rat
Or8b49_mouse
Or8b49_rat
Or8b50_mouse
Or8b50_rat
Or8b53_mouse
Or8b53_rat
Or8b53d_rat
Or8b53c_rat
Or8b53g_rat
Or8b53b_rat
Or8b53e_rat
Or8b53f_rat
Or8b59_rat
Or8b1_mouse
Or8b1_rat
Or8b1c_mouse
Or8b1c_rat
Or8b1i_rat
Or8b1d-ps_mouse
Or8b1d_rat
Or8b1e-ps_rat
Or8b1f_rat
Or8b1g_rat
Or8b3_mouse
Or8b3_rat
Or8b3c_rat
Or8b3b_mouse
Or8b3b-ps_rat
Or8b3d-ps_rat
Or8b3e-ps_rat
OR8B3_cow
OR8B3P_cow
OR8B3G_cow
OR8B3H_horse
OR8B3_dog
OR8B3FP_dog
OR8B3_horse
OR8B2_orang
OR8B2_chimp
OR8B3_chimp
OR8B3_human
OR8B2_human
OR51E1_human
0.2

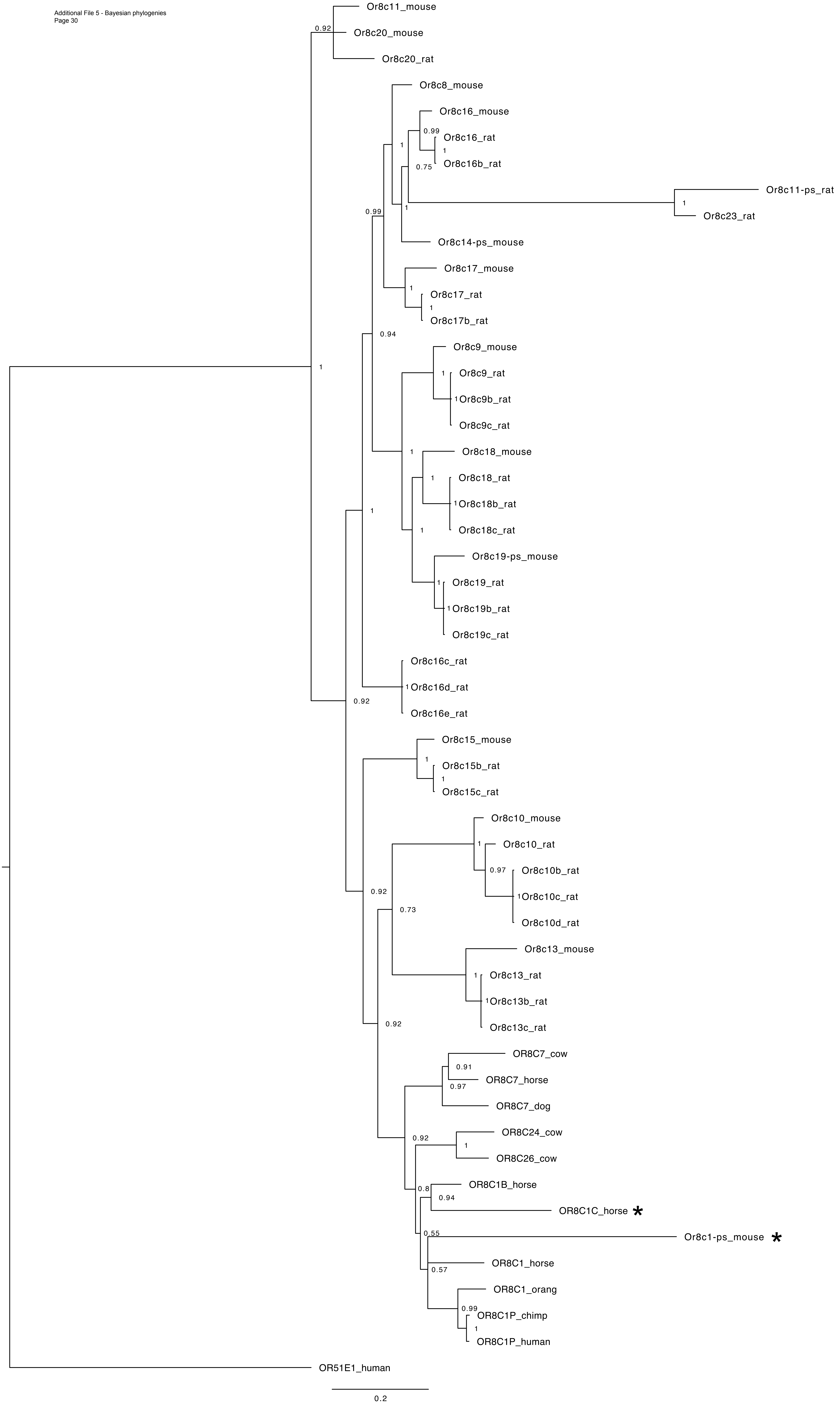
Or8c11_mouse
Or8c20_mouse
Or8c20_rat
Or8c8_mouse
Or8c16_mouse
Or8c16_rat
Or8c16b_rat
Or8c11-ps_rat
Or8c23_rat
Or8c14-ps_mouse
Or8c17_mouse
Or8c17_rat
Or8c17b_rat
Or8c9_mouse
Or8c9_rat
Or8c9b_rat
Or8c9c_rat
Or8c18_mouse
Or8c18_rat
Or8c18b_rat
Or8c18c_rat
Or8c19-ps_mouse
Or8c19_rat
Or8c19b_rat
Or8c19c_rat
Or8c16c_rat
Or8c16d_rat
Or8c16e_rat
Or8c15_mouse
Or8c15b_rat
Or8c15c_rat
Or8c10_mouse
Or8c10_rat
Or8c10b_rat
Or8c10c_rat
Or8c10d_rat
Or8c13_mouse
Or8c13_rat
Or8c13b_rat
Or8c13c_rat
OR8C7_cow
OR8C7_horse
OR8C7_dog
OR8C24_cow
OR8C26_cow
OR8C1B_horse
OR8C1C_horse *
Or8c1-ps_mouse *
OR8C1_horse
OR8C1_orang
OR8C1P_chimp
OR8C1P_human
OR51E1_human
0.2

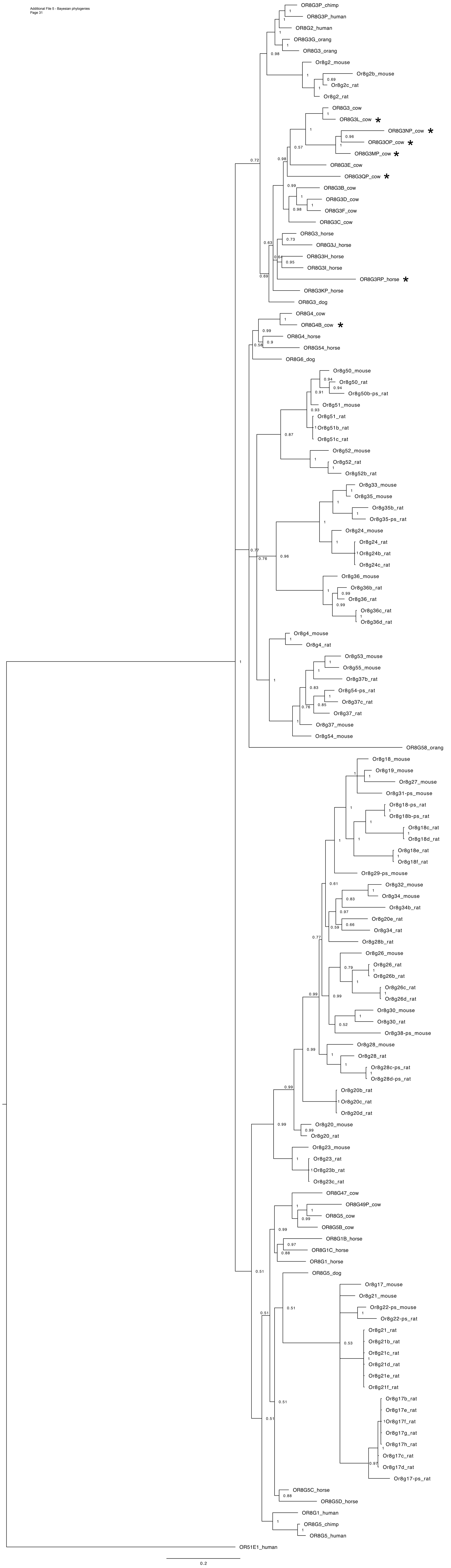
OR8G3P_chimp
OR8G3P_human
OR8G2_human
OR8G3G_orang
OR8G3_orang
Or8g2_mouse
Or8g2b_mouse
Or8g2c_rat
Or8g2_rat
OR8G3_cow
OR8G3L_cow *
OR8G3NP_cow *
OR8G3OP_cow *
OR8G3MP_cow *
OR8G3E_cow
OR8G3QP_cow *
OR8G3B_cow
OR8G3D_cow
OR8G3F_cow
OR8G3C_cow
OR8G3_horse
OR8G3J_horse
OR8G3H_horse
OR8G3I_horse
OR8G3RP_horse *
OR8G3KP_horse
OR8G3_dog
OR8G4_cow
OR8G4B_cow *
OR8G4_horse
OR8G54_horse
OR8G6_dog
Or8g50_mouse
Or8g50_rat
Or8g50b-ps_rat
Or8g51_mouse
Or8g51_rat
Or8g51b_rat
Or8g51c_rat
Or8g52_mouse
Or8g52_rat
Or8g52b_rat
Or8g33_mouse
Or8g35_mouse
Or8g35b_rat
Or8g35-ps_rat
Or8g24_mouse
Or8g24_rat
Or8g24b_rat
Or8g24c_rat
Or8g36_mouse
Or8g36b_rat
Or8g36_rat
Or8g36c_rat
Or8g36d_rat
Or8g4_mouse
Or8g4_rat
Or8g53_mouse
Or8g55_mouse
Or8g37b_rat
Or8g54-ps_rat
Or8g37c_rat
Or8g37_rat
Or8g37_mouse
Or8g54_mouse
OR8G58_orang
Or8g18_mouse
Or8g19_mouse
Or8g27_mouse
Or8g31-ps_mouse
Or8g18-ps_rat
Or8g18b-ps_rat
Or8g18c_rat
Or8g18d_rat
Or8g18e_rat
Or8g18f_rat
Or8g29-ps_mouse
Or8g32_mouse
Or8g34_mouse
Or8g34b_rat
Or8g20e_rat
Or8g34_rat
Or8g28b_rat
Or8g26_mouse
Or8g26_rat
Or8g26b_rat
Or8g26c_rat
Or8g26d_rat
Or8g30_mouse
Or8g30_rat
Or8g38-ps_mouse
Or8g28_mouse
Or8g28_rat
Or8g28c-ps_rat
Or8g28d-ps_rat
Or8g20b_rat
Or8g20c_rat
Or8g20d_rat
Or8g20_mouse
Or8g20_rat
Or8g23_mouse
Or8g23_rat
Or8g23b_rat
Or8g23c_rat
OR8G47_cow
OR8G49P_cow
OR8G5_cow
OR8G5B_cow
OR8G1B_horse
OR8G1C_horse
OR8G1_horse
OR8G5_dog
Or8g17_mouse
Or8g21_mouse
Or8g22-ps_mouse
Or8g22-ps_rat
Or8g21_rat
Or8g21b_rat
Or8g21c_rat
Or8g21d_rat
Or8g21e_rat
Or8g21f_rat
Or8g17b_rat
Or8g17e_rat
Or8g17f_rat
Or8g17g_rat
Or8g17h_rat
Or8g17c_rat
Or8g17d_rat
Or8g17-ps_rat
OR8G5C_horse
OR8G5D_horse
OR8G1_human
OR8G5_chimp
OR8G5_human
OR51E1_human
0.2

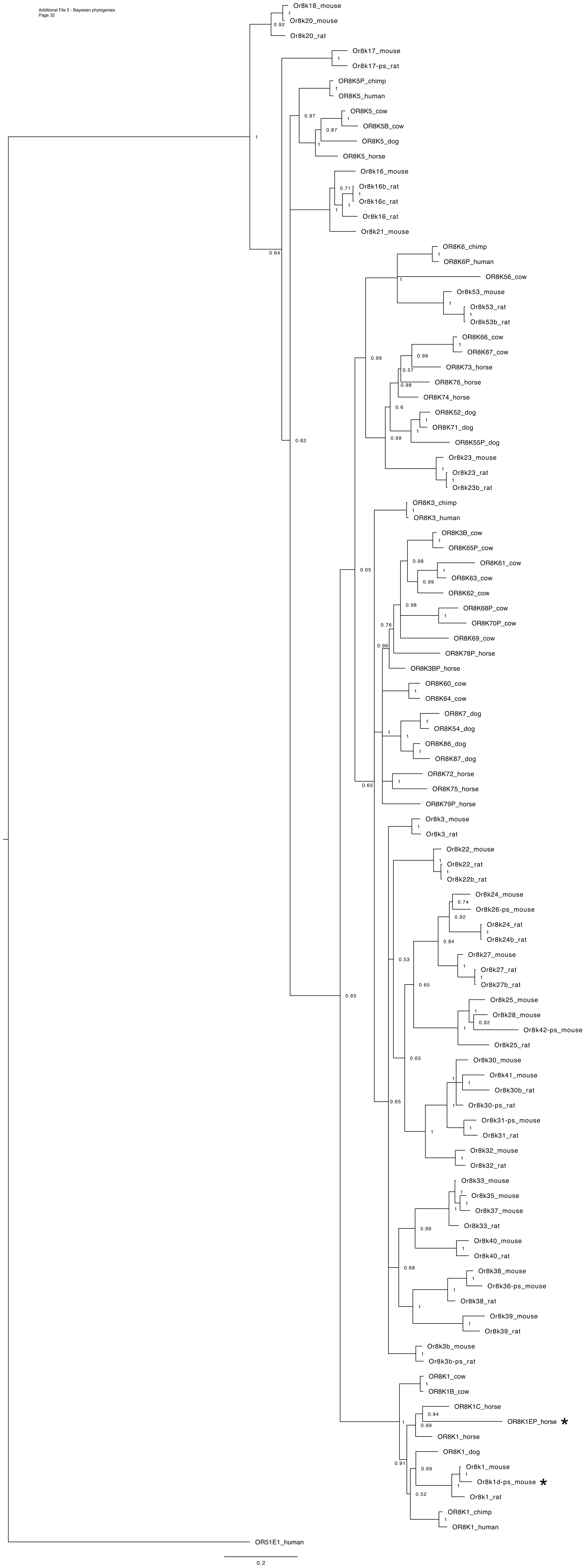
Or8k18_mouse
Or8k20_mouse
Or8k20_rat
Or8k17_mouse
Or8k17-ps_rat
OR8K5P_chimp
OR8K5_human
OR8K5_cow
OR8K5B_cow
OR8K5_dog
OR8K5_horse
Or8k16_mouse
Or8k16b_rat
Or8k16c_rat
Or8k16_rat
Or8k21_mouse
OR8K6_chimp
OR8K6P_human
OR8K56_cow
Or8k53_mouse
Or8k53_rat
Or8k53b_rat
OR8K66_cow
OR8K67_cow
OR8K73_horse
OR8K76_horse
OR8K74_horse
OR8K52_dog
OR8K71_dog
OR8K55P_dog
Or8k23_mouse
Or8k23_rat
Or8k23b_rat
OR8K3_chimp
OR8K3_human
OR8K3B_cow
OR8K65P_cow
OR8K61_cow
OR8K63_cow
OR8K62_cow
OR8K68P_cow
OR8K70P_cow
OR8K69_cow
OR8K78P_horse
OR8K3BP_horse
OR8K60_cow
OR8K64_cow
OR8K7_dog
OR8K54_dog
OR8K86_dog
OR8K87_dog
OR8K72_horse
OR8K75_horse
OR8K79P_horse
Or8k3_mouse
Or8k3_rat
Or8k22_mouse
Or8k22_rat
Or8k22b_rat
Or8k24_mouse
Or8k26-ps_mouse
Or8k24_rat
Or8k24b_rat
Or8k27_mouse
Or8k27_rat
Or8k27b_rat
Or8k25_mouse
Or8k28_mouse
Or8k42-ps_mouse
Or8k25_rat
Or8k30_mouse
Or8k41_mouse
Or8k30b_rat
Or8k30-ps_rat
Or8k31-ps_mouse
Or8k31_rat
Or8k32_mouse
Or8k32_rat
Or8k33_mouse
Or8k35_mouse
Or8k37_mouse
Or8k33_rat
Or8k40_mouse
Or8k40_rat
Or8k38_mouse
Or8k36-ps_mouse
Or8k38_rat
Or8k39_mouse
Or8k39_rat
Or8k3b_mouse
Or8k3b-ps_rat
OR8K1_cow
OR8K1B_cow
OR8K1C_horse
OR8K1EP_horse *
OR8K1_horse
OR8K1_dog
Or8k1_mouse
Or8k1d-ps_mouse *
Or8k1_rat
OR8K1_chimp
OR8K1_human
OR51E1_human
0.2

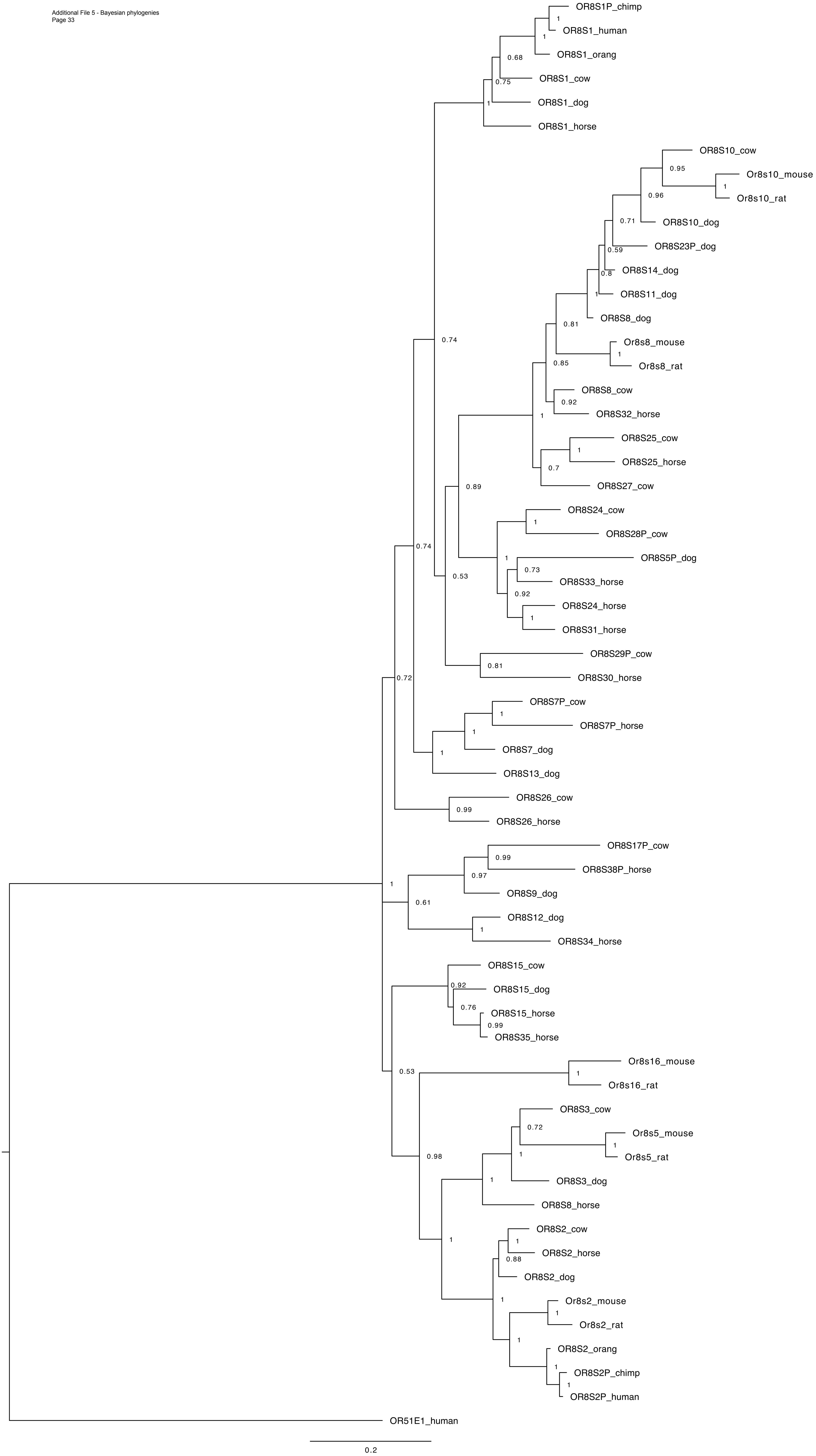
OR8S1P_chimp
OR8S1_human
OR8S1_orang
OR8S1_cow
OR8S1_dog
OR8S1_horse
OR8S10_cow
Or8s10_mouse
Or8s10_rat
OR8S10_dog
OR8S23P_dog
OR8S14_dog
OR8S11_dog
OR8S8_dog
Or8s8_mouse
Or8s8_rat
OR8S8_cow
OR8S32_horse
OR8S25_cow
OR8S25_horse
OR8S27_cow
OR8S24_cow
OR8S28P_cow
OR8S5P_dog
OR8S33_horse
OR8S24_horse
OR8S31_horse
OR8S29P_cow
OR8S30_horse
OR8S7P_cow
OR8S7P_horse
OR8S7_dog
OR8S13_dog
OR8S26_cow
OR8S26_horse
OR8S17P_cow
OR8S38P_horse
OR8S9_dog
OR8S12_dog
OR8S34_horse
OR8S15_cow
OR8S15_dog
OR8S15_horse
OR8S35_horse
Or8s16_mouse
Or8s16_rat
OR8S3_cow
Or8s5_mouse
Or8s5_rat
OR8S3_dog
OR8S8_horse
OR8S2_cow
OR8S2_horse
OR8S2_dog
Or8s2_mouse
Or8s2_rat
OR8S2_orang
OR8S2P_chimp
OR8S2P_human
OR51E1_human
0.2

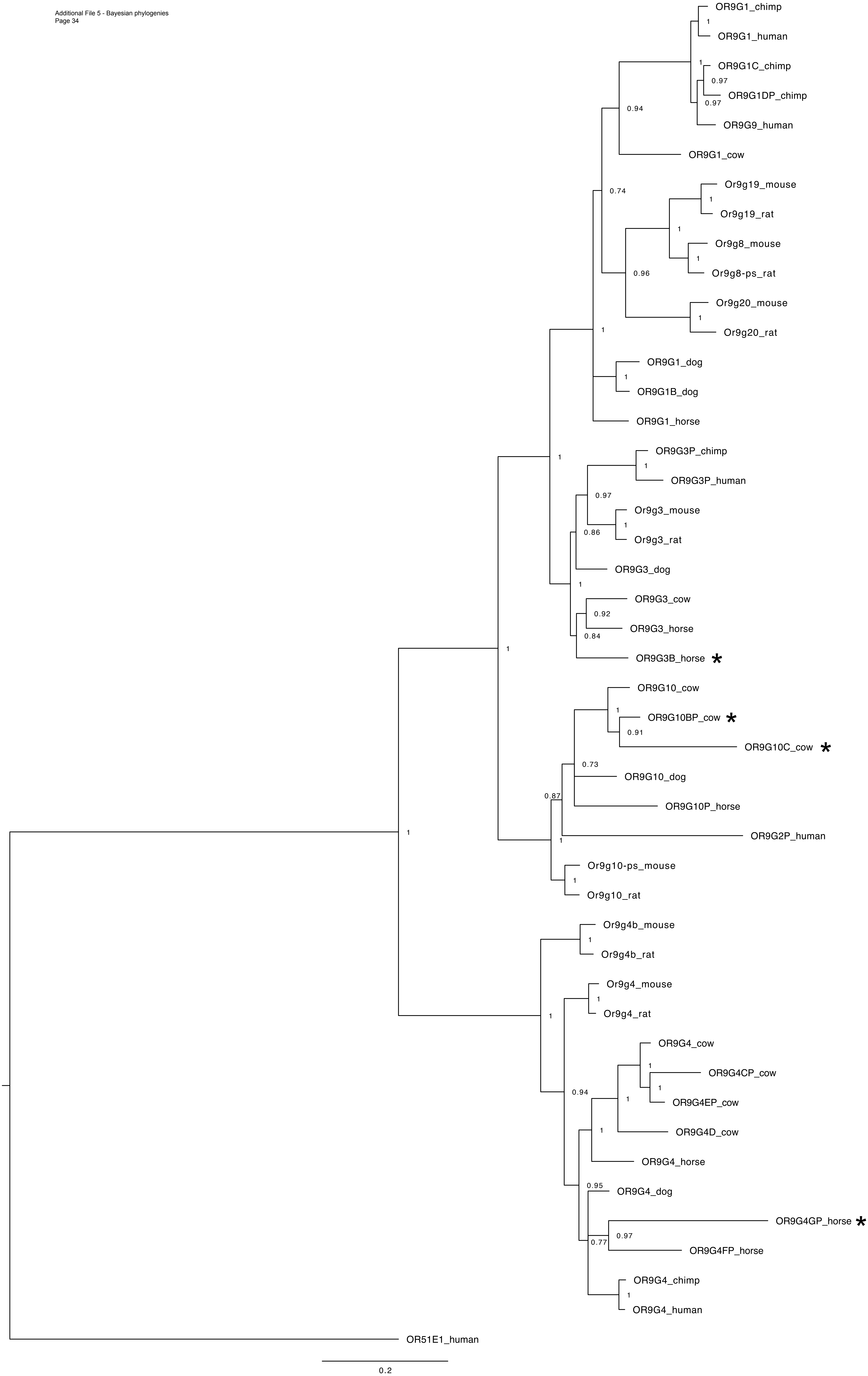
OR9G1_chimp
1
OR9G1_human
1
OR9G1C_chimp
0.97
OR9G1DP_chimp
0.97
0.94
OR9G9_human
OR9G1_cow
Or9g19_mouse
0.74
1
Or9g19_rat
1
Or9g8_mouse
1
0.96
Or9g8-ps_rat
Or9g20_mouse
1
1
Or9g20_rat
OR9G1_dog
1
OR9G1B_dog
OR9G1_horse
OR9G3P_chimp
1
1
OR9G3P_human
0.97
Or9g3_mouse
1
0.86
Or9g3_rat
OR9G3_dog
1
OR9G3_cow
0.92
OR9G3_horse
0.84
1
OR9G3B_horse *
OR9G10_cow
1
OR9G10BP_cow *
0.91
OR9G10C_cow *
0.73
OR9G10_dog
0.87
OR9G10P_horse
1
OR9G2P_human
1
Or9g10-ps_mouse
1
Or9g10_rat
Or9g4b_mouse
1
Or9g4b_rat
Or9g4_mouse
1
1
Or9g4_rat
OR9G4_cow
1
OR9G4CP_cow
1
0.94
1
OR9G4EP_cow
1
OR9G4D_cow
OR9G4_horse
0.95
OR9G4_dog
OR9G4GP_horse *
0.97
0.77
OR9G4FP_horse
OR9G4_chimp
1
OR9G4_human
OR51E1_human
0.2

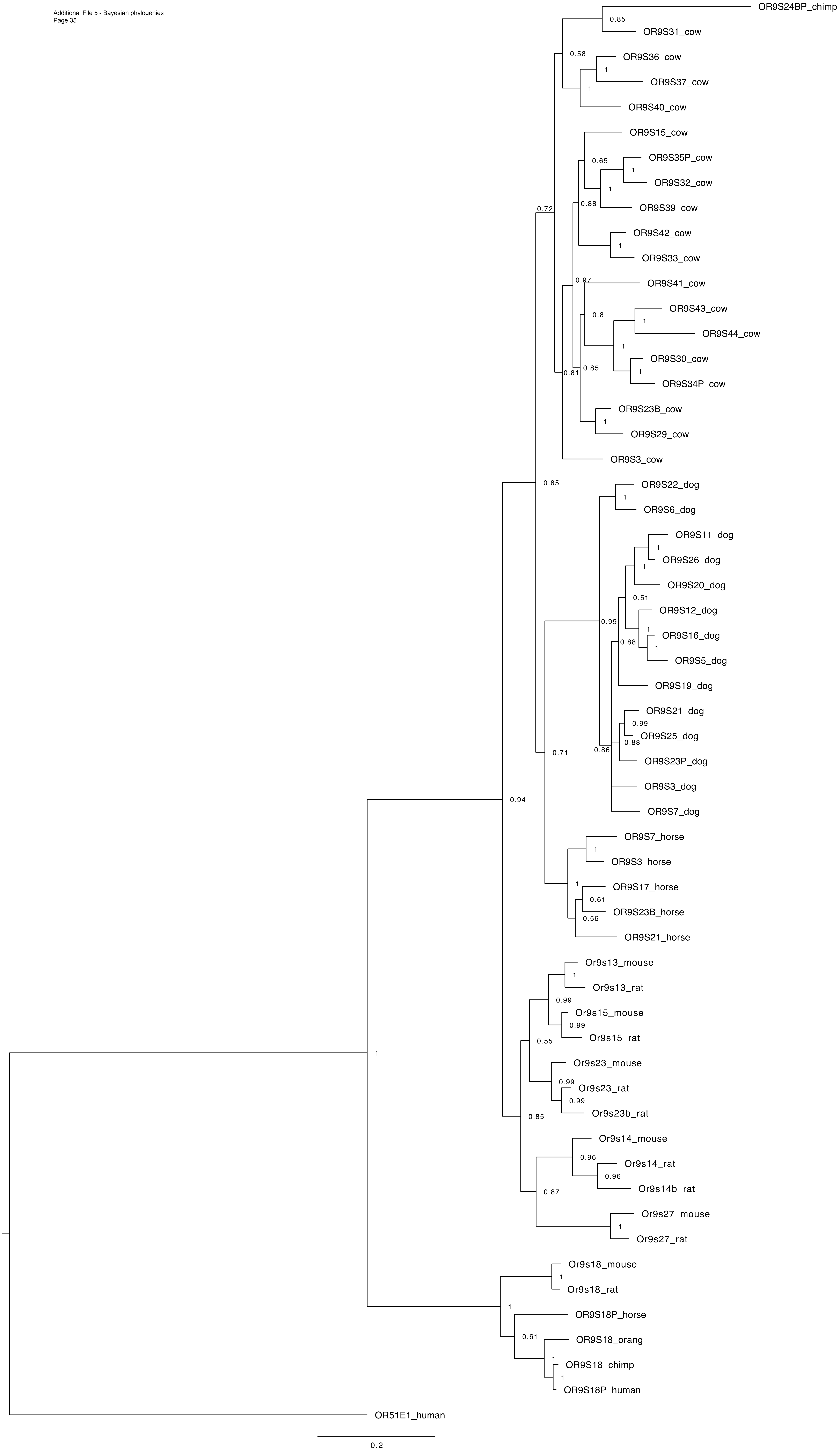
OR9S24BP_chimp
OR9S31_cow
OR9S36_cow
OR9S37_cow
OR9S40_cow
OR9S15_cow
OR9S35P_cow
OR9S32_cow
OR9S39_cow
OR9S42_cow
OR9S33_cow
OR9S41_cow
OR9S43_cow
OR9S44_cow
OR9S30_cow
OR9S34P_cow
OR9S23B_cow
OR9S29_cow
OR9S3_cow
OR9S22_dog
OR9S6_dog
OR9S11_dog
OR9S26_dog
OR9S20_dog
OR9S12_dog
OR9S16_dog
OR9S5_dog
OR9S19_dog
OR9S21_dog
OR9S25_dog
OR9S23P_dog
OR9S3_dog
OR9S7_dog
OR9S7_horse
OR9S3_horse
OR9S17_horse
OR9S23B_horse
OR9S21_horse
Or9s13_mouse
Or9s13_rat
Or9s15_mouse
Or9s15_rat
Or9s23_mouse
Or9s23_rat
Or9s23b_rat
Or9s14_mouse
Or9s14_rat
Or9s14b_rat
Or9s27_mouse
Or9s27_rat
Or9s18_mouse
Or9s18_rat
OR9S18P_horse
OR9S18_orang
OR9S18_chimp
OR9S18P_human
OR51E1_human
0.2

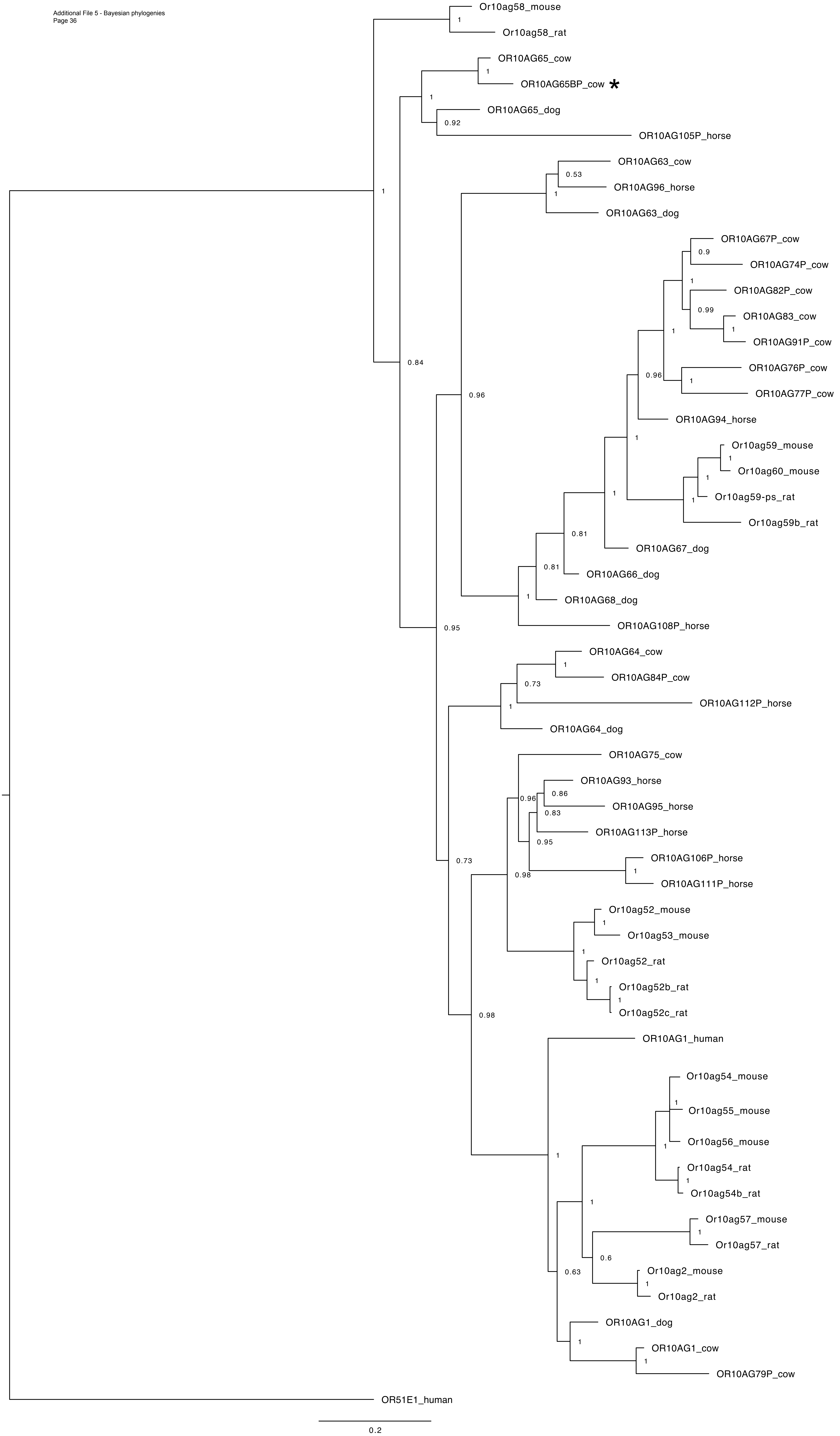
Or10ag58_mouse
Or10ag58_rat
OR10AG65_cow
OR10AG65BP_cow *
OR10AG65_dog
OR10AG105P_horse
OR10AG63_cow
OR10AG96_horse
OR10AG63_dog
OR10AG67P_cow
OR10AG74P_cow
OR10AG82P_cow
OR10AG83_cow
OR10AG91P_cow
OR10AG76P_cow
OR10AG77P_cow
OR10AG94_horse
Or10ag59_mouse
Or10ag60_mouse
Or10ag59-ps_rat
Or10ag59b_rat
OR10AG67_dog
OR10AG66_dog
OR10AG68_dog
OR10AG108P_horse
OR10AG64_cow
OR10AG84P_cow
OR10AG112P_horse
OR10AG64_dog
OR10AG75_cow
OR10AG93_horse
OR10AG95_horse
OR10AG113P_horse
OR10AG106P_horse
OR10AG111P_horse
Or10ag52_mouse
Or10ag53_mouse
Or10ag52_rat
Or10ag52b_rat
Or10ag52c_rat
OR10AG1_human
Or10ag54_mouse
Or10ag55_mouse
Or10ag56_mouse
Or10ag54_rat
Or10ag54b_rat
Or10ag57_mouse
Or10ag57_rat
Or10ag2_mouse
Or10ag2_rat
OR10AG1_dog
OR10AG1_cow
OR10AG79P_cow
OR51E1_human
0.2

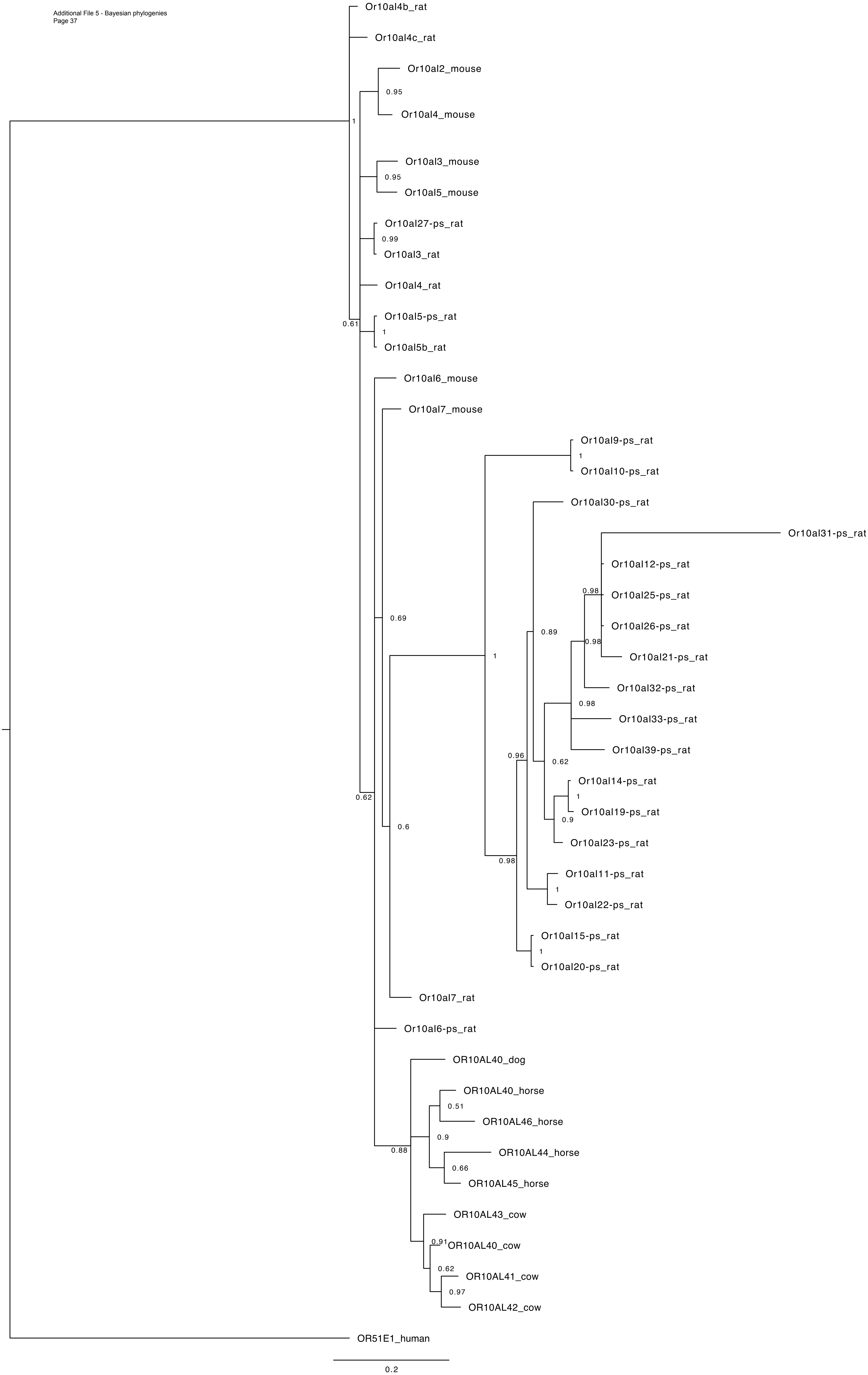
Or10al4b_rat
Or10al4c_rat
Or10al2_mouse
0.95
Or10al4_mouse
1
Or10al3_mouse
0.95
Or10al5_mouse
Or10al27-ps_rat
0.99
Or10al3_rat
Or10al4_rat
0.61
Or10al5-ps_rat
1
Or10al5b_rat
Or10al6_mouse
Or10al7_mouse
Or10al9-ps_rat
1
Or10al10-ps_rat
Or10al30-ps_rat
Or10al31-ps_rat
Or10al12-ps_rat
0.98
Or10al25-ps_rat
0.69
Or10al26-ps_rat
0.89
0.98
Or10al21-ps_rat
1
Or10al32-ps_rat
0.98
Or10al33-ps_rat
Or10al39-ps_rat
0.96
0.62
Or10al14-ps_rat
1
0.62
Or10al19-ps_rat
0.6
0.9
Or10al23-ps_rat
0.98
Or10al11-ps_rat
1
Or10al22-ps_rat
Or10al15-ps_rat
1
Or10al20-ps_rat
Or10al7_rat
Or10al6-ps_rat
OR10AL40_dog
OR10AL40_horse
0.51
OR10AL46_horse
0.9
OR10AL44_horse
0.88
0.66
OR10AL45_horse
OR10AL43_cow
0.91
OR10AL40_cow
0.62
OR10AL41_cow
0.97
OR10AL42_cow
OR51E1_human
0.2

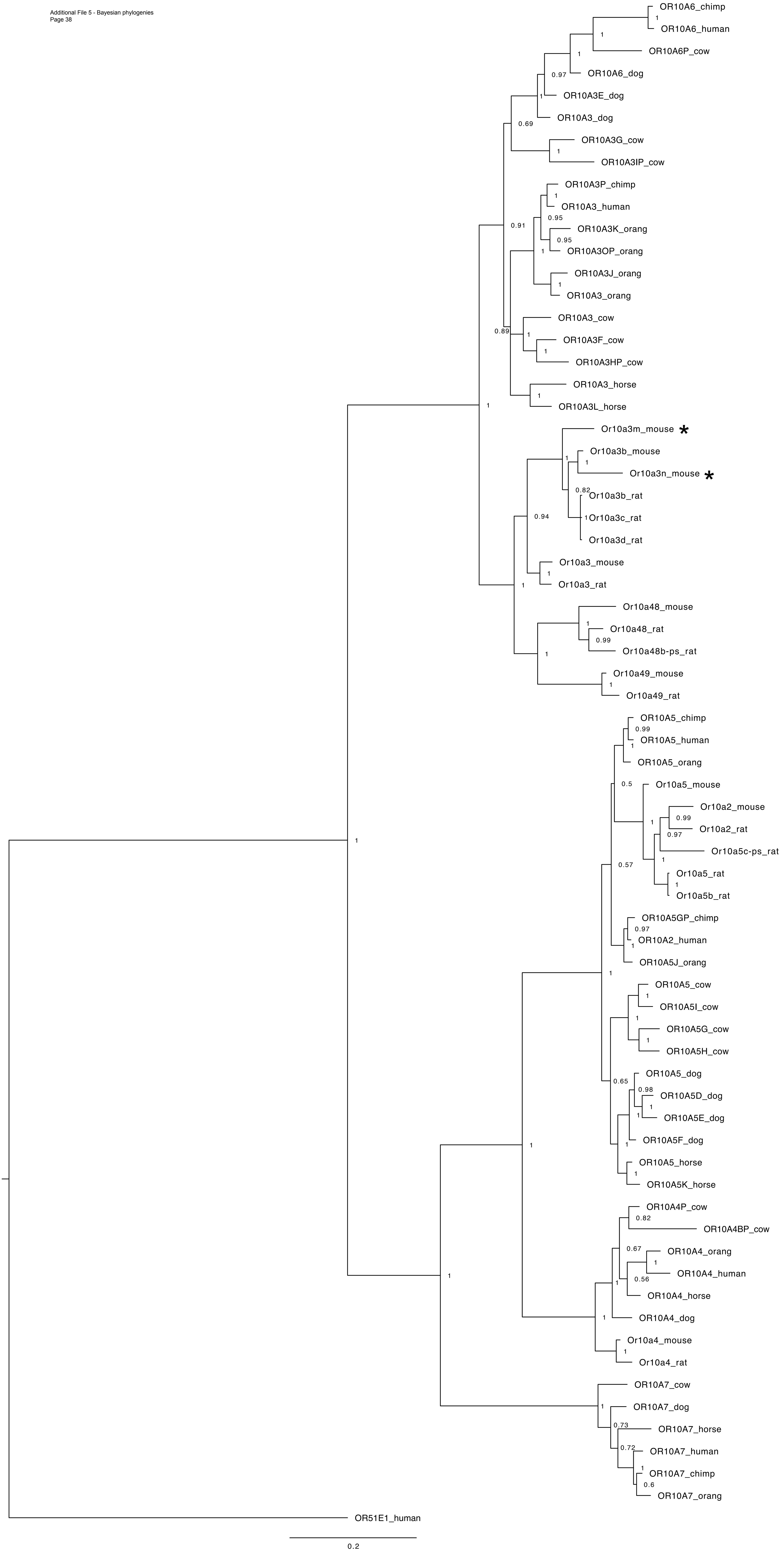
OR10A6_chimp
OR10A6_human
OR10A6P_cow
OR10A6_dog
OR10A3E_dog
OR10A3_dog
OR10A3G_cow
OR10A3IP_cow
OR10A3P_chimp
OR10A3_human
OR10A3K_orang
OR10A3OP_orang
OR10A3J_orang
OR10A3_orang
OR10A3_cow
OR10A3F_cow
OR10A3HP_cow
OR10A3_horse
OR10A3L_horse
Or10a3m_mouse *
Or10a3b_mouse
Or10a3n_mouse *
Or10a3b_rat
Or10a3c_rat
Or10a3d_rat
Or10a3_mouse
Or10a3_rat
Or10a48_mouse
Or10a48_rat
Or10a48b-ps_rat
Or10a49_mouse
Or10a49_rat
OR10A5_chimp
OR10A5_human
OR10A5_orang
Or10a5_mouse
Or10a2_mouse
Or10a2_rat
Or10a5c-ps_rat
Or10a5_rat
Or10a5b_rat
OR10A5GP_chimp
OR10A2_human
OR10A5J_orang
OR10A5_cow
OR10A5I_cow
OR10A5G_cow
OR10A5H_cow
OR10A5_dog
OR10A5D_dog
OR10A5E_dog
OR10A5F_dog
OR10A5_horse
OR10A5K_horse
OR10A4P_cow
OR10A4BP_cow
OR10A4_orang
OR10A4_human
OR10A4_horse
OR10A4_dog
Or10a4_mouse
Or10a4_rat
OR10A7_cow
OR10A7_dog
OR10A7_horse
OR10A7_human
OR10A7_chimp
OR10A7_orang
OR51E1_human
0.2

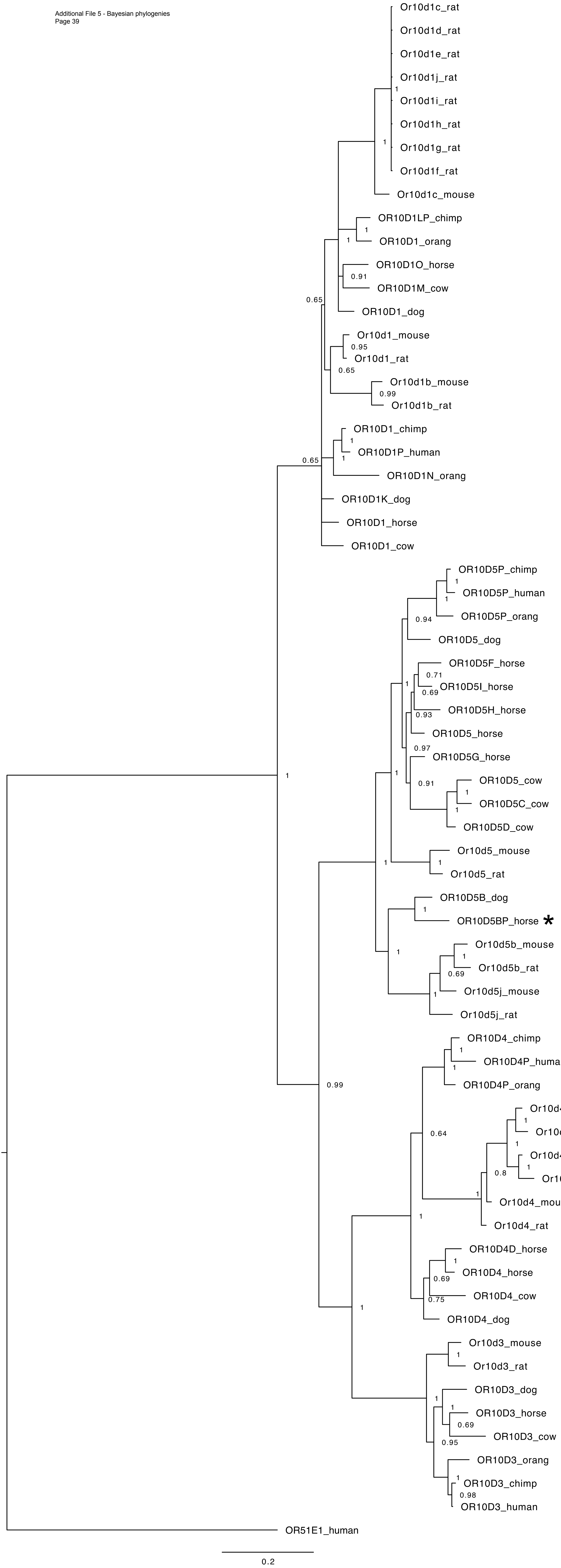
Or10d1c_rat
Or10d1d_rat
Or10d1e_rat
Or10d1j_rat
Or10d1i_rat
Or10d1h_rat
Or10d1g_rat
Or10d1f_rat
Or10d1c_mouse
OR10D1LP_chimp
OR10D1_orang
OR10D1O_horse
OR10D1M_cow
OR10D1_dog
Or10d1_mouse
Or10d1_rat
Or10d1b_mouse
Or10d1b_rat
OR10D1_chimp
OR10D1P_human
OR10D1N_orang
OR10D1K_dog
OR10D1_horse
OR10D1_cow
OR10D5P_chimp
OR10D5P_human
OR10D5P_orang
OR10D5_dog
OR10D5F_horse
OR10D5I_horse
OR10D5H_horse
OR10D5_horse
OR10D5G_horse
OR10D5_cow
OR10D5C_cow
OR10D5D_cow
Or10d5_mouse
Or10d5_rat
OR10D5B_dog
OR10D5BP_horse *
Or10d5b_mouse
Or10d5b_rat
Or10d5j_mouse
Or10d5j_rat
OR10D4_chimp
OR10D4P_human
OR10D4P_orang
Or10d4b_mouse
Or10d4b_rat
Or10d4c_mouse
Or10d4c_rat
Or10d4_mouse
Or10d4_rat
OR10D4D_horse
OR10D4_horse
OR10D4_cow
OR10D4_dog
Or10d3_mouse
Or10d3_rat
OR10D3_dog
OR10D3_horse
OR10D3_cow
OR10D3_orang
OR10D3_chimp
OR10D3_human
OR51E1_human
0.2

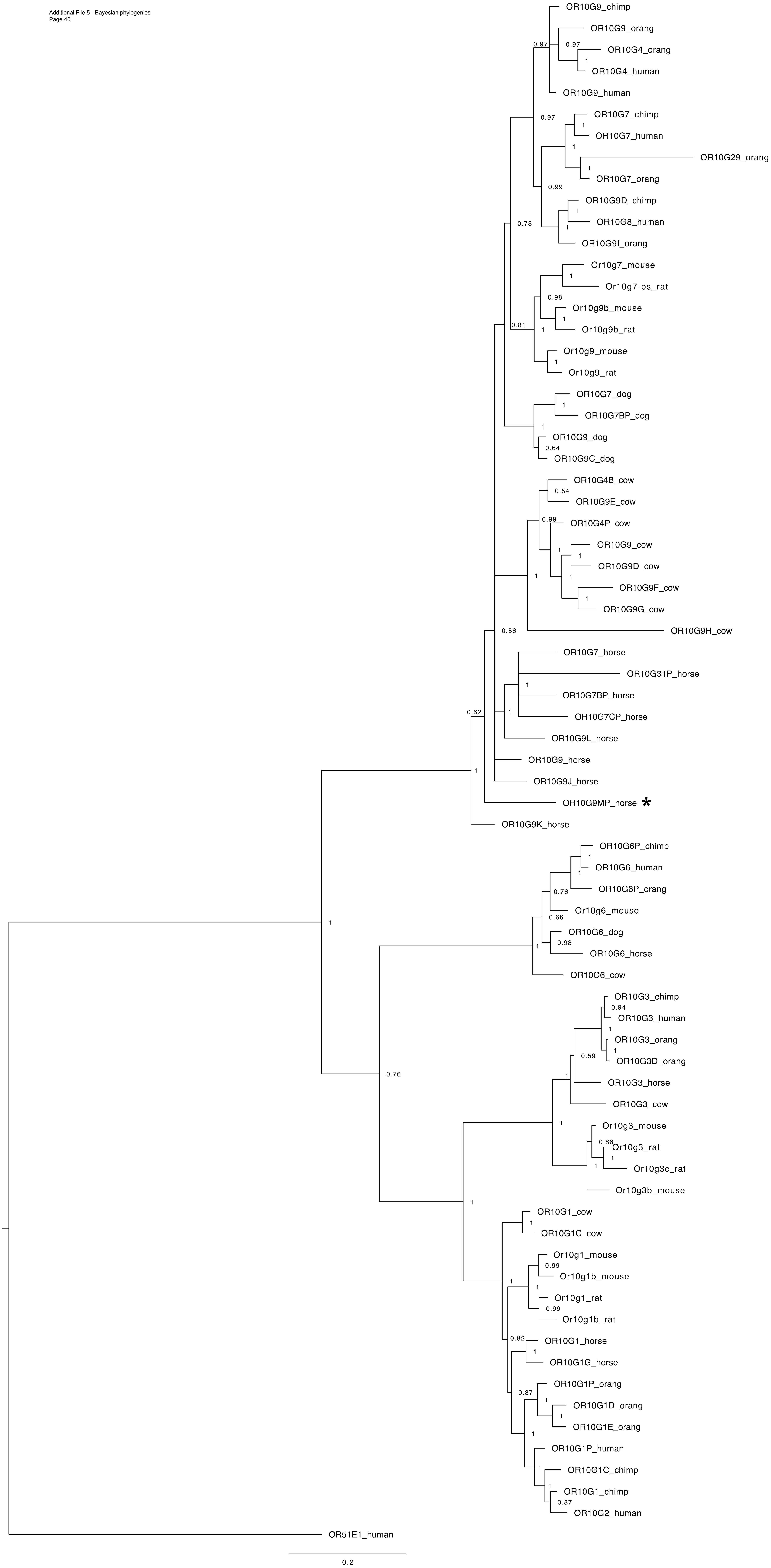
OR10G9_chimp
OR10G9_orang
OR10G4_orang
OR10G4_human
OR10G9_human
OR10G7_chimp
OR10G7_human
OR10G29_orang
OR10G7_orang
OR10G9D_chimp
OR10G8_human
OR10G9I_orang
Or10g7_mouse
Or10g7-ps_rat
Or10g9b_mouse
Or10g9b_rat
Or10g9_mouse
Or10g9_rat
OR10G7_dog
OR10G7BP_dog
OR10G9_dog
OR10G9C_dog
OR10G4B_cow
OR10G9E_cow
OR10G4P_cow
OR10G9_cow
OR10G9D_cow
OR10G9F_cow
OR10G9G_cow
OR10G9H_cow
OR10G7_horse
OR10G31P_horse
OR10G7BP_horse
OR10G7CP_horse
OR10G9L_horse
OR10G9_horse
OR10G9J_horse
OR10G9MP_horse *
OR10G9K_horse
OR10G6P_chimp
OR10G6_human
OR10G6P_orang
Or10g6_mouse
OR10G6_dog
OR10G6_horse
OR10G6_cow
OR10G3_chimp
OR10G3_human
OR10G3_orang
OR10G3D_orang
OR10G3_horse
OR10G3_cow
Or10g3_mouse
Or10g3_rat
Or10g3c_rat
Or10g3b_mouse
OR10G1_cow
OR10G1C_cow
Or10g1_mouse
Or10g1b_mouse
Or10g1_rat
Or10g1b_rat
OR10G1_horse
OR10G1G_horse
OR10G1P_orang
OR10G1D_orang
OR10G1E_orang
OR10G1P_human
OR10G1C_chimp
OR10G1_chimp
OR10G2_human
OR51E1_human
0.2

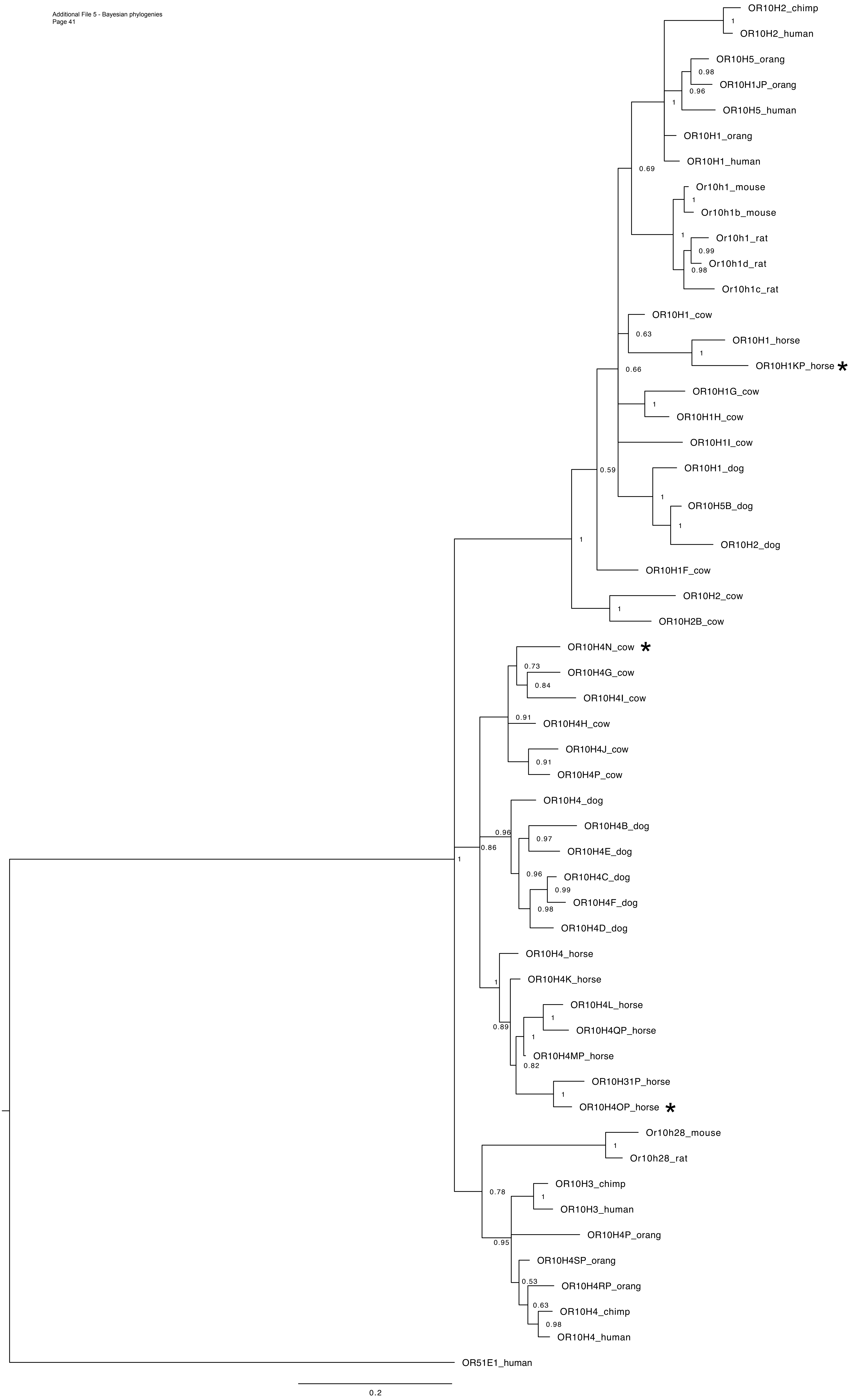
OR10H2_chimp
1
OR10H2_human
OR10H5_orang
0.98
OR10H1JP_orang
0.96
1
OR10H5_human
OR10H1_orang
OR10H1_human
0.69
Or10h1_mouse
1
Or10h1b_mouse
1
Or10h1_rat
0.99
Or10h1d_rat
0.98
Or10h1c_rat
OR10H1_cow
0.63
OR10H1_horse
1
OR10H1KP_horse *
0.66
OR10H1G_cow
1
OR10H1H_cow
OR10H1I_cow
OR10H1_dog
0.59
1
OR10H5B_dog
1
OR10H2_dog
1
OR10H1F_cow
OR10H2_cow
1
OR10H2B_cow
OR10H4N_cow *
0.73
OR10H4G_cow
0.84
OR10H4I_cow
0.91
OR10H4H_cow
OR10H4J_cow
0.91
OR10H4P_cow
OR10H4_dog
OR10H4B_dog
0.96
0.97
0.86
OR10H4E_dog
1
0.96
OR10H4C_dog
0.99
OR10H4F_dog
0.98
OR10H4D_dog
OR10H4_horse
OR10H4K_horse
1
OR10H4L_horse
1
0.89
OR10H4QP_horse
1
OR10H4MP_horse
0.82
OR10H31P_horse
1
OR10H4OP_horse *
Or10h28_mouse
1
Or10h28_rat
OR10H3_chimp
0.78
1
OR10H3_human
OR10H4P_orang
0.95
OR10H4SP_orang
0.53
OR10H4RP_orang
0.63
OR10H4_chimp
0.98
OR10H4_human
OR51E1_human
0.2

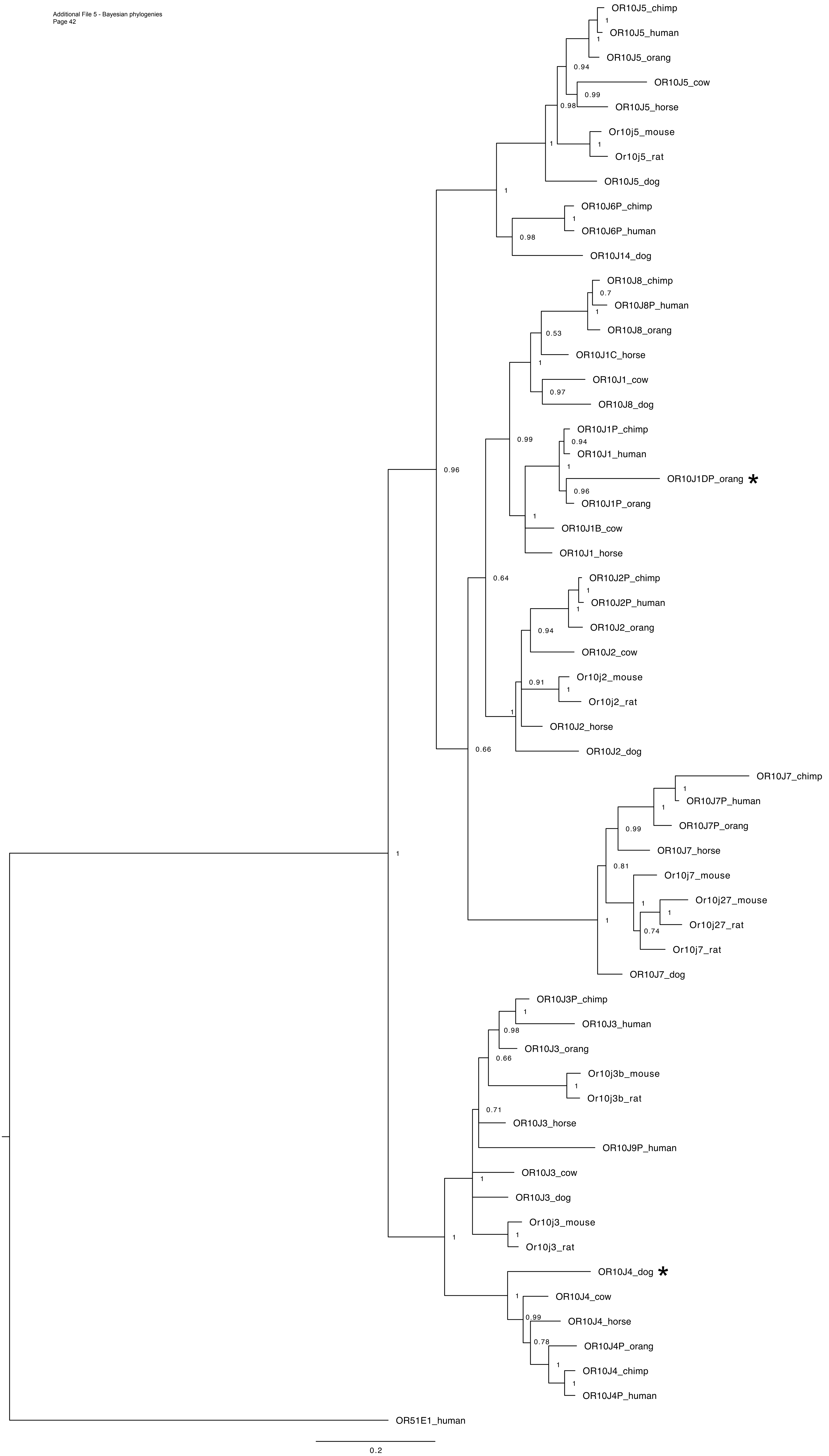
OR10J5_chimp
OR10J5_human
OR10J5_orang
OR10J5_cow
OR10J5_horse
Or10j5_mouse
Or10j5_rat
OR10J5_dog
OR10J6P_chimp
OR10J6P_human
OR10J14_dog
OR10J8_chimp
OR10J8P_human
OR10J8_orang
OR10J1C_horse
OR10J1_cow
OR10J8_dog
OR10J1P_chimp
OR10J1_human
OR10J1DP_orang *
OR10J1P_orang
OR10J1B_cow
OR10J1_horse
OR10J2P_chimp
OR10J2P_human
OR10J2_orang
OR10J2_cow
Or10j2_mouse
Or10j2_rat
OR10J2_horse
OR10J2_dog
OR10J7_chimp
OR10J7P_human
OR10J7P_orang
OR10J7_horse
Or10j7_mouse
Or10j27_mouse
Or10j27_rat
Or10j7_rat
OR10J7_dog
OR10J3P_chimp
OR10J3_human
OR10J3_orang
Or10j3b_mouse
Or10j3b_rat
OR10J3_horse
OR10J9P_human
OR10J3_cow
OR10J3_dog
Or10j3_mouse
Or10j3_rat
OR10J4_dog *
OR10J4_cow
OR10J4_horse
OR10J4P_orang
OR10J4_chimp
OR10J4P_human
OR51E1_human
1
1
0.94
0.99
0.98
1
1
1
0.98
0.7
1
0.53
1
0.97
0.99
0.94
1
0.96
1
0.96
0.64
1
1
0.94
0.91
1
1
0.66
1
1
0.99
0.81
1
1
0.74
1
1
1
0.98
0.66
1
0.71
1
1
1
1
0.99
0.78
1
1
0.2

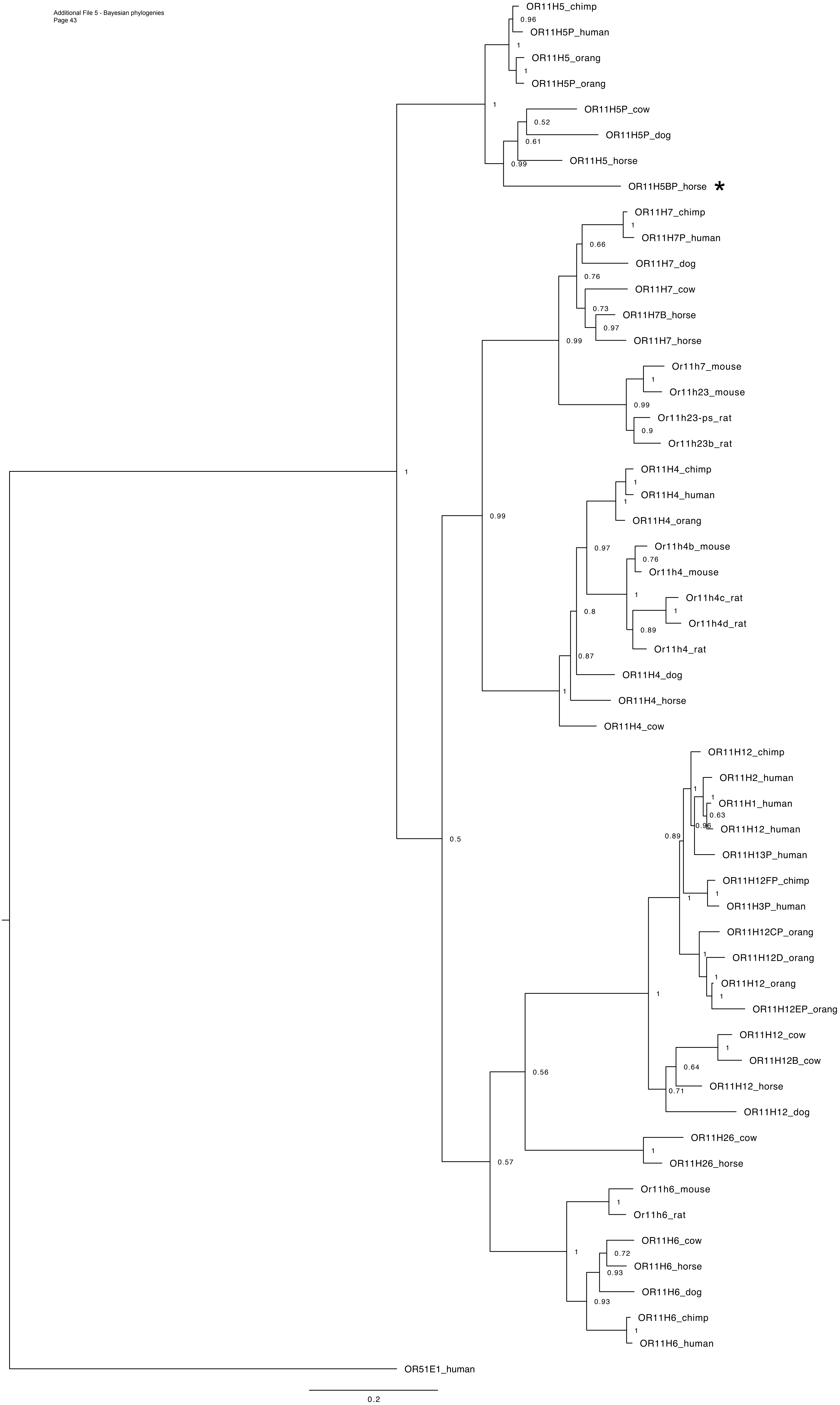
OR11H5_chimp
OR11H5P_human
OR11H5_orang
OR11H5P_orang
OR11H5P_cow
OR11H5P_dog
OR11H5_horse
OR11H5BP_horse *
OR11H7_chimp
OR11H7P_human
OR11H7_dog
OR11H7_cow
OR11H7B_horse
OR11H7_horse
Or11h7_mouse
Or11h23_mouse
Or11h23-ps_rat
Or11h23b_rat
OR11H4_chimp
OR11H4_human
OR11H4_orang
Or11h4b_mouse
Or11h4_mouse
Or11h4c_rat
Or11h4d_rat
Or11h4_rat
OR11H4_dog
OR11H4_horse
OR11H4_cow
OR11H12_chimp
OR11H2_human
OR11H1_human
OR11H12_human
OR11H13P_human
OR11H12FP_chimp
OR11H3P_human
OR11H12CP_orang
OR11H12D_orang
OR11H12_orang
OR11H12EP_orang
OR11H12_cow
OR11H12B_cow
OR11H12_horse
OR11H12_dog
OR11H26_cow
OR11H26_horse
Or11h6_mouse
Or11h6_rat
OR11H6_cow
OR11H6_horse
OR11H6_dog
OR11H6_chimp
OR11H6_human
OR51E1_human
0.2

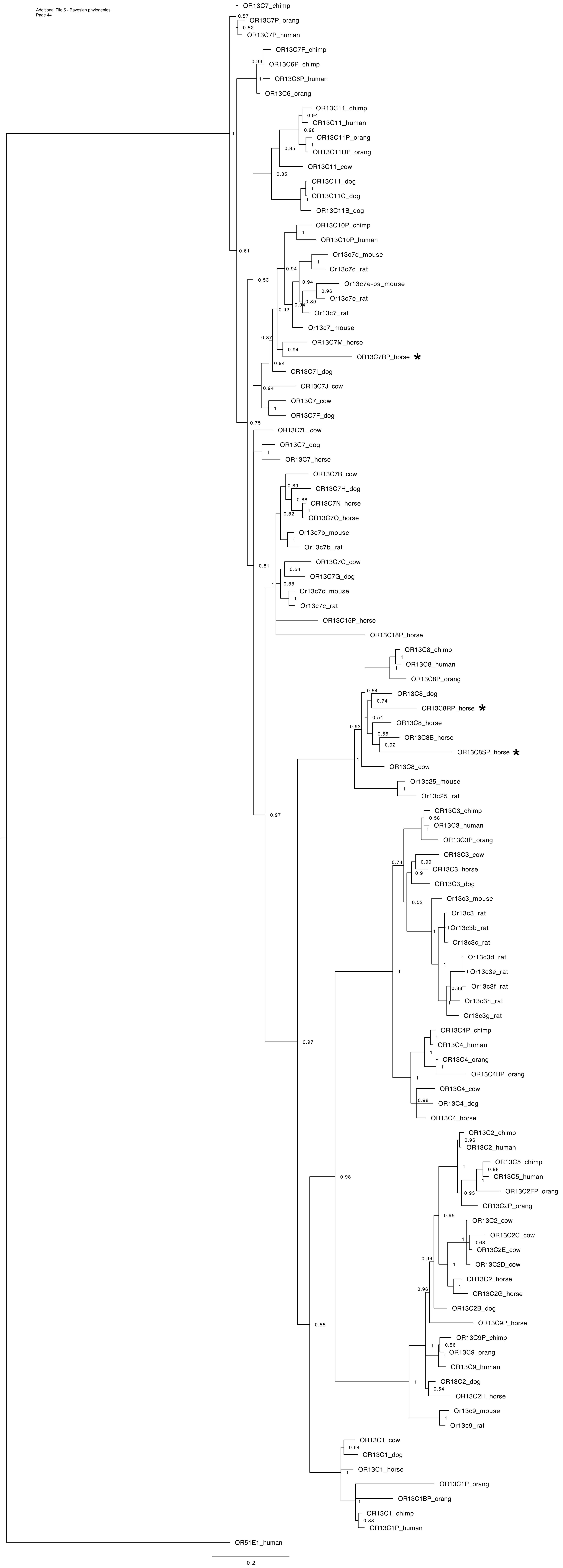
OR13C7_chimp
OR13C7P_orang
OR13C7P_human
OR13C7F_chimp
OR13C6P_chimp
OR13C6P_human
OR13C6_orang
OR13C11_chimp
OR13C11_human
OR13C11P_orang
OR13C11DP_orang
OR13C11_cow
OR13C11_dog
OR13C11C_dog
OR13C11B_dog
OR13C10P_chimp
OR13C10P_human
Or13c7d_mouse
Or13c7d_rat
Or13c7e-ps_mouse
Or13c7e_rat
Or13c7_rat
Or13c7_mouse
OR13C7M_horse
OR13C7RP_horse *
OR13C7I_dog
OR13C7J_cow
OR13C7_cow
OR13C7F_dog
OR13C7L_cow
OR13C7_dog
OR13C7_horse
OR13C7B_cow
OR13C7H_dog
OR13C7N_horse
OR13C7O_horse
Or13c7b_mouse
Or13c7b_rat
OR13C7C_cow
OR13C7G_dog
Or13c7c_mouse
Or13c7c_rat
OR13C15P_horse
OR13C18P_horse
OR13C8_chimp
OR13C8_human
OR13C8P_orang
OR13C8_dog
OR13C8RP_horse *
OR13C8_horse
OR13C8B_horse
OR13C8SP_horse *
OR13C8_cow
Or13c25_mouse
Or13c25_rat
OR13C3_chimp
OR13C3_human
OR13C3P_orang
OR13C3_cow
OR13C3_horse
OR13C3_dog
Or13c3_mouse
Or13c3_rat
Or13c3b_rat
Or13c3c_rat
Or13c3d_rat
Or13c3e_rat
Or13c3f_rat
Or13c3h_rat
Or13c3g_rat
OR13C4P_chimp
OR13C4_human
OR13C4_orang
OR13C4BP_orang
OR13C4_cow
OR13C4_dog
OR13C4_horse
OR13C2_chimp
OR13C2_human
OR13C5_chimp
OR13C5_human
OR13C2FP_orang
OR13C2P_orang
OR13C2_cow
OR13C2C_cow
OR13C2E_cow
OR13C2D_cow
OR13C2_horse
OR13C2G_horse
OR13C2B_dog
OR13C9P_horse
OR13C9P_chimp
OR13C9_orang
OR13C9_human
OR13C2_dog
OR13C2H_horse
Or13c9_mouse
Or13c9_rat
OR13C1_cow
OR13C1_dog
OR13C1_horse
OR13C1P_orang
OR13C1BP_orang
OR13C1_chimp
OR13C1P_human
OR51E1_human
0.2

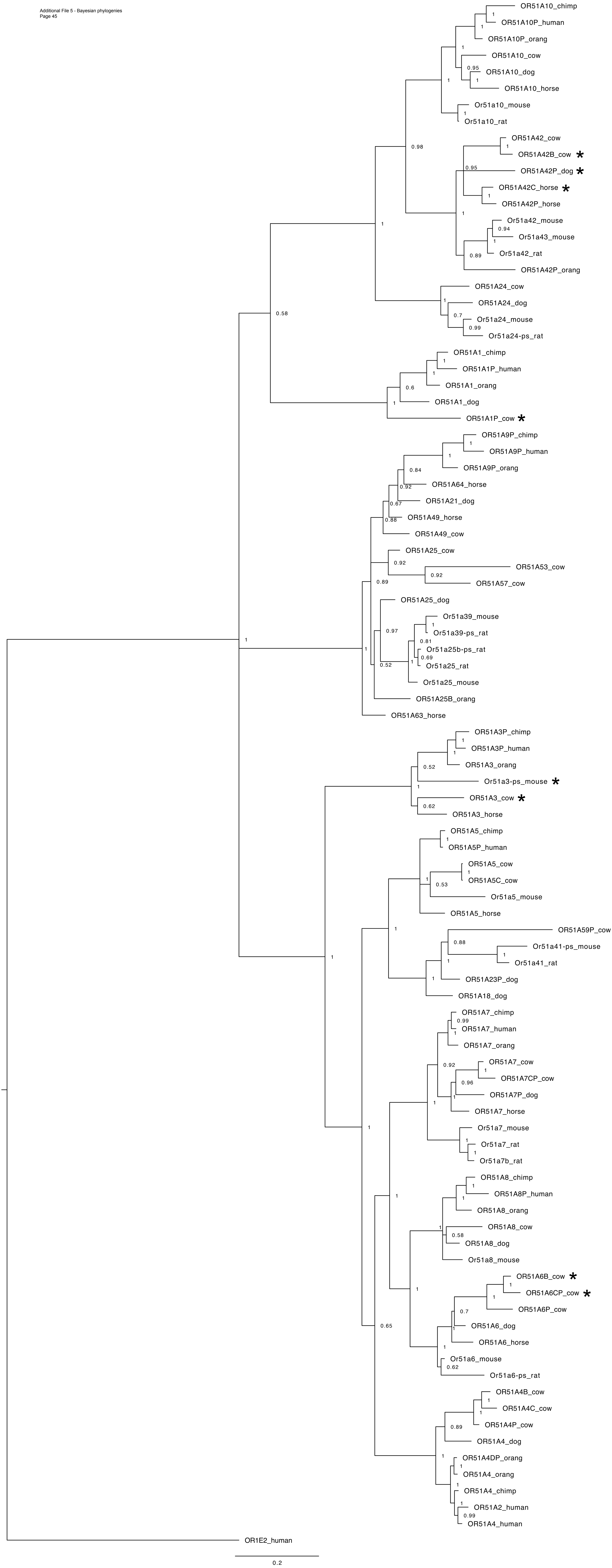
OR51A10_chimp
OR51A10P_human
OR51A10P_orang
OR51A10_cow
OR51A10_dog
OR51A10_horse
Or51a10_mouse
Or51a10_rat
OR51A42_cow
OR51A42B_cow *
OR51A42P_dog *
OR51A42C_horse *
OR51A42P_horse
Or51a42_mouse
Or51a43_mouse
Or51a42_rat
OR51A42P_orang
OR51A24_cow
OR51A24_dog
Or51a24_mouse
Or51a24-ps_rat
OR51A1_chimp
OR51A1P_human
OR51A1_orang
OR51A1_dog
OR51A1P_cow *
OR51A9P_chimp
OR51A9P_human
OR51A9P_orang
OR51A64_horse
OR51A21_dog
OR51A49_horse
OR51A49_cow
OR51A25_cow
OR51A53_cow
OR51A57_cow
OR51A25_dog
Or51a39_mouse
Or51a39-ps_rat
Or51a25b-ps_rat
Or51a25_rat
Or51a25_mouse
OR51A25B_orang
OR51A63_horse
OR51A3P_chimp
OR51A3P_human
OR51A3_orang
Or51a3-ps_mouse *
OR51A3_cow *
OR51A3_horse
OR51A5_chimp
OR51A5P_human
OR51A5_cow
OR51A5C_cow
Or51a5_mouse
OR51A5_horse
OR51A59P_cow
Or51a41-ps_mouse
Or51a41_rat
OR51A23P_dog
OR51A18_dog
OR51A7_chimp
OR51A7_human
OR51A7_orang
OR51A7_cow
OR51A7CP_cow
OR51A7P_dog
OR51A7_horse
Or51a7_mouse
Or51a7_rat
Or51a7b_rat
OR51A8_chimp
OR51A8P_human
OR51A8_orang
OR51A8_cow
OR51A8_dog
Or51a8_mouse
OR51A6B_cow *
OR51A6CP_cow *
OR51A6P_cow
OR51A6_dog
OR51A6_horse
Or51a6_mouse
Or51a6-ps_rat
OR51A4B_cow
OR51A4C_cow
OR51A4P_cow
OR51A4_dog
OR51A4DP_orang
OR51A4_orang
OR51A4_chimp
OR51A2_human
OR51A4_human
OR1E2_human
0.2

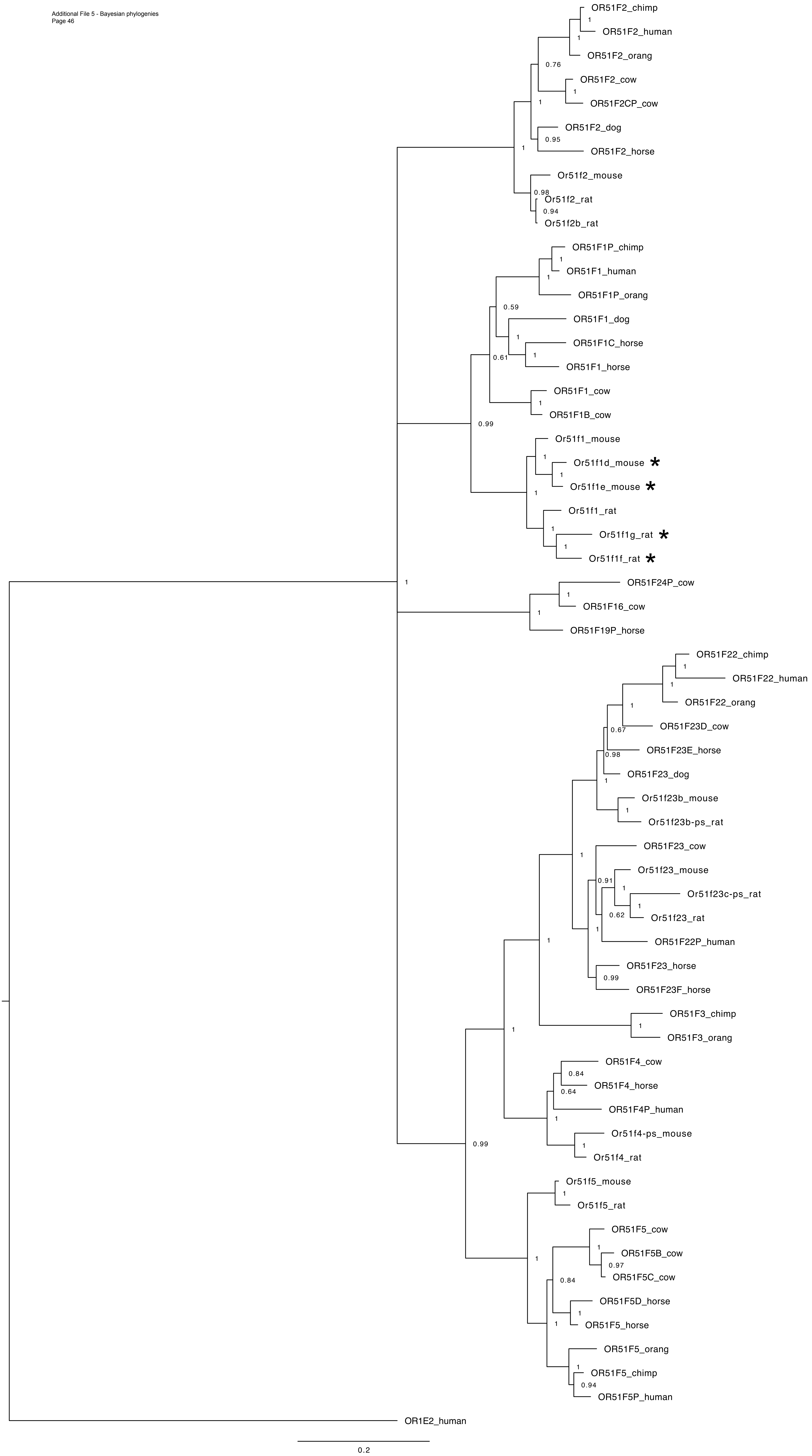
OR51F2_chimp
OR51F2_human
OR51F2_orang
OR51F2_cow
OR51F2CP_cow
OR51F2_dog
OR51F2_horse
Or51f2_mouse
Or51f2_rat
Or51f2b_rat
OR51F1P_chimp
OR51F1_human
OR51F1P_orang
OR51F1_dog
OR51F1C_horse
OR51F1_horse
OR51F1_cow
OR51F1B_cow
Or51f1_mouse
Or51f1d_mouse *
Or51f1e_mouse *
Or51f1_rat
Or51f1g_rat *
Or51f1f_rat *
OR51F24P_cow
OR51F16_cow
OR51F19P_horse
OR51F22_chimp
OR51F22_human
OR51F22_orang
OR51F23D_cow
OR51F23E_horse
OR51F23_dog
Or51f23b_mouse
Or51f23b-ps_rat
OR51F23_cow
Or51f23_mouse
Or51f23c-ps_rat
Or51f23_rat
OR51F22P_human
OR51F23_horse
OR51F23F_horse
OR51F3_chimp
OR51F3_orang
OR51F4_cow
OR51F4_horse
OR51F4P_human
Or51f4-ps_mouse
Or51f4_rat
Or51f5_mouse
Or51f5_rat
OR51F5_cow
OR51F5B_cow
OR51F5C_cow
OR51F5D_horse
OR51F5_horse
OR51F5_orang
OR51F5_chimp
OR51F5P_human
OR1E2_human
0.2

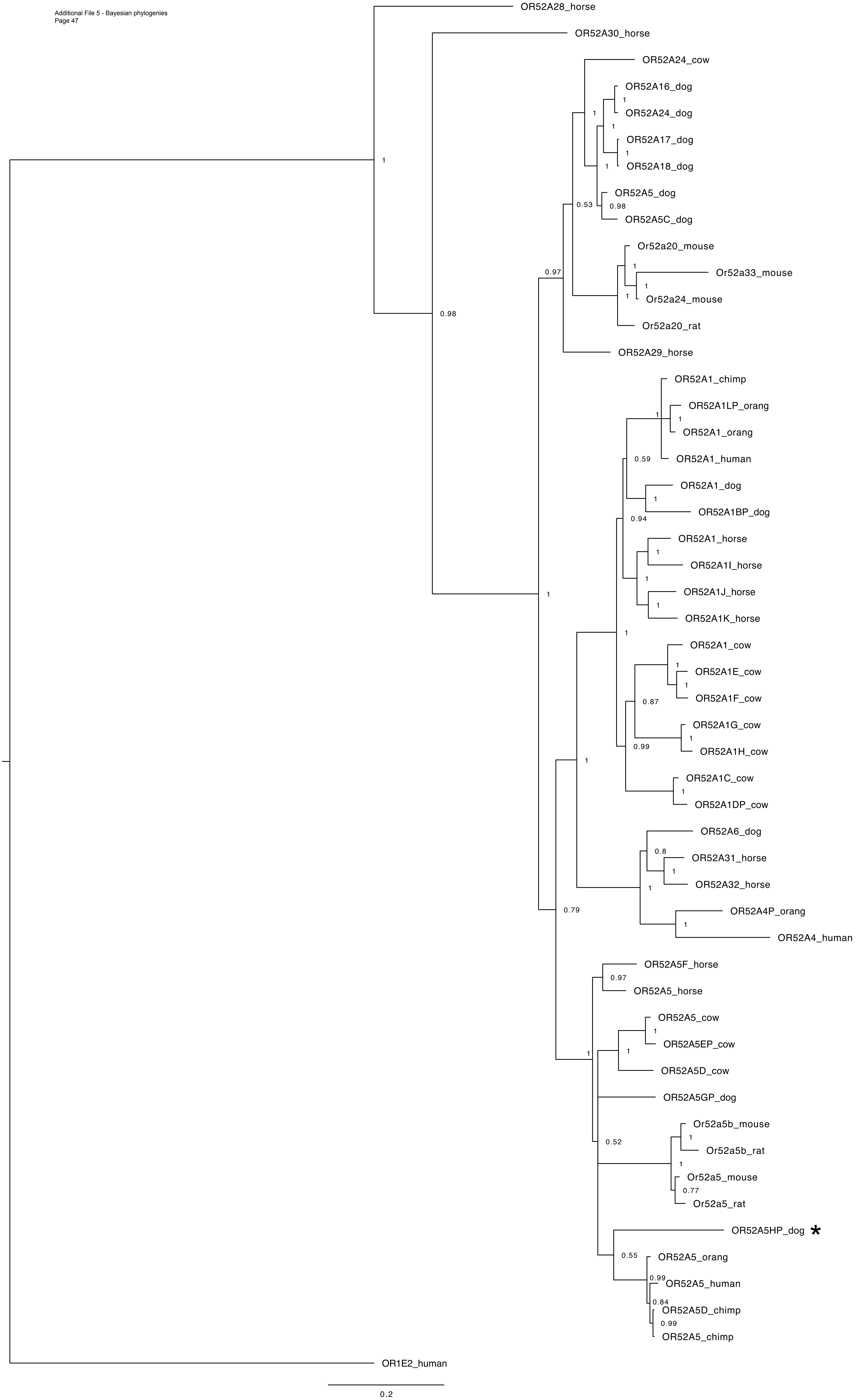
OR52A28_horse
OR52A30_horse
OR52A24_cow
OR52A16_dog
OR52A24_dog
OR52A17_dog
OR52A18_dog
OR52A5_dog
OR52A5C_dog
Or52a20_mouse
Or52a33_mouse
Or52a24_mouse
Or52a20_rat
OR52A29_horse
OR52A1_chimp
OR52A1LP_orang
OR52A1_orang
OR52A1_human
OR52A1_dog
OR52A1BP_dog
OR52A1_horse
OR52A1I_horse
OR52A1J_horse
OR52A1K_horse
OR52A1_cow
OR52A1E_cow
OR52A1F_cow
OR52A1G_cow
OR52A1H_cow
OR52A1C_cow
OR52A1DP_cow
OR52A6_dog
OR52A31_horse
OR52A32_horse
OR52A4P_orang
OR52A4_human
OR52A5F_horse
OR52A5_horse
OR52A5_cow
OR52A5EP_cow
OR52A5D_cow
OR52A5GP_dog
Or52a5b_mouse
Or52a5b_rat
Or52a5_mouse
Or52a5_rat
OR52A5HP_dog *
OR52A5_orang
OR52A5_human
OR52A5D_chimp
OR52A5_chimp
OR1E2_human
0.2

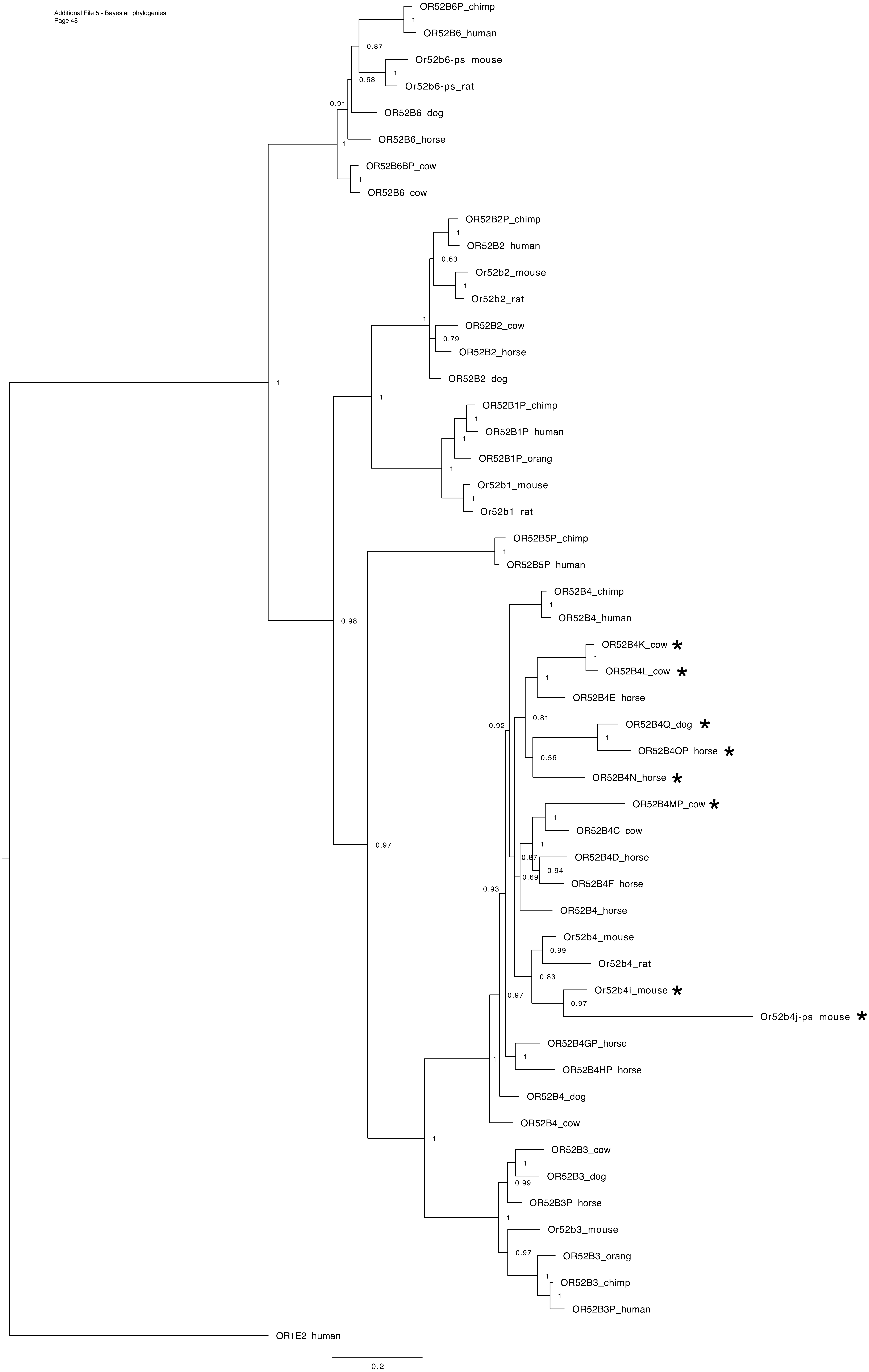
OR52B6P_chimp
OR52B6_human
Or52b6-ps_mouse
Or52b6-ps_rat
OR52B6_dog
OR52B6_horse
OR52B6BP_cow
OR52B6_cow
OR52B2P_chimp
OR52B2_human
Or52b2_mouse
Or52b2_rat
OR52B2_cow
OR52B2_horse
OR52B2_dog
OR52B1P_chimp
OR52B1P_human
OR52B1P_orang
Or52b1_mouse
Or52b1_rat
OR52B5P_chimp
OR52B5P_human
OR52B4_chimp
OR52B4_human
OR52B4K_cow *
OR52B4L_cow *
OR52B4E_horse
OR52B4Q_dog *
OR52B4OP_horse *
OR52B4N_horse *
OR52B4MP_cow *
OR52B4C_cow
OR52B4D_horse
OR52B4F_horse
OR52B4_horse
Or52b4_mouse
Or52b4_rat
Or52b4i_mouse *
Or52b4j-ps_mouse *
OR52B4GP_horse
OR52B4HP_horse
OR52B4_dog
OR52B4_cow
OR52B3_cow
OR52B3_dog
OR52B3P_horse
Or52b3_mouse
OR52B3_orang
OR52B3_chimp
OR52B3P_human
OR1E2_human
0.2

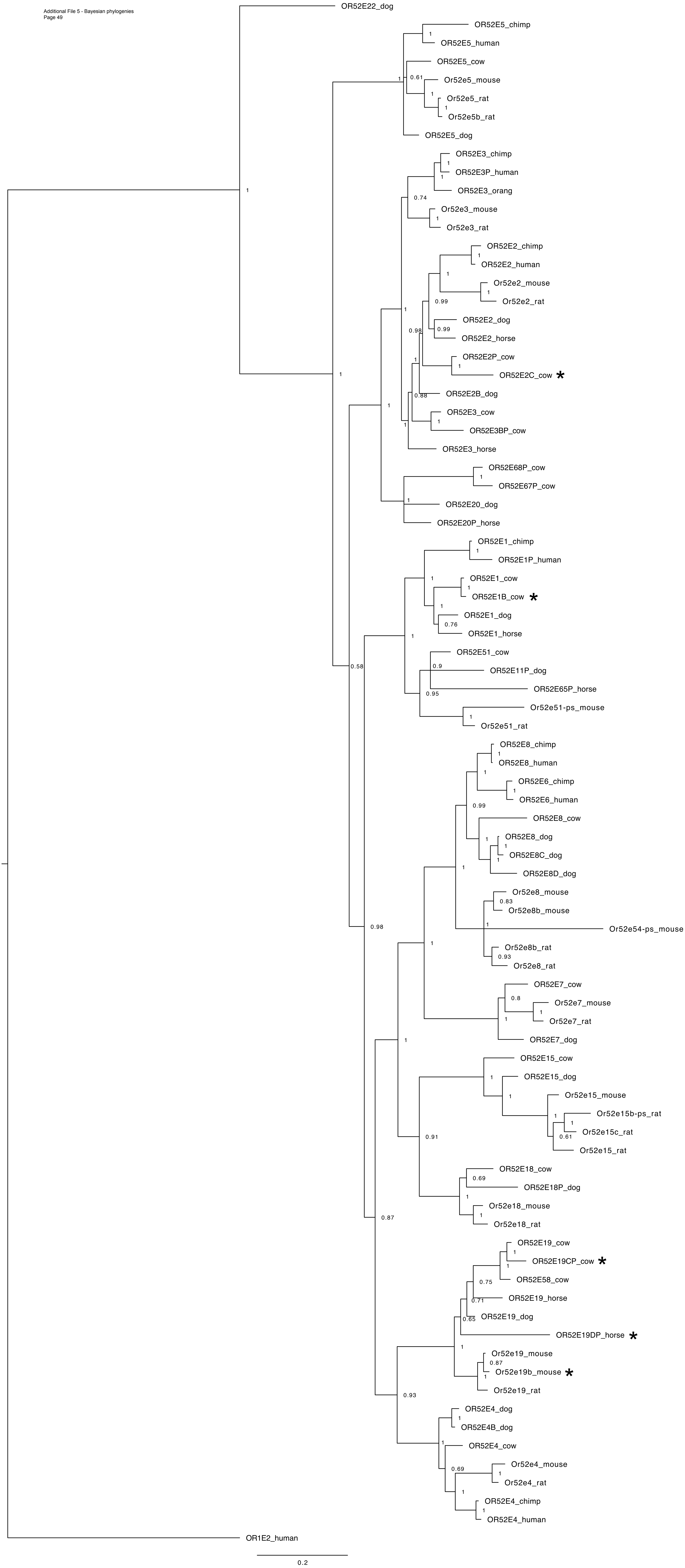
OR52E22_dog
OR52E5_chimp
OR52E5_human
OR52E5_cow
Or52e5_mouse
Or52e5_rat
Or52e5b_rat
OR52E5_dog
OR52E3_chimp
OR52E3P_human
OR52E3_orang
Or52e3_mouse
Or52e3_rat
OR52E2_chimp
OR52E2_human
Or52e2_mouse
Or52e2_rat
OR52E2_dog
OR52E2_horse
OR52E2P_cow
OR52E2C_cow *
OR52E2B_dog
OR52E3_cow
OR52E3BP_cow
OR52E3_horse
OR52E68P_cow
OR52E67P_cow
OR52E20_dog
OR52E20P_horse
OR52E1_chimp
OR52E1P_human
OR52E1_cow
OR52E1B_cow *
OR52E1_dog
OR52E1_horse
OR52E51_cow
OR52E11P_dog
OR52E65P_horse
Or52e51-ps_mouse
Or52e51_rat
OR52E8_chimp
OR52E8_human
OR52E6_chimp
OR52E6_human
OR52E8_cow
OR52E8_dog
OR52E8C_dog
OR52E8D_dog
Or52e8_mouse
Or52e8b_mouse
Or52e54-ps_mouse
Or52e8b_rat
Or52e8_rat
OR52E7_cow
Or52e7_mouse
Or52e7_rat
OR52E7_dog
OR52E15_cow
OR52E15_dog
Or52e15_mouse
Or52e15b-ps_rat
Or52e15c_rat
Or52e15_rat
OR52E18_cow
OR52E18P_dog
Or52e18_mouse
Or52e18_rat
OR52E19_cow
OR52E19CP_cow *
OR52E58_cow
OR52E19_horse
OR52E19_dog
OR52E19DP_horse *
Or52e19_mouse
Or52e19b_mouse *
Or52e19_rat
OR52E4_dog
OR52E4B_dog
OR52E4_cow
Or52e4_mouse
Or52e4_rat
OR52E4_chimp
OR52E4_human
OR1E2_human
0.2

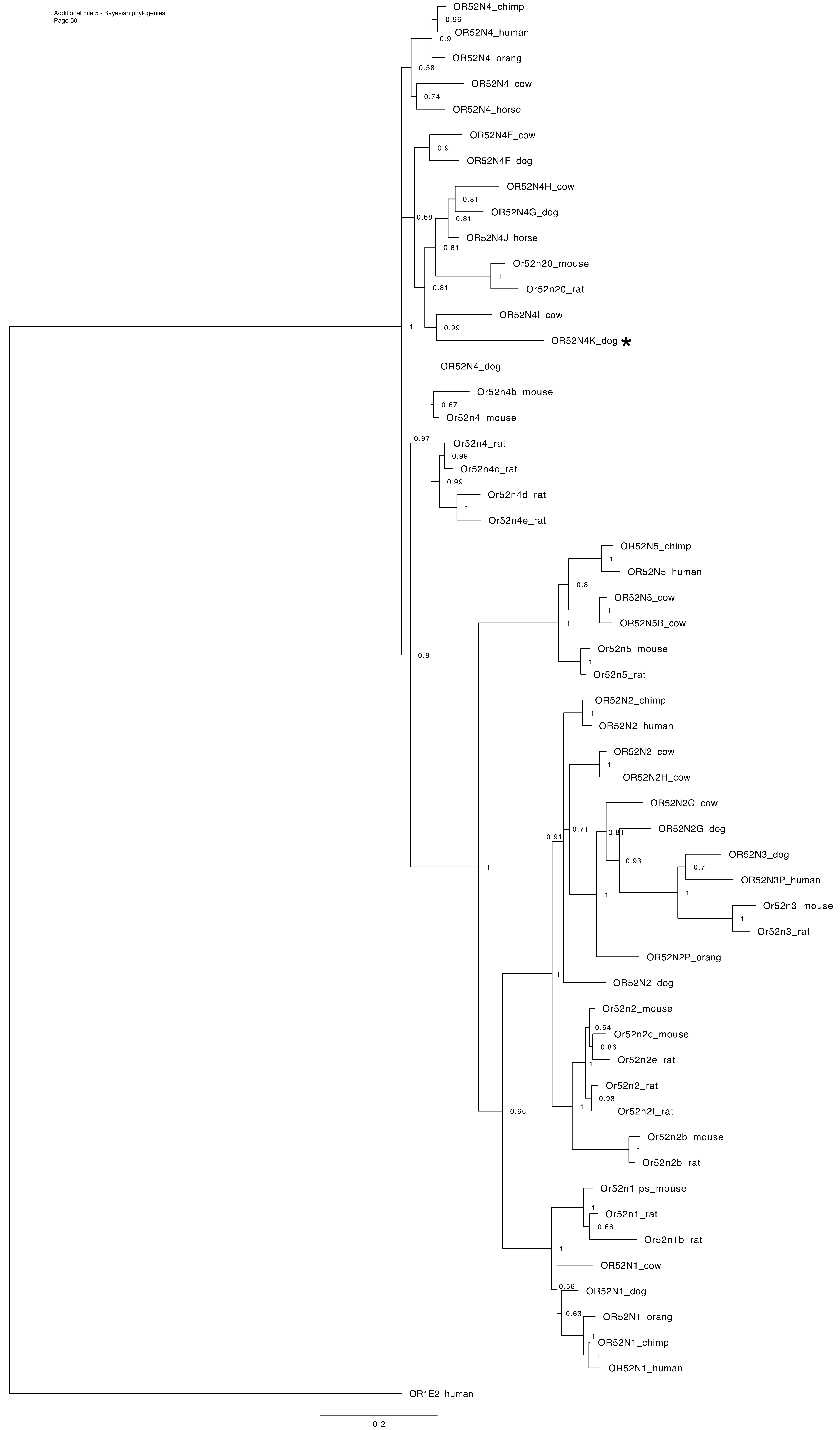
OR52N4_chimp
0.96
OR52N4_human
0.9
OR52N4_orang
0.58
OR52N4_cow
0.74
OR52N4_horse
OR52N4F_cow
0.9
OR52N4F_dog
OR52N4H_cow
0.81
OR52N4G_dog
0.68
0.81
OR52N4J_horse
0.81
Or52n20_mouse
1
Or52n20_rat
0.81
OR52N4I_cow
1
0.99
OR52N4K_dog *
OR52N4_dog
Or52n4b_mouse
0.67
Or52n4_mouse
0.97
Or52n4_rat
0.99
Or52n4c_rat
0.99
Or52n4d_rat
1
Or52n4e_rat
OR52N5_chimp
1
OR52N5_human
0.8
OR52N5_cow
1
OR52N5B_cow
1
Or52n5_mouse
0.81
1
Or52n5_rat
OR52N2_chimp
1
OR52N2_human
OR52N2_cow
1
OR52N2H_cow
OR52N2G_cow
0.91
0.71
0.81
OR52N2G_dog
0.93
OR52N3_dog
1
0.7
OR52N3P_human
1
1
Or52n3_mouse
1
Or52n3_rat
OR52N2P_orang
1
OR52N2_dog
Or52n2_mouse
0.64
Or52n2c_mouse
0.86
Or52n2e_rat
1
Or52n2_rat
0.93
1
Or52n2f_rat
0.65
Or52n2b_mouse
1
Or52n2b_rat
Or52n1-ps_mouse
1
Or52n1_rat
0.66
Or52n1b_rat
1
OR52N1_cow
0.56
OR52N1_dog
0.63
OR52N1_orang
1
OR52N1_chimp
1
OR52N1_human
OR1E2_human
0.2

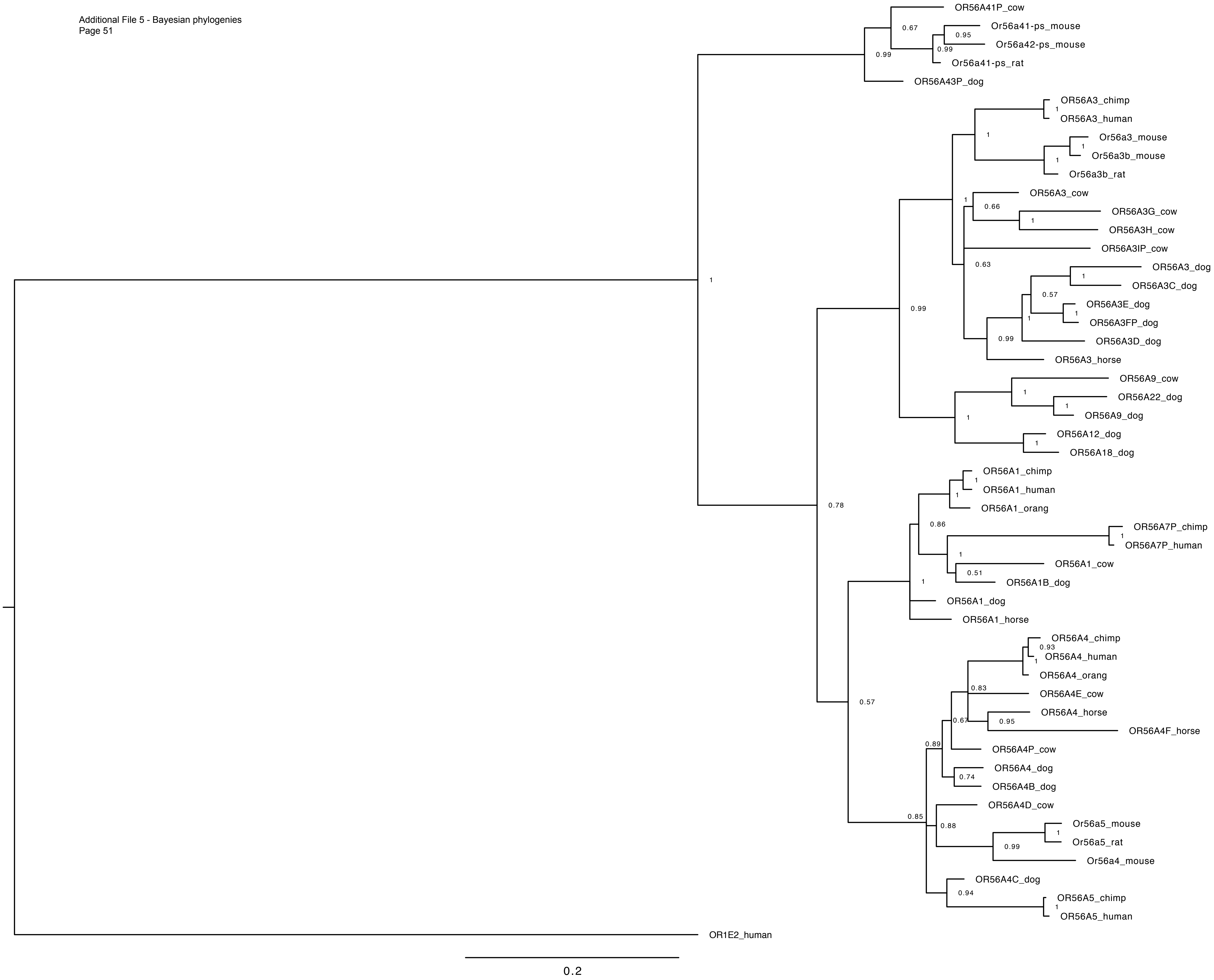
OR56A41P_cow
Or56a41-ps_mouse
Or56a42-ps_mouse
Or56a41-ps_rat
OR56A43P_dog
OR56A3_chimp
OR56A3_human
Or56a3_mouse
Or56a3b_mouse
Or56a3b_rat
OR56A3_cow
OR56A3G_cow
OR56A3H_cow
OR56A3IP_cow
OR56A3_dog
OR56A3C_dog
OR56A3E_dog
OR56A3FP_dog
OR56A3D_dog
OR56A3_horse
OR56A9_cow
OR56A22_dog
OR56A9_dog
OR56A12_dog
OR56A18_dog
OR56A1_chimp
OR56A1_human
OR56A1_orang
OR56A7P_chimp
OR56A7P_human
OR56A1_cow
OR56A1B_dog
OR56A1_dog
OR56A1_horse
OR56A4_chimp
OR56A4_human
OR56A4_orang
OR56A4E_cow
OR56A4_horse
OR56A4F_horse
OR56A4P_cow
OR56A4_dog
OR56A4B_dog
OR56A4D_cow
Or56a5_mouse
Or56a5_rat
Or56a4_mouse
OR56A4C_dog
OR56A5_chimp
OR56A5_human
OR1E2_human
0.2

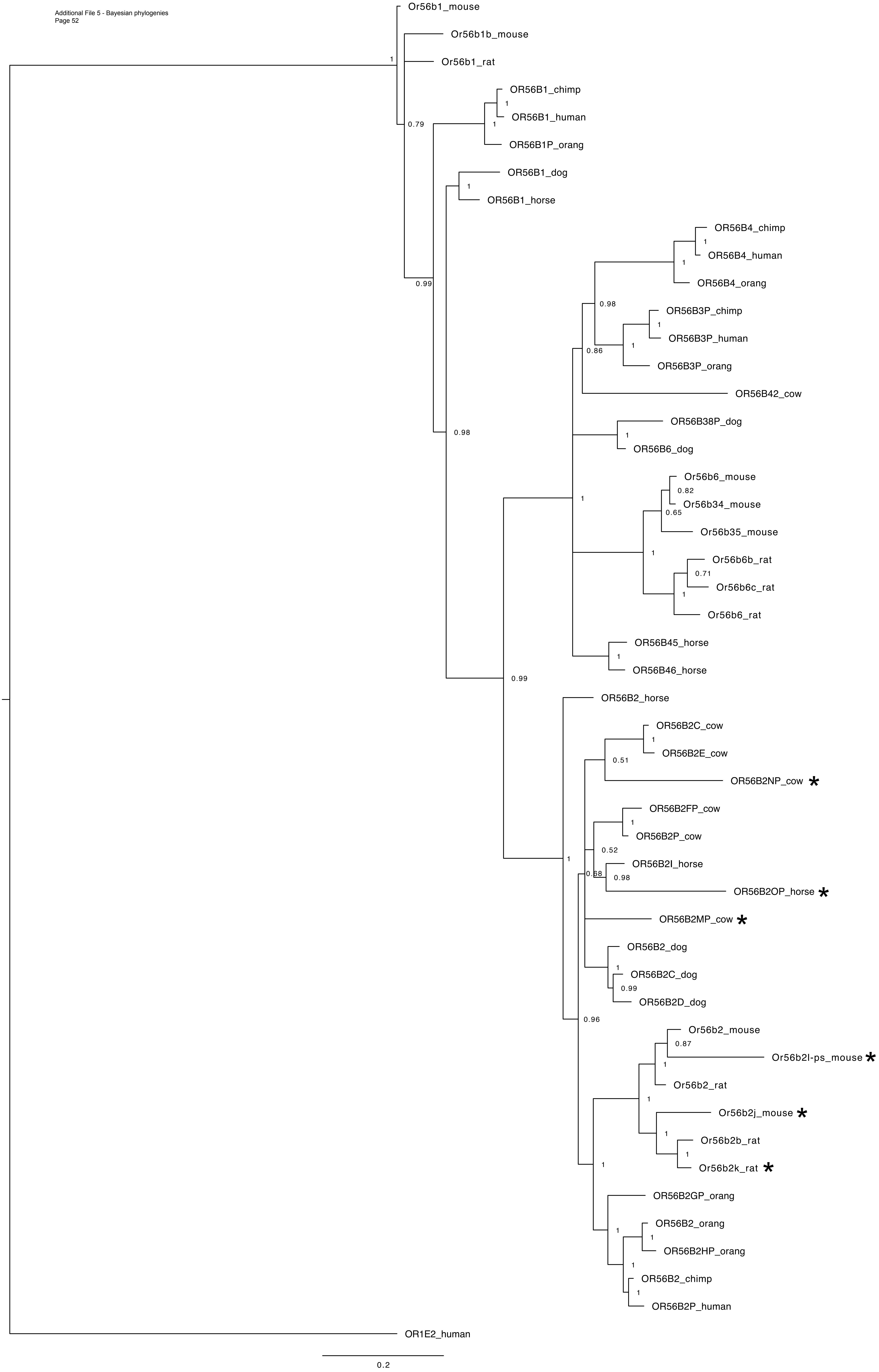
Or56b1_mouse
Or56b1b_mouse
Or56b1_rat
OR56B1_chimp
OR56B1_human
OR56B1P_orang
OR56B1_dog
OR56B1_horse
OR56B4_chimp
OR56B4_human
OR56B4_orang
OR56B3P_chimp
OR56B3P_human
OR56B3P_orang
OR56B42_cow
OR56B38P_dog
OR56B6_dog
Or56b6_mouse
Or56b34_mouse
Or56b35_mouse
Or56b6b_rat
Or56b6c_rat
Or56b6_rat
OR56B45_horse
OR56B46_horse
OR56B2_horse
OR56B2C_cow
OR56B2E_cow
OR56B2NP_cow *
OR56B2FP_cow
OR56B2P_cow
OR56B2I_horse
OR56B2OP_horse *
OR56B2MP_cow *
OR56B2_dog
OR56B2C_dog
OR56B2D_dog
Or56b2_mouse
Or56b2l-ps_mouse *
Or56b2_rat
Or56b2j_mouse *
Or56b2b_rat
Or56b2k_rat *
OR56B2GP_orang
OR56B2_orang
OR56B2HP_orang
OR56B2_chimp
OR56B2P_human
OR1E2_human
0.2
